# Shaping older adults’ care policy: a scoping review of key determinants in post-acute and community reintegration transitions

**DOI:** 10.1186/s12913-025-13433-x

**Published:** 2025-09-30

**Authors:** Saina Sehatkarlangrodi, Manaf Zargoush, Christopher J. Longo, Somayeh Ghazalbash

**Affiliations:** Health Policy and Management, DeGroote School of Business, McMaster University, 1280 Main St. W, Hamilton, ON L8S 4M4 Canada

**Keywords:** Older adults, Care transitions, Post-acute care, Community reintegration, Long-term care, Complex continuing care, Home care, Transition determinants, Scoping review

## Abstract

**Background:**

The aging population is driving an increasing demand for long-term care (LTC) and complex continuing care (CCC) across member countries of the Organization for Economic Co-operation and Development (OECD). Addressing this growing need requires improved care transition strategies that prioritize directing older adults with less resource-intensive needs toward home care rather than LTC or CCC. Effective implementation of these strategies necessitates that decision-makers have a comprehensive understanding of the factors influencing older adults’ transitions across care settings. Although substantial research has investigated these factors, the current understanding remains fragmented due to the limited synthesis of recent evidence.

**Objectives:**

This scoping review identifies factors that influence older adults’ transitions across two critical pathways: (1) post-acute care transitions (from acute care to LTC, CCC, and home care) and (2) community reintegration transitions (from LTC and CCC to home care). The findings aim to inform evidence-based integration of these factors into care models and placement decisions.

**Methods:**

Using Arksey and O’Malley’s five-stage framework, we reviewed English-language publications from OECD countries between 2015 and 2025 across SCOPUS, MEDLINE, OVID EMBASE, CINAHL EBSCO, and Web of Science.

**Results:**

Our review of 120 publications identified socio-demographic characteristics, caregiver support, health conditions, healthcare system attributes, funding policies, and person-centered care as key determinants of older adults’ transitions.

**Conclusions:**

Our review underscores the importance of incorporating the identified determinants into care models to address older adults’ individualized needs and support optimal placement decisions. This evidence-based approach can guide policy reforms and management practices, improving resource utilization and system efficiency. Additionally, we outline key gaps in the literature and propose directions for future research.

**Supplementary Information:**

The online version contains supplementary material available at 10.1186/s12913-025-13433-x.

## Background

Over the past decade, Organization for Economic Co-operation and Development (OECD) countries have experienced a dramatic demographic shift, with the proportion of individuals aged 65 and over rising from 14.61% in 2010 to 17.96% in 2021 [1]. This aging population increasingly faces chronic health conditions, functional limitations, and frailty that compromise independent living, necessitating sustained clinical, personal, and social care services [2, 3]. These services, designed to address older adults’ ongoing care needs, are primarily delivered across three settings. Long-Term Care (LTC), commonly called nursing homes or residential care homes in various OECD countries, provides round-the-clock supervision and personal assistance for extended periods [4, 5]. Complex Continuing Care (CCC), also known as skilled nursing facilities or geriatric rehabilitation units, delivers intensive medical, nursing, and rehabilitative care to individuals with complex chronic conditions [6]. For the purposes of this review, LTC and CCC are collectively referred to as ‘institutional care.’ Lastly, Home Care services provide personal and social support to older adults within the comfort and familiarity of their own homes [7].

In recent decades, these care settings have faced mounting challenges [8]. The rapid growth of the aging population in OECD countries has intensified the demand for institutional care, resulting in patient flow congestion [9, 10]. For example, in Ontario, Canada’s largest province, the LTC waitlist has doubled over the past decade [11, 12]. The COVID-19 pandemic has further exacerbated these pressures through service backlogs [13]. Similar patterns are evident across other OECD countries. In the UK, more than 14,000 acute care (hospital) beds per day were occupied in 2024 by patients who were medically fit for discharge but lacked access to appropriate continuing care, creating a system-wide bottleneck [14]. In the United States, institutional care occupancy had increased, while persistent staffing shortages led many facilities to cap new admissions [15, 16].

Some countries have attempted to address these patient flow disruptions by expanding institutional bed capacity [11, 17–19]. However, this approach is increasingly viewed as impractical due to the projected doubling of the older adult population over the next two decades [20–22]. Meeting this growth would require doubling the bed capacity, a financially unsustainable solution [11]. As a result, some jurisdictions have introduced more controversial measures. In Ontario, for example, Bill 7 allows for the involuntary placement of older adults in non-agreed settings. This policy has been criticized as both discriminatory and suboptimal [23]. Similarly, in the UK, a policy aimed at easing hospital pressures involved block-booking LTC beds to accommodate medically stable patients [24, 25]. In practice, this enabled transfers to institutional settings without patients’ informed consent, prompting legal and ethical objections from sector leaders [25]. Germany offers another example of systemic burden-shifting. Under its statutory LTC insurance model, cost-sharing structures have placed significant financial pressure on individuals and families. In some cases, adult children are legally obligated to contribute to LTC expenses [26–28]. Taken together, these examples highlight how transferring responsibility for continuing care from public systems to individuals can undermine both autonomy and equity.

A more sustainable solution, endorsed by academics and clinicians, involves leveraging the underutilized capacity of home care to reduce the strain on LTC and CCC facilities [29, 30]. This strategy proposes directing older adults who require less resource-intensive care to home settings, thereby preserving critical institutional resources for those with more complex needs. Even small reductions in residents’ length of stay in institutional settings can significantly improve patient flow [20]. For example, in a system with 30,000 LTC beds, reducing each new resident’s length of stay by just one month could enable the system to serve 1,000 more individuals within the existing capacity [20]. Furthermore, home care aligns with older adults’ preferences, as most seniors prefer to live independently in their own homes [31–35]. Overall, this growing body of evidence has prompted policymakers and decision-makers to reevaluate transitions, emphasizing the need to direct older adults requiring lower levels of care to home care rather than LTC and CCC [29].

**Fig. 1:**
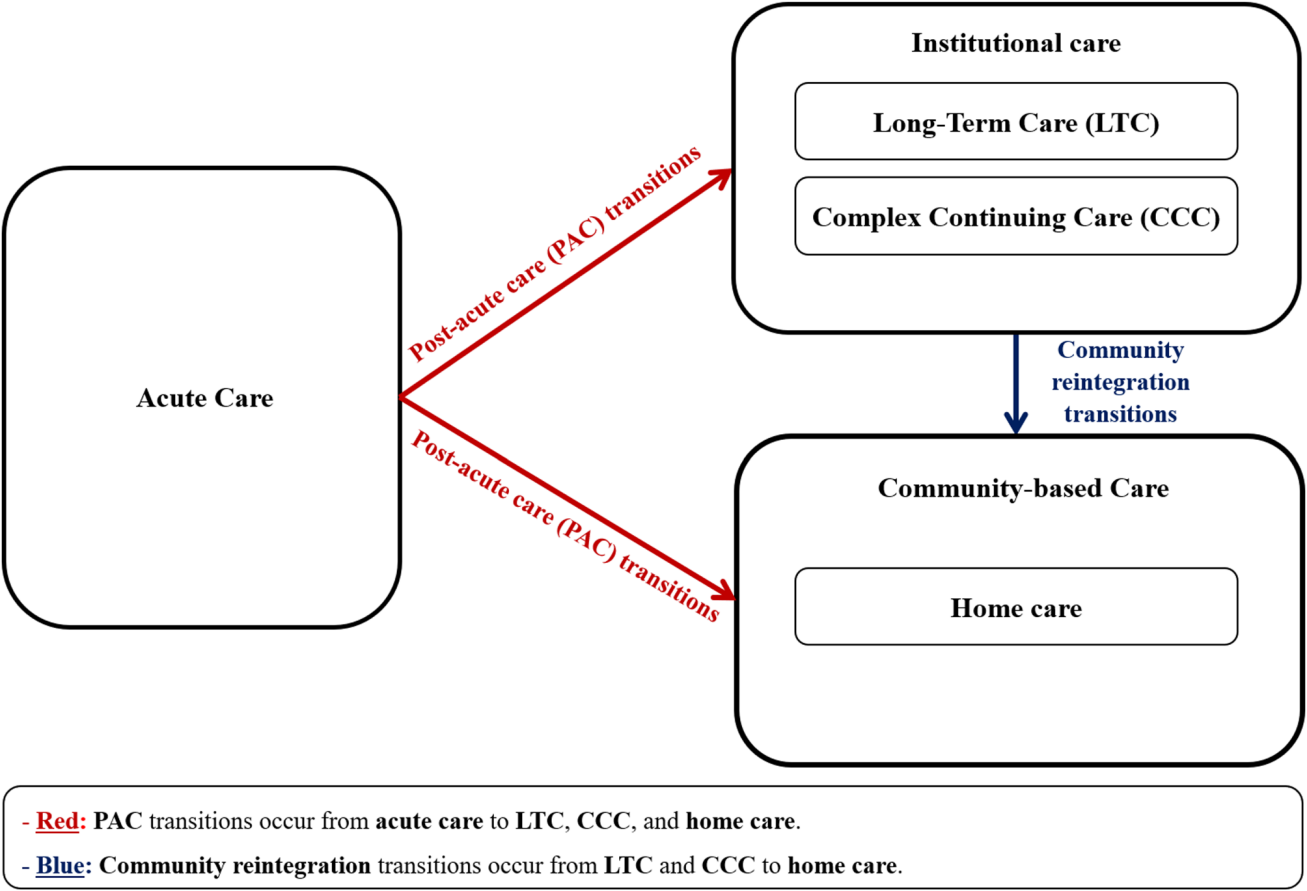
Types of transitions considered within the scope of this study

An effective implementation of this strategy hinges on a thorough understanding of the factors influencing older adults’ transitions to LTC, CCC, and home care. Such knowledge can provide decision-makers with actionable insights for placing older adults in suitable care environments based on their individual conditions and characteristics. Among various possible care transitions, this study focuses on two critical pathways that considerably impact system efficiency and patient outcomes. The first pathway represents transitions from acute care to LTC, CCC, and home care, namely “post-acute care (PAC) transitions.” In OECD countries, these represent the dominant route into institutional settings and frequently create bottlenecks due to capacity constraints in LTC and CCC [19–21]. The second pathway represents transitions from LTC and CCC back to community living through home care, namely “community reintegration transitions.” While less common, these transitions are becoming increasingly important. They help alleviate the pressure on LTC and CCC and demonstrate improved quality of life for older adults who successfully return home [11, 36, 37]. Overall, as Fig. 1 depicts, this study examines both PAC and community reintegration transitions.

The existing literature reviews on the factors influencing these transitions are largely based on pre-2015 studies and provide a limited perspective. While they examine fundamental socio-demographic factors (e.g., age, gender, and marital status), they often overlook complex population attributes (e.g., socio-economic status, education, and housing) and modifiable healthcare system characteristics (e.g., medical interventions, facility capacity, and staffing) [38, 39].

Additionally, given the aging population and regulatory adjustments to healthcare systems due to staffing and funding constraints, pre-2015 studies may not accurately reflect the current landscape [40]. This discrepancy is further magnified by recent shifts in the healthcare landscape due to the COVID-19 pandemic [41]. Finally, there is a lack of synthesis that takes an integrated approach to examining factors across both PAC and community reintegration pathways. Consequently, there is a clear need for an updated review to provide a more accurate and nuanced understanding of the factors shaping these transitions.

This study aims to conduct a scoping review to identify, examine, and synthesize the existing knowledge on these factors within OECD countries, addressing the following research question: “What factors are associated with timely and needs-appropriate PAC and community reintegration transitions among older adults in OECD countries?” Our primary objective is to provide policymakers and decision-makers with a comprehensive understanding of the key determinants that influence whether these transitions occur at the right time and in alignment with individuals’ care needs. These determinants can be integrated into care models, transition planning protocols, and placement decisions, ultimately supporting more effective, evidence-informed care pathways. Additionally, by analyzing the self-reported limitations outlined in the included studies, this review will shed light on knowledge gaps to guide future research.

Our focus on OECD countries is deliberate. Despite the diversity in these countries’ healthcare systems, they share common demographic trends of rapidly aging populations [42, 43]. These nations face increasing pressure on their care systems, particularly in institutional settings, and grapple with similar policy challenges, including the need to enhance system efficiency and shift towards more community-based care models [44, 45]. The similarity in these overarching challenges allows for meaningful comparisons and potential transferability of insights across these countries.

## Methods

The five-stage framework outlined by Arksey and O’Malley was employed to conduct this scoping review [46]. This methodology was chosen for its ability to systematically map the diverse range of factors influencing older adults’ transitions [46]. The stages of this framework include (1) identifying the research question, (2) identifying the initial set of publications, (3) selecting relevant publications, (4) charting the data, and (5) collating, summarizing, and reporting the results. In designing and reporting this review, we followed recent methodological guidance for scoping reviews, including the Preferred Reporting Items for Systematic reviews and Meta-Analyses extension for Scoping Reviews (PRISMA‑ScR) [47], and the updated JBI scoping review guidance [48]. This review was preregistered with the Open Science Framework (OSF)^1^.

### Search strategy - identifying the initial set of publications

Five major databases, SCOPUS, MEDLINE, OVID EMBASE, CINAHL EBSCO, and Web of Science, were searched between 2015 and 2025. These databases were chosen for their global reach, extensive coverage of health sector literature, and reputation for providing high-quality evidence that informs healthcare decision-making [49]. In addition to peer-reviewed literature, practice-oriented documents such as government reports, evidence briefs, and expert commentaries were also examined to enhance the review’s comprehensiveness. The searches were limited exclusively to English-language documents.

The search terms encompassed specific keywords relevant to the field. These terms include, among others, “older adults,” “seniors,” “acute care,” “hospital*,” “transitions,” “transfers,” “long-term care,” “nursing home,” “complex continuing care,” “skilled nursing facility,” “home care,” “factors,” “determinants,” and “influences.” Table S1 in the supplementary materials provides a detailed search strategy.

### Inclusion/exclusion criteria - selecting relevant publications

Our inclusion criteria were structured using the Population-Concept-Context (PCC) framework recommended by the JBI guidance [48], focusing on older adults (population), factors influencing transitions across care settings (concept), and OECD health care systems (context). More specifically, publications were considered for inclusion if they: (1) examined transitions within at least one of the targeted pathways, namely acute care to LTC, acute care to CCC, acute care to home care, LTC to home care, or CCC to home care; (2) explicitly focused on factors influencing transitions within these pathways rather than other related topics; (3) pertained to individuals aged 65 and above, acknowledging the specialized health care needs of older adults; and (4) originated from OECD countries. We further restricted the search to publications from 2015 to 2025 and included only English-language records. We did not pre-specify separate exclusion criteria. During screening, a record was excluded if it did not meet one or more of the above inclusion conditions.

The selection process began with an initial assessment of titles and abstracts from the search results, conducted by two reviewers to identify publications aligned with the research aim. Following the initial screening, the same two reviewers conducted a detailed full-text review to confirm eligibility according to the inclusion/exclusion criteria. Instances of uncertainty were resolved through discussion and consultation with supervisory researchers.

### Data extraction

A data extraction form was employed to chart the important information from the selected resources. The fields in the form included Title, First Author, Publication Year, Analysis Period, Document Type (academic or practice-oriented), Jurisdiction, Study Aim, Study Population, Research Design, Original and Destination Care Setting, and Factors Positively/Negatively Influencing Transitions from Original Care Setting to Destination Care Setting. The extracted data were stored in Microsoft Excel, facilitating the systematic organization, categorization, and retrieval of the data to support narrative synthesis. After charting factors, one reviewer assigned descriptive labels to recurring items and conceptually grouped them into high-level categories. The categories were then reviewed with the supervisory researchers to support consistency and clarity.

## Results

**Fig. 2:**
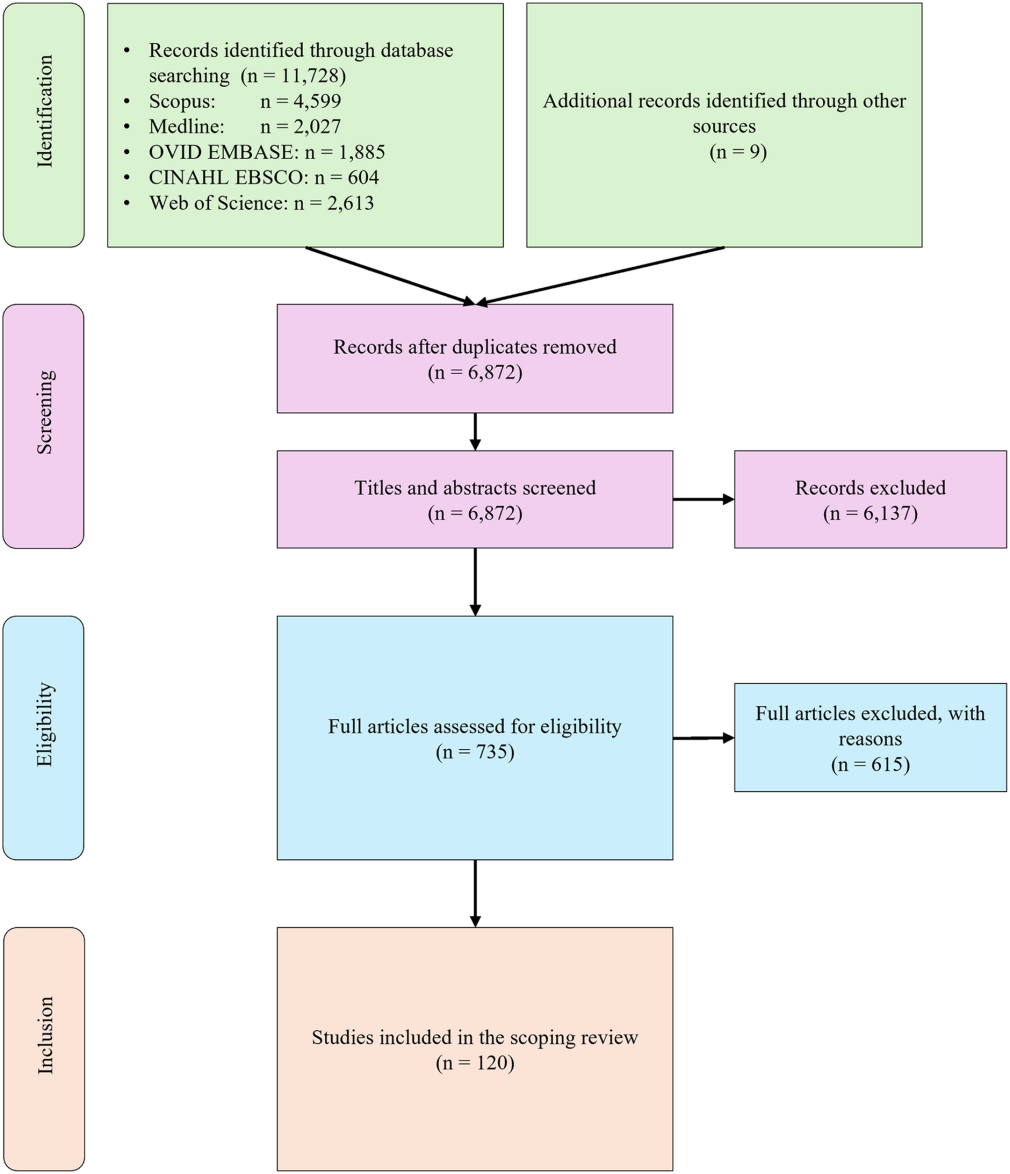
PRISMA diagram

Our database search yielded 11,728 records. After the abstract screening, we retrieved 735 records for full-text review. Of these, 120 publications were ultimately included and analyzed. Table S2 in the supplementary materials provides the complete list of included publications. Figure 2 represents the PRISMA flow diagram.

Throughout this paper, ‘n=’ denotes the number of resources. Most included academic studies utilized quantitative research design (*n* = 85), while a smaller proportion employed a qualitative approach (*n* = 12), literature review methods (*n* = 14), or mixed methods (*n* = 4). Unique designs found in practice-oriented documents included reports (*n* = 3), practice development (*n* = 1), and commentary pieces (*n* = 1).

Geographically, the collected resources spanned various jurisdictions, with a high concentration in the USA (*n* = 50), followed by Canada (*n* = 13), Japan (*n* = 11), and Australia (*n* = 8). European studies were distributed across the UK (*n* = 8), the Nordic countries (*n* = 6), Switzerland (*n* = 5), France (*n* = 2), the Netherlands (*n* = 2), Italy (*n* = 1), and Ireland (*n* = 1). Furthermore, several studies (*n* = 13) provided cross-national perspectives, covering multiple OECD countries across North America, Europe, and Asia.

Regarding the publication years, resources spanned from 2015 to 2025, peaking in 2020 (*n* = 17). This trend reflects the growing emphasis of research on older adults’ care transitions in recent years. The study populations varied significantly, with the smallest involving fewer than 20 participants and the largest encompassing a cohort of 9,762,208 individuals, reflecting the diverse scales of these investigations.

Throughout this review, we use standardized terminology for care settings to ensure consistency. LTC encompasses facilities referred to as long-term care, nursing homes, care homes, or residential care homes across different healthcare systems. CCC denotes complex continuing care, skilled nursing facilities, intermediate care facilities, sub-acute care, or geriatric rehabilitation units, depending on jurisdiction. Lastly, home care refers to health and personal support services delivered in older adults’ homes.

### Factors influencing PAC transitions

**Fig. 3:**
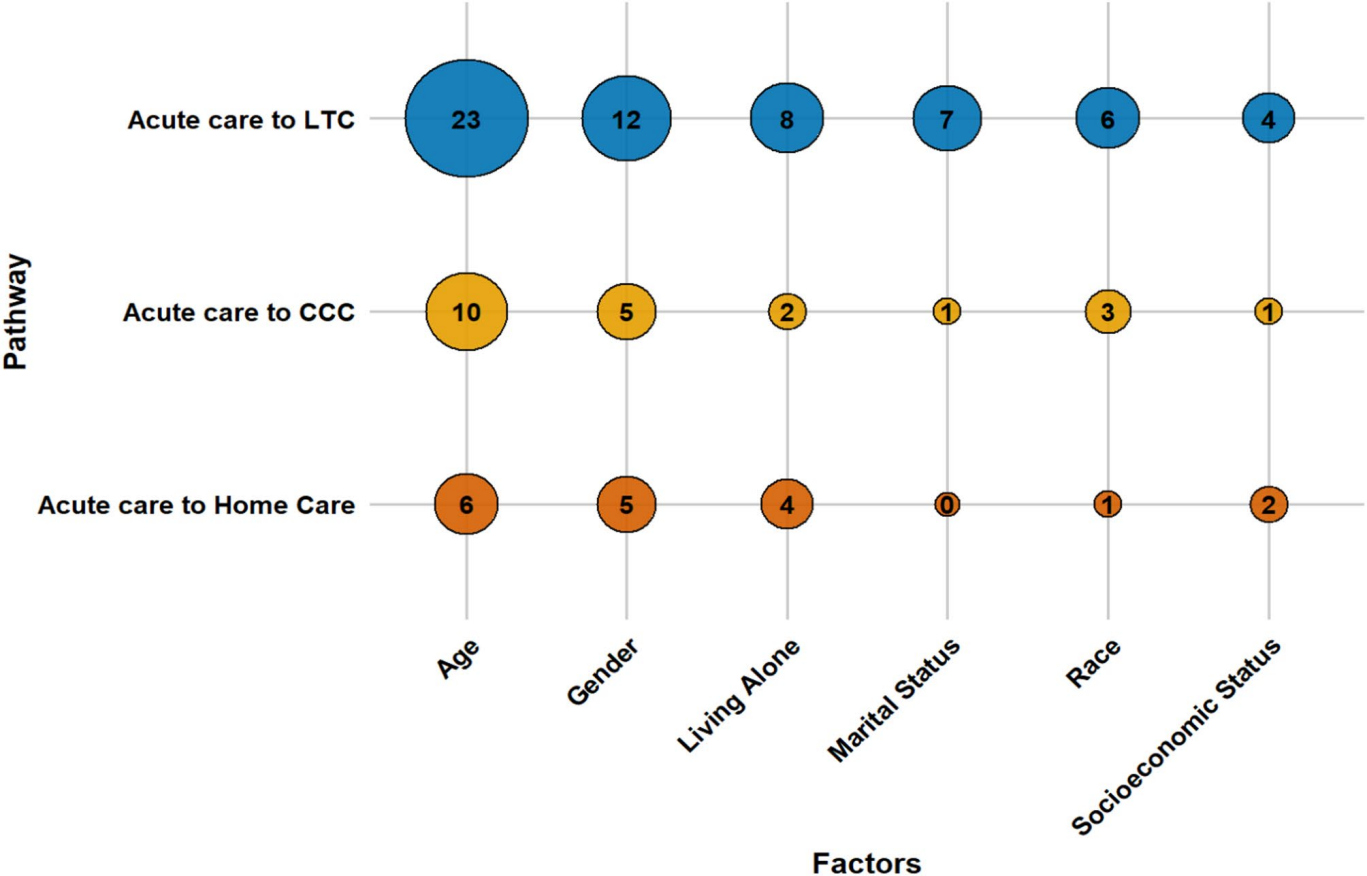
The influence of socio-demographic determinants on PAC transitions

**Fig. 4:**
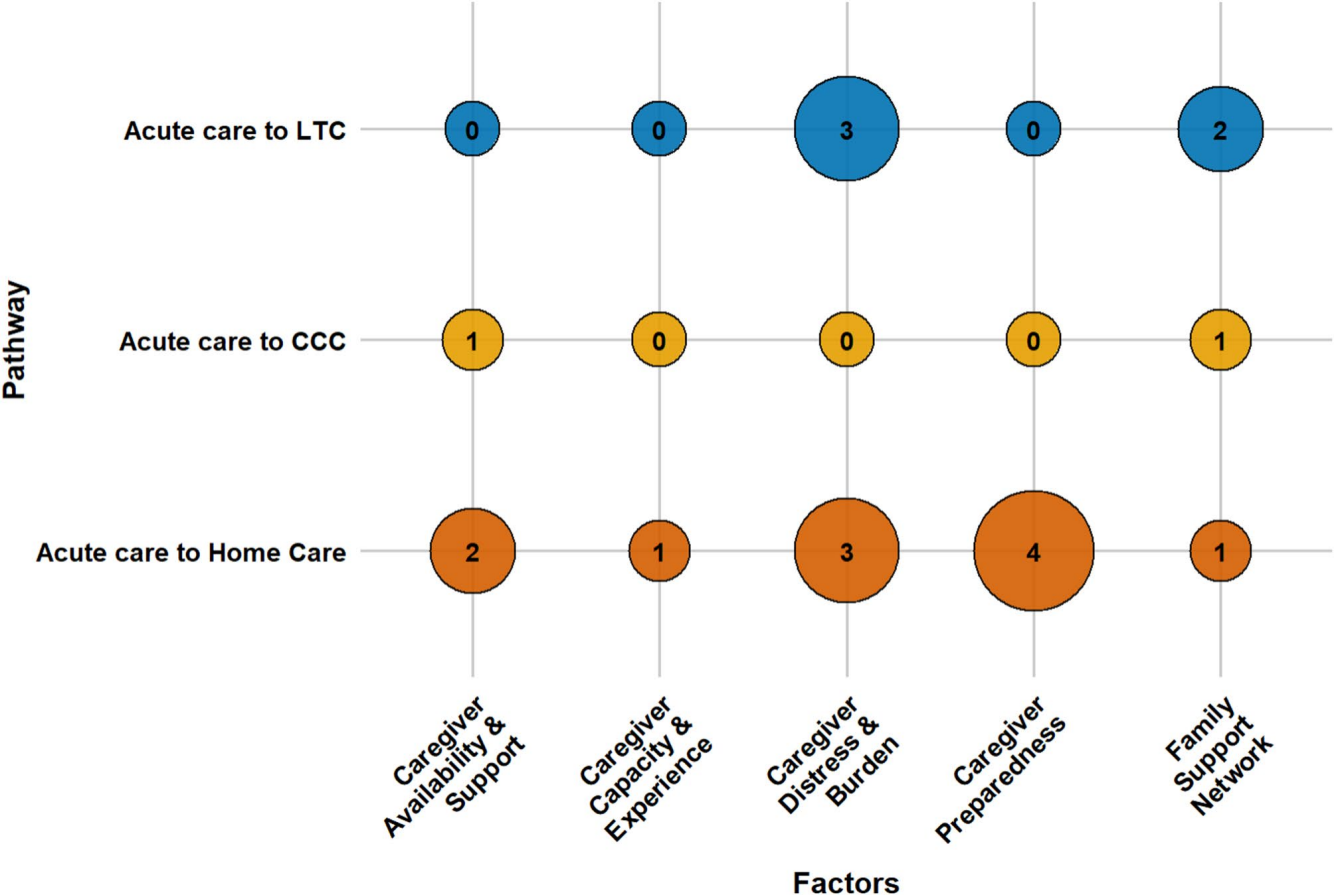
The influence of caregiver support determinants on PAC transitions

**Fig. 5:**
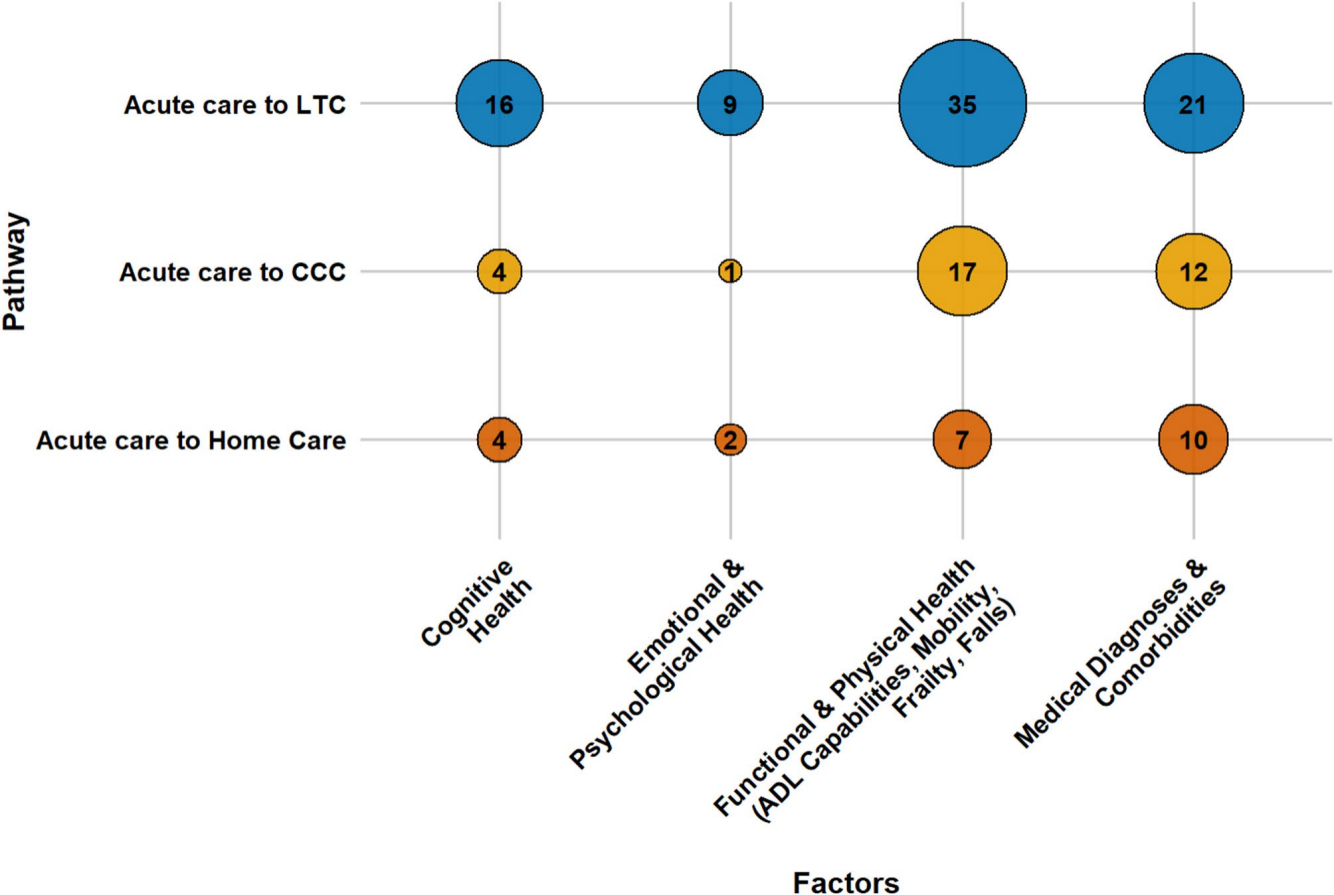
The influence of health condition determinants on PAC transitions

**Fig. 6:**
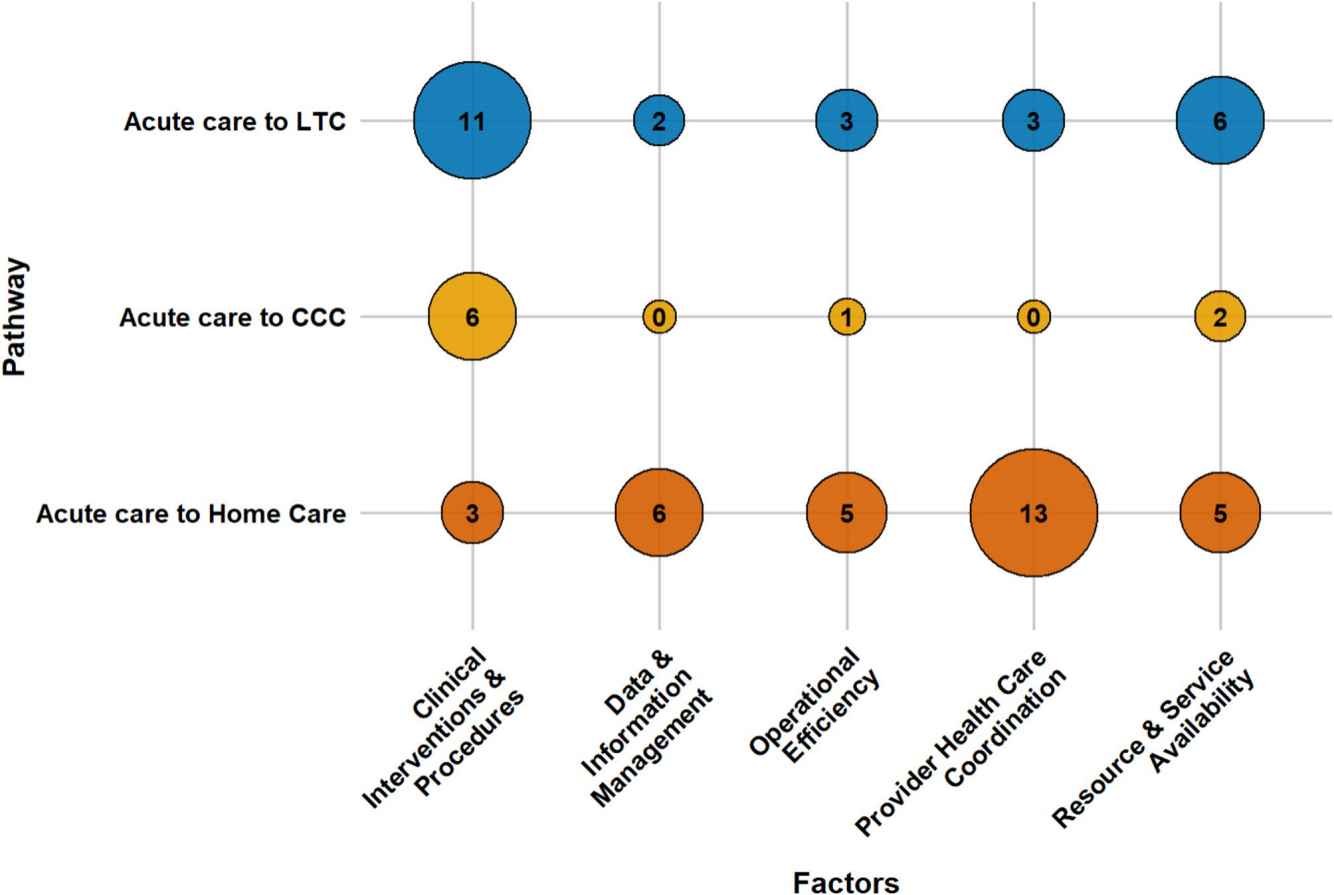
The influence of health care system determinants on PAC transitions

An array of factors influences older adults’ transitions from acute care to LTC, CCC, and home care. These determinants can be grouped into four main categories: socio-demographic characteristics, caregiver support, health conditions, and healthcare system elements. This section presents a consolidated overview of key factors across all pathways. Section S3 of the Supplementary Materials presents detailed, study-specific findings with citations, while Table S3 lists all identified factors and their frequency. Figures 3, 4, 5 and 6 offer a broad visual summary, grouping key elements into overarching categories to complement the results. These charts are based on studies with diverse designs and are scaled specifically for PAC transitions without distinguishing between methodological approaches.

#### Consolidated overview of key determinants in older adults’ PAC transitions

Across PAC pathways, socio-demographic characteristics manifest consistent yet setting-specific associations. Advanced age is the most pervasive determinant, positively associated with transitions from acute care to LTC and CCC, and inversely associated with discharge to home care. Evidence for sex differences is weaker but generally aligns in direction. Female patients are more frequently admitted to institutional care; however, a minority of studies, such as a recent analysis of hip-fracture patients, have suggested that male sex may also elevate the likelihood of LTC placement. Indicators of social isolation, such as living alone, being unmarried, widowed, or divorced, uniformly increase the probability of placement in LTC or CCC and decrease the likelihood of transitioning to home care.

Racial and ethnic patterns are heterogeneous. Black individuals are often over-represented among LTC admissions, while individuals of Asian/Pacific Islander, Hispanic, and American‑Indian/Alaska‑Native backgrounds are generally associated with lower rates of LTC entry. Evidence on CCC transitions is mixed: one study conducted during the COVID‑19 pandemic reported that non‑Latinx Black and Latinx patients were less likely to enter CCC, whereas another found higher CCC placement rates among non‑Hispanic Black and Hispanic patients. In contrast, Hispanic ethnicity is consistently associated with lower rates of LTC placements and a higher likelihood of discharge to home care.

Socioeconomic gradients are evident across all pathways. Lower household income, unaffordable service costs, housing instability, and homelessness heighten reliance on institutional options, whereas higher income confers relative protection. Qualitative evidence further indicates that language barriers limit access to home care for ethnic minorities. The quantitative evidence base is densest for LTC and comparatively sparse for CCC. Figure 3 illustrates the distribution of socio-demographic factors across pathways.

Caregiver support was a critical determinant of PAC transitions, although the extent of its documentation varied considerably across settings. In LTC transitions, caregiver strain and the inability to provide informal support increased the risk of institutionalization, while strong family networks and reliable informal caregivers served as protective factors. In CCC transitions, caregiver dynamics are less frequently studied. Limited evidence suggested that the absence of an informal caregiver raises the likelihood of CCC placement, and that involving family members in care planning facilitates these transitions.

In home care pathways, caregiver support has been examined more comprehensively. Evidence from quantitative studies showed that well-prepared and available informal caregivers facilitate successful home transitions, whereas qualitative findings revealed that insufficient readiness, burnout, or limited capacity among caregivers can impede them. Taken together, the findings indicate that caregiver capacity and readiness are central to successful home transitions, although they receive comparatively less attention in research on institutional placements. Figure 4 delineates the role of caregiver support in PAC transitions.

Health conditions were consistently found to influence PAC transitions, with distinct patterns across LTC, CCC, and home care. A higher comorbidity burden was associated with increased transitions to LTC and CCC and reduced likelihood of discharge to home care, particularly in cases involving advanced or metastatic disease. Cognitive impairment, especially dementia, is among the most frequently cited determinants in LTC transitions. In CCC settings, the influence of dementia was less consistent, with studies reporting both positive and negative associations. Better cognitive performance increases the likelihood of home care transitions. Mental health conditions were less frequently examined but showed consistent effects. Behavioral challenges, depression, and refusal of care were linked to higher rates of institutional placement and lower rates of home discharge.

Functional status was a central determinant across all pathways. Impaired mobility, activities of daily living (ADL) dependence, Falls, and general functional decline were strongly associated with LTC and CCC transitions, while higher functional capacity and mobility promoted home care transitions. Frailty was another commonly examined factor in LTC and CCC settings, with higher levels consistently associated with institutionalization. Nutritional status also shaped outcomes, with poor nutritional profiles increasing the likelihood of institutional placement.

A range of specific medical conditions also influenced transition patterns. Neurological, urological, musculoskeletal, cardiovascular, respiratory, and oncological conditions, as well as incontinence and general medical instability, were linked to higher LTC placement. In CCC transitions, cardiovascular disease was associated with a decreased likelihood of transfer, whereas chronic liver disease, spinal cord injury, major surgeries, stroke, and diabetes showed positive associations. In home care transitions, lower stroke severity, absence of fracture-related injuries, high nutritional and functional scores, and good trunk control were facilitators, while pressure ulcers, pleural effusion, and prior traumatic brain injury acted as barriers. Overall, these patterns underscore the role of cognitive, functional, and clinical complexity in shaping PAC trajectories. Figure 5 examines the influence of diverse health conditions on PAC transitions.

Healthcare system factors also shaped PAC transitions, with the nature of determinants varying by setting. Across LTC and CCC, multiple clinical procedures were associated with higher institutional placement. These included orthopedic, cardiac, and gynecological surgeries, general anesthesia, respiratory treatments, and multiple interventions during a single hospitalization. In home care pathways, some procedures such as total knee arthroplasty and intensive medical therapies reduced discharge likelihood, whereas pre-hospitalization physiotherapy consistently facilitated home transitions.

Hospital operational efficiency further influenced outcomes. Higher patient-to-nurse ratios were linked to increased LTC and CCC placements, while integrated care models, person-centered planning, and clearly defined provider roles supported successful home discharges. In contrast, poor communication between hospital teams, unclear staff responsibilities, insufficient incorporation of patient and family preferences, and resource constraints were reported as barriers across all settings, particularly for home discharges.

Information systems were key enablers in home care transitions. Health information exchange platforms, shared records, and telehealth services were consistently associated with improved continuity and discharge success. Conversely, delays in information access, fragmented communication, and lack of standardized protocols undermined effective transitions. Access to services also plays a decisive role. Limited LTC bed availability delayed discharges from acute care, while shortages in home care staffing, geographic disparities, and inequities in community-based service infrastructure posed substantial challenges to home transitions. These findings underscore the importance of aligning procedural, operational, and informational capacities to support timely and appropriate PAC planning. Figure 6 explores the influence of healthcare system determinants on PAC transitions.

### Factors influencing community reintegration transitions

**Fig. 7:**
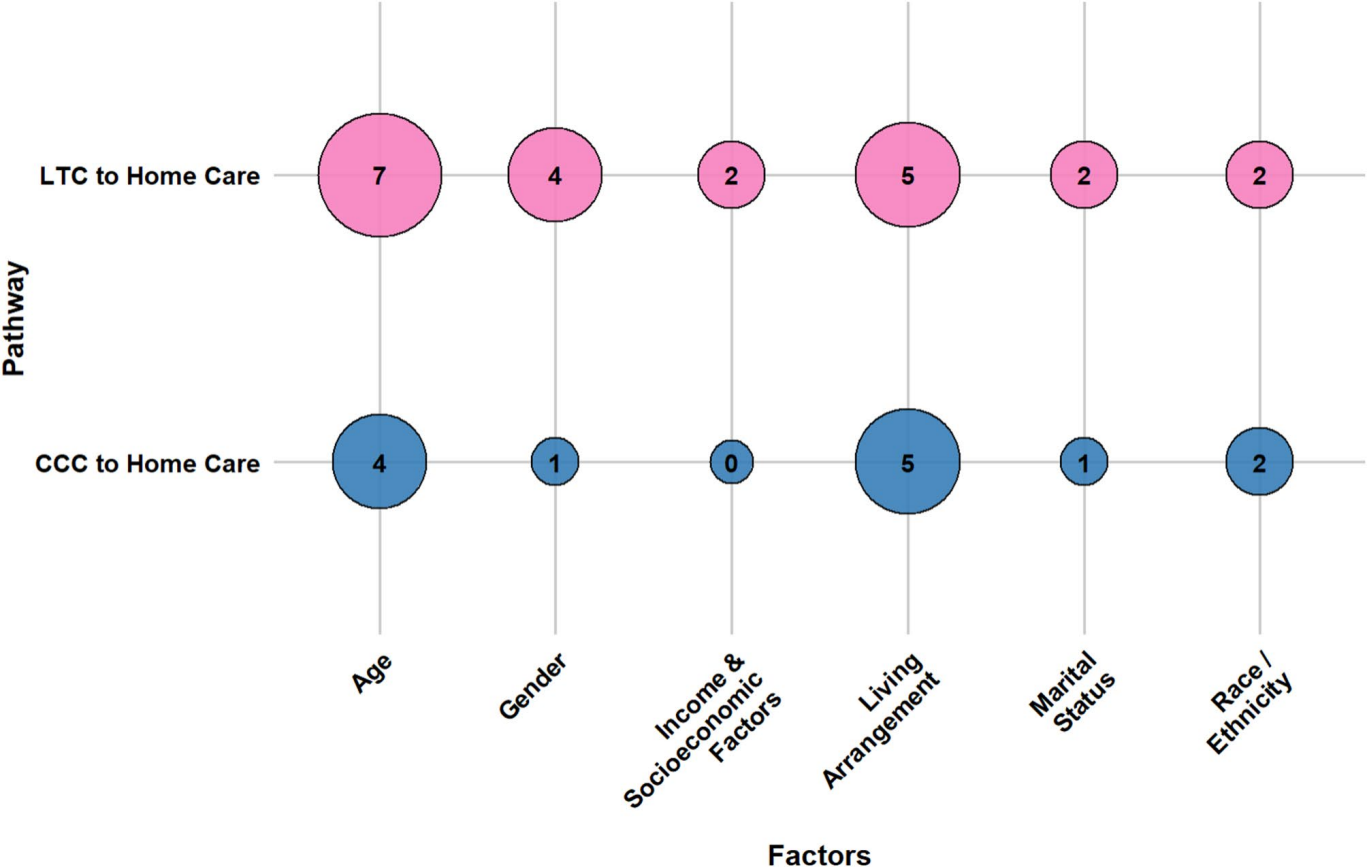
The influence of socio-demographic determinants on community reintegration transitions

**Fig. 8:**
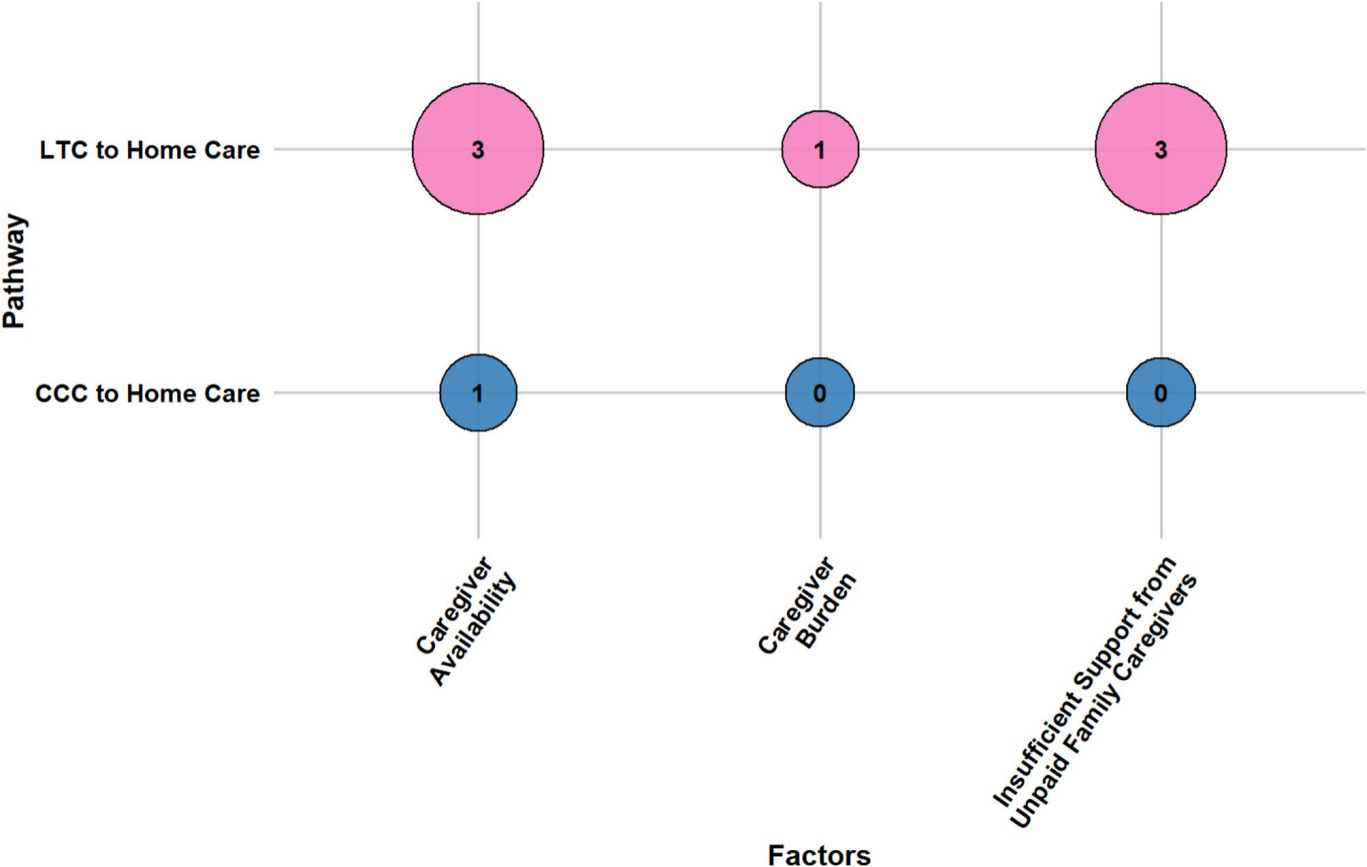
The influence of caregiver support determinants on community reintegration transitions

**Fig. 9:**
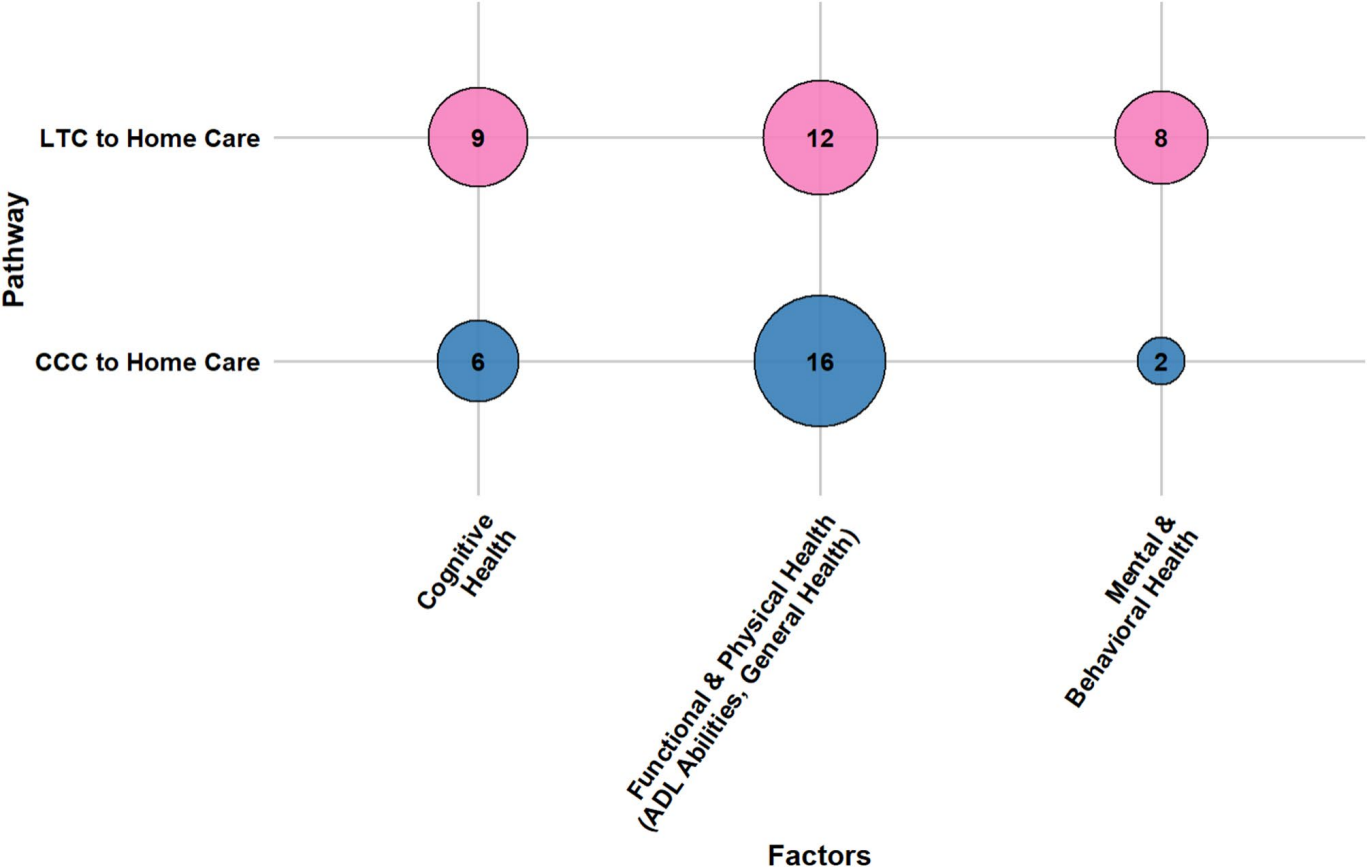
The influence of health condition determinants on community reintegration transitions

**Fig. 10:**
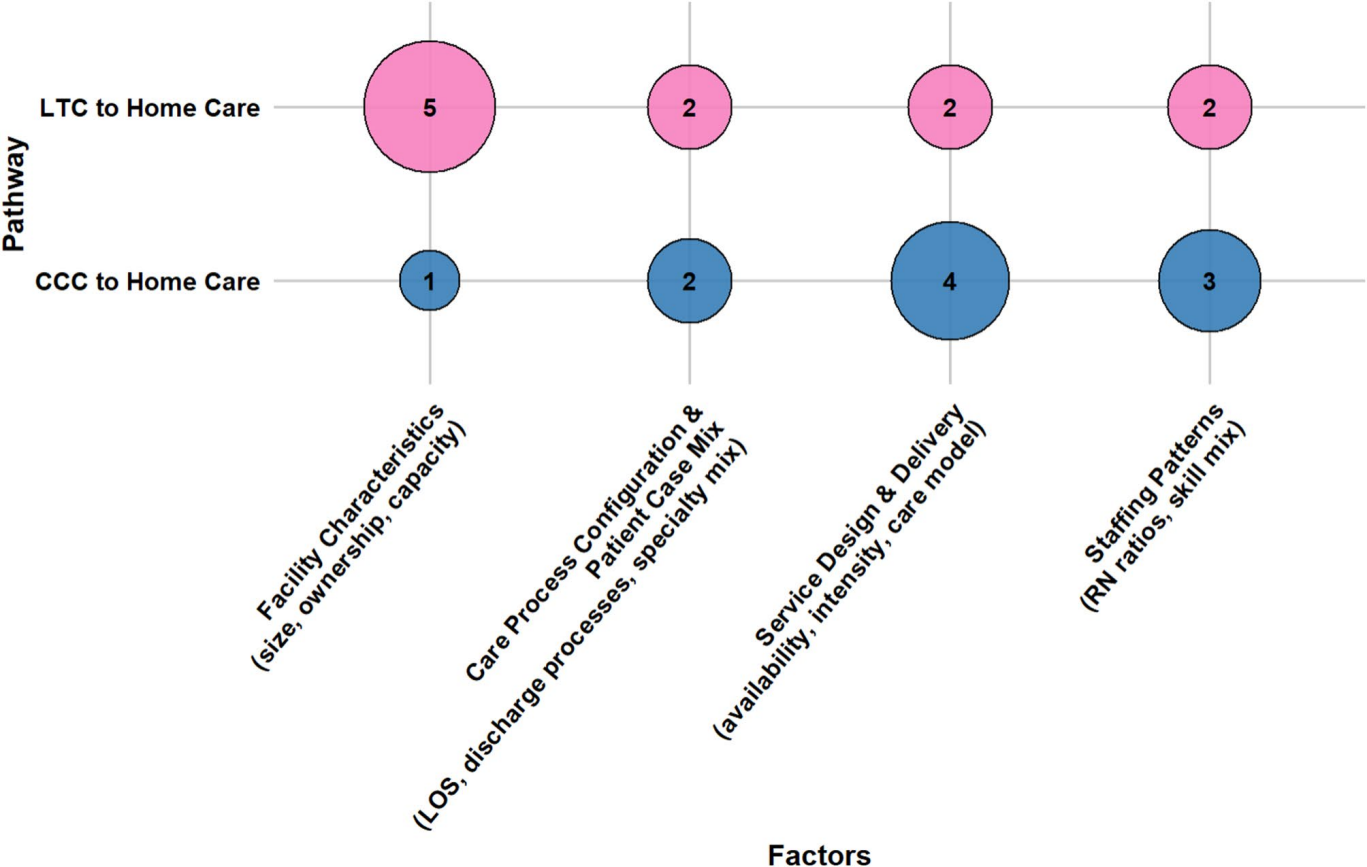
The influence of healthcare system determinants on community reintegration transitions

**Fig. 11:**
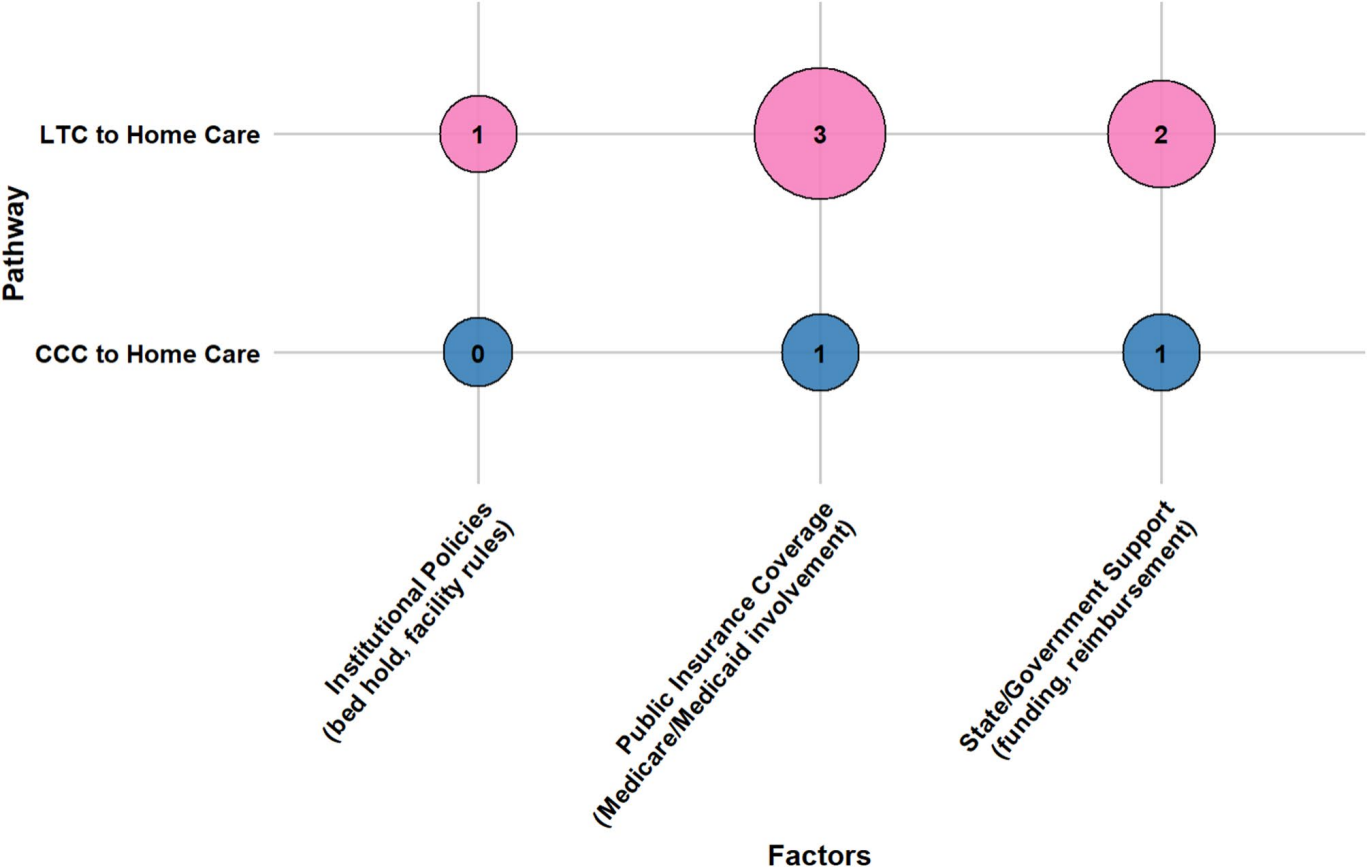
The influence of reimbursement and funding determinants on community reintegration transitions

**Fig. 12:**
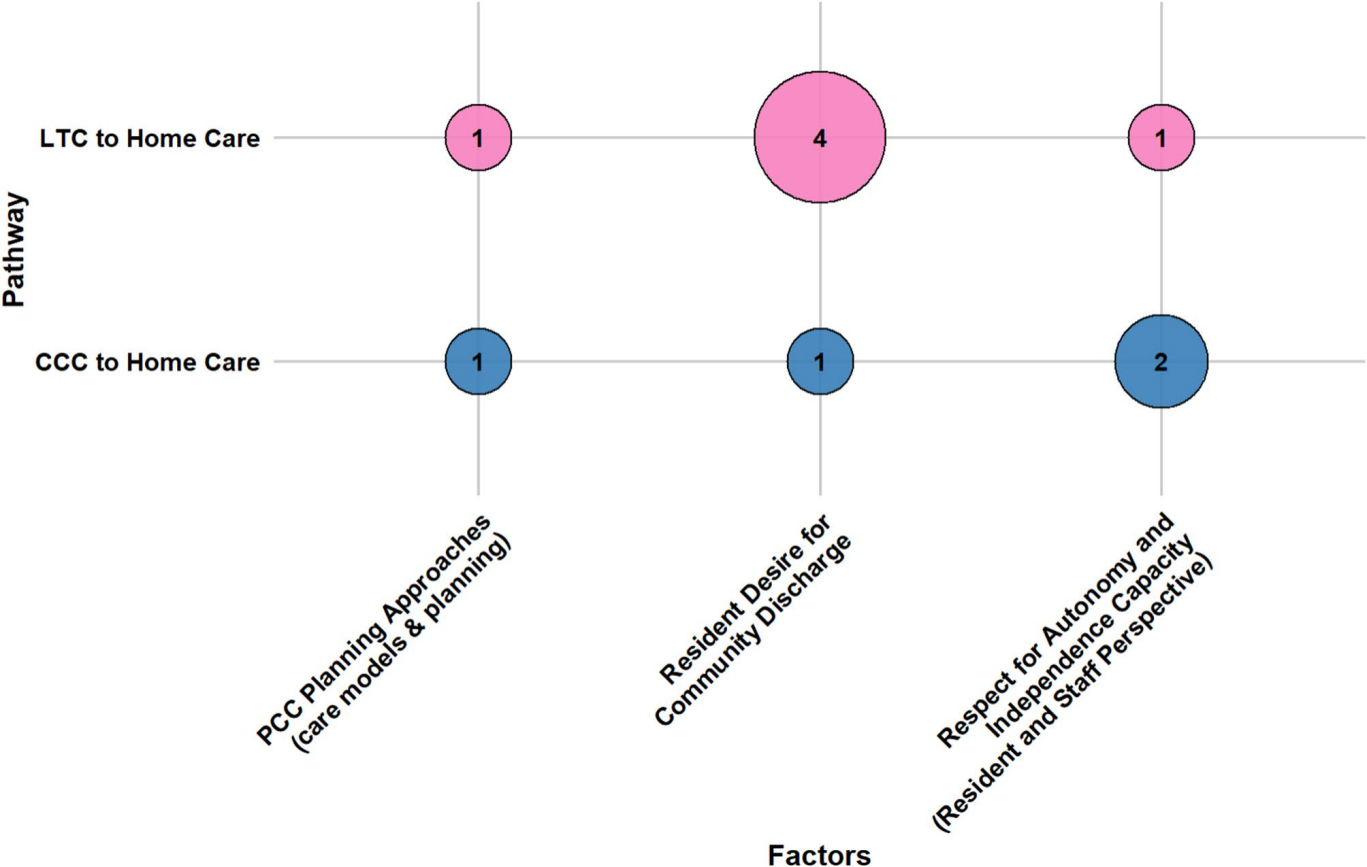
The influence of person-centered care determinants on community reintegration transitions

Older adults’ transitions from LTC and CCC to home care are shaped by a confluence of socio-demographic characteristics, caregiver support, health conditions, healthcare system attributes, reimbursement and funding policies, and person-centered care factors. This section presents a consolidated overview of key determinants across both pathways. Section S5 of the Supplementary Materials presents detailed, study-specific findings with citations, while Table S4 lists all identified factors and their frequency. Figures 7, 8, 9, 10, 11 and 12 offer a broad visual perspective, grouping key elements into overarching categories to complement the results. These bubble charts are compiled from studies with diverse designs, regardless of methodology, and are scaled independently from their PAC counterparts to reflect community reintegration data only.

#### Consolidated overview of key determinants in seniors’ community reintegration transitions

Socio-demographic characteristics consistently influenced community reintegration transitions, with multiple social and structural factors shaping outcomes across both LTC and CCC pathways. Younger age was more often associated with successful transitions, while advanced age frequently acted as a barrier. Gender effects were mixed: female sex more often functioned as a facilitator, particularly in LTC transitions, while one study identified male sex as promoting transitions. Race and ethnicity displayed notable patterns: non-White and Hispanic residents showed higher rates of home discharge, whereas White ethnicity was linked to lower transition rates, especially in CCC contexts.

Living arrangements and household composition also played a decisive role. Individuals living with others or with access to in-home support were more likely to be discharged home, while living alone and experiencing social isolation were common barriers. Marital status followed a similar pattern, with marriage linked to greater reintegration success and single status associated with lower discharge rates. Socioeconomic conditions, including higher family income and stable housing, facilitated transitions, whereas financial hardship and housing instability constrained discharge potential. These socio-demographic influences were more thoroughly documented in LTC than in CCC. Figure 7 illustrates socio-demographic factors affecting transitions.

Caregiver support was a critical enabler, particularly in LTC-to-home transitions. The presence of a committed caregiver, especially one engaged in planning and capable of meeting post-discharge care demands, was consistently associated with successful reintegration. Strong care partner relationships, adequate support resources, and alignment with the older adult’s needs and preferences further contributed to positive outcomes. Conversely, caregiver-related barriers included insufficient family support, unavailability of informal caregivers, and caregiver burnout, particularly when expectations or capacity did not match the intensity of care required. In CCC transitions, caregiver dynamics were less frequently examined but appeared similarly influential; supportive attitudes toward discharge and early caregiver involvement improved outcomes. Figure 8 highlights caregiver support as a critical factor.

Health conditions were central determinants of community reintegration, with consistent evidence that cognitive, emotional, functional, and physical limitations hindered successful discharge from institutional settings. Cognitive impairment, particularly dementia and memory loss, was one of the most frequently cited obstacles across both LTC and CCC pathways. Mental health conditions such as depression, anxiety, and broader psychiatric disabilities also emerged as significant barriers, with depression receiving particular emphasis in both settings. Behavioral challenges, including agitation and aggression, were additional impediments, especially among LTC residents.

Physical health status played a critical role. Better overall health and medical stability facilitated transitions, whereas higher comorbidity burdens and complex health needs reduced discharge likelihood. Functional status was among the most consistently reported factors: reduced ADL performance, high care needs, and general functional dependence limited reintegration, while higher functional independence and mobility facilitated it. Notably, one study highlighted that older adults’ own beliefs about their ability to regain ADL independence positively influenced their transition outcomes. Together, these findings underscore the strong link between cognitive, emotional, physical, and functional capacity and the likelihood of a successful return home. Figure 9 presents health conditions influencing transitions.

Healthcare system factors shaped outcomes through multiple interrelated pathways, including facility characteristics, staffing models, operational structures, and therapy intensity. In LTC settings, higher registered nurses (RN) staffing levels, favorable RN-to-nurse ratios, and greater staffing per bed were associated with improved discharge outcomes. Facility size and ownership showed mixed effects: some evidence supported transitions from public or larger facilities, while other findings suggested barriers.

In CCC settings, hospital-based and nonprofit facilities, dedicated rehabilitation units, structured care models (e.g., the Siebens Domain Management Model), and high-volume specialty programs were more likely to support transitions. Moreover, lower bed‑to‑nurse ratios, higher RN staffing levels, and balanced skill mixes facilitated integrations, whereas high ratios of licensed practical nurses to RN posed barriers. Therapy-related variables, including higher-intensity rehabilitation and condition-specific programming, were associated with increased discharge rates. Lastly, across both settings, inefficient discharge practices, unclear care pathways, and limited service availability impeded transitions, highlighting the importance of institutional readiness. Figure 10 represents the influence of healthcare system factors on transitions.

Reimbursement and funding policies influenced reintegration through the design and adequacy of public financing structures. In LTC-to-home transitions, Medicaid and Medicare policy features played a prominent role. Greater spending on home care, more generous reimbursement rates, and the absence of financial incentives to retain residents (e.g., bed-hold policies) were linked to higher discharge rates. Broader public investment in home care infrastructure was also associated with stronger reintegration performance.

In CCC transitions, the evidence was more limited but echoed similar dynamics. Medicaid home care coverage facilitated transitions, while Medicaid enrollment in itself was associated with reduced discharge likelihood in some contexts, potentially reflecting coverage gaps or system-level inefficiencies. These findings point to the importance of aligning reimbursement models with policy goals that support home and community-based care. Figure 11 outlines the influence of reimbursement and funding policies.

Lastly, person-centered care was as a key enabling domain, particularly when individual preferences, values, and perceptions of recovery potential were integrated into discharge planning. In both LTC and CCC settings, older adults who expressed a desire to return home and had a support person with positive attitudes toward discharge were more likely to transition successfully. Supportive care planning processes that incorporated patient goals, preferences, and self-efficacy were likewise positively associated with outcomes.

By contrast, transitions were hindered when staff or residents held negative beliefs about the feasibility of returning home, or when care planning failed to reflect individual autonomy. Structured care models that emphasized shared decision-making and person-directed goal setting were linked to improved reintegration. These findings reinforce the importance of psychological readiness, patient-centered models, and preference-sensitive planning in enabling successful transitions from institutional care to home. Figure 12 summarizes the influence of PCC factors.

## Discussion and policy implications

This scoping review, based on a comprehensive analysis of 120 publications, sought to elucidate the factors influencing older adults’ transitions across two critical pathways: PAC transitions and community reintegration transitions. While the results section presented the raw findings, this section delves deeper into the interpretations, reasoning, and implications provided by the selected studies. We also discuss how these determinants have been integrated into care models and policies, alongside recommendations for research and practice.

Guided by the high-level themes identified in the results, our synthesis highlights the most significant determinants in both pathways. Additionally, we provide an overview of how studies have acknowledged their limitations and proposed directions for future research within each pathway. Overall, our review suggests that policymakers reform policies to integrate these key determinants into care models, thereby enabling the development of individualized care placement plans for older adults.

### PAC transitions

#### Socio-demographic characteristics

**Age.** Advancing age is a significant determinant of discharge to institutional care rather than returning home post-hospitalization. Across diverse countries and medical conditions, studies consistently show that older adults are more likely to transition to LTC or CCC, even after adjusting for clinical factors [50–54]. This pattern is largely attributed to age-related physical and cognitive declines, increased frailty, greater dependency, and reduced functional capacity, all of which complicate recovery and necessitate structured post-discharge care [55–58].

While age is widely acknowledged as a key factor in transitions, few studies propose targeted, age-specific strategies. Existing recommendations include expanding institutional care capacity to meet growing demand [50] and strengthening home medical care services for highly dependent older adults [56]. However, there remains limited focus on interventions designed to support older adults in returning home. The growing evidence base reinforces the need for transitional programs tailored to age-related challenges, aimed at bolstering functional capacity and enabling more older adults to return home safely.

**Gender.** Studies predominantly indicate that females are more likely to transition to LTC and CCC after acute hospitalization [50, 52, 55, 59, 60]. This trend is observed across various medical conditions and procedures, including cardiac surgery, TKA, and TAVR [52, 58, 59]. However, this association is not uniform across all contexts. For example, a study on traumatic brain injury (TBI) revealed that females were more likely than males to achieve community residence [54]. These findings suggest that the influence of gender on transitions may depend on specific health conditions and care settings, warranting further investigation.

The higher likelihood of institutional discharge among females is often attributed to social and caregiving factors. Females are more likely to live alone or lack informal caregiving support from a spouse or family member, making institutional care more necessary [50, 61–63]. Additionally, their longer life expectancy increases the likelihood of experiencing dementia, which often requires higher levels of care [60, 61]. Accordingly, there is a clear need for gender-sensitive discharge planning and resource allocation [52, 62]. Targeted interventions for females, particularly those living alone or without family support, may improve their ability to return home safely after hospitalization. This may involve enhancing home care services and creating robust post-discharge support systems tailored to older females [52, 61, 62]. Furthermore, integrating gender considerations into predictive models could improve the identification of older adults at risk of PAC institutionalization [62, 63]. At the same time, emerging evidence that females are more likely than males to be discharged to home care in some cohorts reminds us that sex‑specific patterns may invert in less intensive settings.

**Marital status.** Studies consistently show that unmarried older adults are more likely than married ones to be institutionalized after hospitalization. The presence of a spouse provides essential emotional and physical support, which boosts functional independence and enables older adults to remain at home [64, 65]. In contrast, unmarried individuals often lack immediate support systems, making them more vulnerable to institutionalization. Notably, marriage provides additional protection against LTC transitions for non-Hispanic whites and Hispanics but not for non-Hispanic Blacks, for whom kin networks may serve as an effective substitute [66]. Overall, these findings shed light on the importance of considering marital status in discharge planning [62, 65]. Although this determinant is not modifiable, it can signal the need for targeted interventions, such as homecare packages, social engagement programs, or emergency assistance, to help single older adults safely return home [65].

**Race.** Hispanic older adults are more likely than non-Hispanic whites to transition to home care, even when accounting for health and socioeconomic factors [54, 66]. However, the findings are inconsistent for Black older adults: some studies show higher institutional discharge rates [58, 67], while others report lower odds [53]. For Asian older adults, patterns also vary; one study noted the highest rate of institutional discharge for them after knee arthroplasty [58], while another suggests higher community residence rates among Asian/Pacific Islander individuals [54]. These diverse associations can be understood through cultural preferences, family support, and systemic barriers. For example, Hispanic older adults often benefit from strong family support for informal caregiving, while higher readmission rates among Black individuals may reflect unmet medical needs or unequal access to quality PAC [54, 66]. More specifically, structural factors, including discrimination, the underrepresentation of diverse healthcare professionals, gaps in health education, and limited culturally competent services, can restrict minority access to suitable care [61, 66, 67].

The implications of these insights are critical for healthcare policy, emphasizing the need for culturally tailored discharge planning, proper follow-up care, and efforts to address systemic healthcare inequities. These measures are essential to meet the unique challenges of aging racial and ethnic minorities, such as historical discrimination, shifting family dynamics, and limited access to appropriate PAC [54, 66, 67].

**Socioeconomic status.** Studies from North America and Nordic countries show that, in urban settings, lower socioeconomic status often restricts older adults’ access to home-based recovery options, leaving institutional care as the only alternative [64, 67, 68]. Notably, healthcare professionals report that low-income older adults require more intensive care coordination due to difficulties navigating the healthcare system and accessing their rights [68]. On the other hand, low-income older adults in rural U.S. areas struggle to access institutional care due to low Medicaid reimbursement rates, reluctance of facilities to accept Medicaid patients, limited availability of LTC staff and beds, transportation barriers, and bureaucratic delays in care coordination [69].

To address these socioeconomic disparities, tailored policy solutions are needed. In urban areas, preoperative counseling and resource planning programs for low- and middle-income older adults are recommended [64, 67]. Furthermore, insights from Nordic countries suggest that simplifying reimbursement procedures and providing additional support for healthcare navigation could enhance access for low-income older adults [68]. In rural regions, reforms such as revising Medicare’s three-night stay rule, increasing Medicaid reimbursement rates, expanding the rural LTC workforce, and improving transportation services could improve institutional care access [69].

#### Caregiver support

Caregivers frequently report feeling unprepared for post-acute transitions, which contributes to heightened stress and a sense of overwhelm that impedes their ability to provide effective support [70]. This lack of preparedness often delays the initiation of home care, which is commonly accessed only after hospital readmission or a severe health decline, by which time caregivers are already burnt out [71]. Furthermore, unprepared caregivers may experience moral and emotional strain, potentially compromising the quality of home care and increasing the risk of readmission [72, 73]. Overall, when caregivers lack the capacity to meet care demands, institutionalization often becomes inevitable [74].

These findings underscore the value of investing in caregiver support to delay LTC admissions and promote successful aging at home [20, 75]. Effective discharge planning should involve a thorough assessment of caregiver capacity to alleviate their emotional strain [72, 74]. Targeted interventions to support caregivers, such as training programs and active involvement of healthcare professionals. can further enhance transition processes and reduce readmissions [73, 74].

#### Health conditions

**Specific medical conditions.** Multiple studies have identified various medical conditions as determinants of discharge destinations, reflecting the complex care needs these conditions impose. For example, Parkinson’s disease is a notable risk factor for LTC admission, particularly in younger seniors, as it severely impairs independence [76]. Similarly, conditions like incontinence contribute to functional decline and caregiver burden, complicating home discharge [55, 65, 77]. Additionally, various conditions such as TBI, metastatic cancer, pressure ulcers, chronic liver disease, and dysphagia are linked to institutionalization due to high care complexity and decreased functionality [54, 59, 77, 78].

These findings highlight the need for tailored interventions and proactive care planning for older adults with complex conditions. For example, early management of incontinence and dysphagia can improve functional outcomes and support independence, potentially reducing institutionalization [77]. For more complex cases, like metastatic cancer or TBI, comprehensive discharge planning and care coordination are essential for enabling home transitions [54, 78].

**Cognitive status.** Older adults with dementia or cognitive impairments are at a higher risk of LTC placement, whether following general hospitalization or specific procedures like lower-extremity surgery [55, 57, 60, 62, 64, 79, 80]. This risk increases when dementia coexists with conditions like hip fractures, cardiovascular disease, or aspiration pneumonia [55, 78, 79]. Cognitive impairments complicate care management by limiting communication, self-management, learning capacities, and discharge coordination [57, 61, 65, 70]. Additionally, behavioral and psychological symptoms of dementia (BPSD) exacerbate caregiver burden, further increasing the likelihood of LTC placement [65, 70, 81].

The influence of dementia on transitions to CCC presents a more ambiguous picture [57, 64]. One study indicates that older adults without dementia are more likely to transition to CCC, where the focus is on functional recovery, while those with dementia typically transition to LTC, which prioritizes ongoing supportive care. However, another study links dementia to an increased likelihood of transitions to both LTC and CCC, reflecting its strong association with frailty and heightened care needs [64]. This apparent paradox underscores the need for further research to elucidate dementia’s role in CCC transitions and to develop tailored rehabilitation programs that optimize functional outcomes for older adults with dementia, as highlighted by the European Consensus Group [57].

Several strategies have been suggested to improve transition outcomes for cognitively impaired older adults. Hospitals should prioritize early identification of cognitive impairments via tools such as the Rowland Universal Dementia Assessment Scale (RUDAS) and the Dementia Assessment Sheet for Community-based Integrated Care (DASC-8), enabling timely interventions to support home care transitions [82, 83]. Specialized interventions, such as cognitive rehabilitation and targeted support programs, can further enhance recovery and increase the likelihood of returning home [57, 65]. Moreover, strengthening in-home services, including physiotherapy and caregiver training, helps individuals with moderate-to-severe dementia maintain independence [61, 81]. Finally, expanding performance-based care models, such as the quality and outcomes framework (QOF), which incorporates dementia reviews assessing both the elderly and caregiver needs, can reduce institutionalization risks by improving care coordination and support [55].

**Functional status.** Impaired mobility and ADL dependencies greatly increase the likelihood of institutional placement, while functional independence supports home transitions [60, 83–86]. Functional impairments usually restrict self-care and safe navigation, necessitating the additional support found in institutional settings [53, 56, 60, 84]. Moreover, mobility limitations raise susceptibility to medical complications, heightening the risks of independent living [51]. Notably, older adults with limited mobility prior to hospitalization are more likely to need institutional PAC, underscoring the importance of baseline function in recovery [57]. It is also worth mentioning that functional limitations sometimes combine with cognitive impairments, further complicating care needs and transitions [61].

These findings highlight the need for early and thorough functional assessments in acute care to guide discharge planning [51, 60]. Standardized assessments, such as the De Morton Mobility Index (DEMMI), AlphaFIM, Activity Measure for PAC (AM-PAC), or Barthel Index, can identify individuals at risk of institutionalization, enabling targeted interventions [51, 83, 85]. Additionally, rehabilitation programs focused on improving mobility and ADL independence during hospitalization can increase home discharge rates [53, 56, 57]. For older adults with dementia, integrated care models that address both functional and cognitive challenges may improve care continuity and transition outcomes [61].

**Mental health conditions.** Depression is a strong determinant of institutional placement for individuals aged 60–69, though its influence diminishes in older cohorts [76]. Behavioral symptoms, such as aggression and severe anxiety, also drive institutionalization across all age groups, particularly among dementia patients [73, 84, 87, 88]. Depression and anxiety often impair self-care abilities, reducing the chances of community reintegration [73]. Aggressive behaviors raise safety concerns, prompting healthcare professionals to prefer institutional over home placements [87, 88]. Care transitions can also exacerbate psychological distress, especially in dementia patients, creating a cycle where deteriorating mental health further narrows discharge options [61]. This burden also significantly affects caregivers, whose own mental well-being influences the feasibility of home care [61, 84].

These findings point out several priorities for clinical practice and policy development. Comprehensive mental health screening should be integrated into transition planning, with age-specific strategies to mitigate depression-related institutionalization for younger seniors [73, 76, 87, 89]. Additionally, resilience-building interventions should be implemented to empower older adults to manage depression and adapt more effectively to care transitions [73]. Equally important, transition support must address the psychological needs of both older adults and their caregivers, recognizing the interdependence of these groups in shaping care outcomes [61, 84]. Overall, policies should promote integrated, continuous care models that provide medical, psychological, and social support throughout the transition process [73, 88].

#### Healthcare systems

**Clinical interventions.** Several studies indicate that older adults undergoing major surgeries, such as TKA or hip fracture repairs with intramedullary implants, are at a higher risk of institutional discharge due to the need for intensive postoperative care [57, 67]. Hospital treatments like electrocardiography and respiratory therapy are also linked to increased odds of institutional discharge, indicating more extensive care needs [56]. In contrast, pre-hospitalization in-home physiotherapy has been shown to enhance older adults’ functional status, facilitating home discharge [81].

These findings point to the importance of early discharge planning for older adults likely to need institutional care post-intervention. Tools like aspiration pneumonia prediction models and the ADELES risk score (Age, Dementia, Eating Dependency, Leg Surgery, and Serum Albumin) for lower-extremity surgeries can support tailored discharge planning and enhance preparedness for the elderly and their families [64, 78]. Identifying modifiable risk factors, such as the type of anesthesia used and the duration of preoperative hospitalization, also creates opportunities for interventions to reduce the likelihood of institutional discharge [64]. Additionally, preoperative counseling that addresses functional outcomes and potential discharge options can improve shared decision-making and align patient expectations [52, 64].

**Hospital Operational Efficiency.** Hospital operational efficiency impacts care transitions through factors such as length of stay, staffing levels, bed capacity, financial incentives, and interprofessional communication. Shorter hospital stays may indicate inadequate care coordination and limited discharge planning, which complicate transitions for older adults, particularly stroke survivors [74, 90]. To be more precise, for complex conditions like stroke, premature discharge can hinder recovery, increase readmissions, and impose an immense economic burden on healthcare systems [74]. Staffing levels and bed capacity also play critical roles. For example, in Japan, hospitals with high patient-to-nurse ratios and larger bed capacities are more likely to discharge older adults to institutional settings [56]. This trend is partly driven by the Diagnosis Procedure Combination (DPC) payment system, which incentivizes rapid discharges to increase throughput and reduce costs; however, it may inadvertently compromise individuals’ readiness for home care [56]. Communication barriers among healthcare professionals further disrupt transitions [73]. For instance, in Sweden and Denmark, issues like poor inter-professional communication, unclear responsibilities, and limited access to primary care providers are common [90, 91]. In rural U.S. areas, delayed communication between hospitals and LTCs extends patient stays [69]. It is also worth noting that power imbalances between providers and patients can limit seniors’ and family engagement in transition planning, potentially misaligning care with older adults’ preferences [71, 72, 92, 93]. 

The policy and practice implications are multifaceted. Firstly, improving communication and information transfer through standardized handover procedures and stronger interprofessional collaboration is essential [69, 73, 74, 90, 91]. Staff training programs can also strengthen care coordination skills and improve understanding of the transition process [90, 93]. Organizational reforms, such as clarifying professionals’ roles, establishing guidelines, and adjusting financial incentives to prioritize patient-centered outcomes, are also necessary [56, 91, 93]. Lastly, actively involving the elderly and their families in transition planning ensures that decisions align with their preferences [71, 72, 92, 93].

**Health information technology**. Effective information sharing between care settings reduces miscommunication, enhances continuity of care, and minimizes medical errors during transitions [73, 91, 94]. For instance, fragmented hospital readmissions have been linked to higher discharges to CCC and lower odds of returning home. However, when admission and readmission hospitals shared a health information exchange (HIE), the chances of discharging home increased [95]. This demonstrates how HIE can counteract the negative effects of fragmented care by improving the information flow between providers. Moreover, the COVID-19 pandemic accelerated the adoption of telehealth and remote monitoring, significantly transforming healthcare delivery. Telehealth use rose 38-fold during the pandemic, allowing providers to monitor older adults remotely and receive real-time alerts about potential issues [96]. These technologies enhance care quality by preventing avoidable health complications, enabling more individuals to receive care in the comfort of their own homes.

Together, the accelerated adoption of EHR, HIE, telehealth, and remote monitoring signifies a lasting shift in healthcare delivery models, emphasizing the growing importance of technology in care practices [74, 91, 96]. The implications for policy and practice are clear: investing in health information technology infrastructure can considerably improve care transitions. Thus, strategic efforts should prioritize enhancing communication systems and data-sharing platforms to minimize adverse events and improve transitions [73, 74, 90, 91, 94].

**Access to services.** Limited service availability significantly restricts transition options for older adults, often resulting in prolonged hospital stays or premature LTC placements. A scoping review across North America, Europe, and Australia identifies home care shortages as a key barrier in post-acute transitions [92]. Geographic disparities, particularly in rural areas, compound these issues with challenges like limited LTC infrastructure, transportation barriers, and workforce shortages [69, 75]. In Nordic countries, local variations in healthcare resources, such as access to post-discharge physician home visits, further contribute to unequal PAC access [68]. Workforce shortages are also a critical factor, with Canada having one of the lowest LTC worker-to-resident ratios among the OECD countries, limiting both home care and institutional services [97]. In the U.S., staffing shortages and reluctance to serve rural areas exacerbate the lack of home care, often resulting in institutional placements [75].

Accordingly, policy implications include increased investment in home care, targeted workforce recruitment in underserved areas, enhanced rural transportation infrastructure, and the development of adaptable housing options [71, 75, 97]. Additionally, integrated care roles, like discharge planning nurses and home care coordinators, are vital for service continuity and advocacy during transitions [71].

#### Literature gaps and future research

The studies highlight key limitations and suggest future research directions. Research on socio-demographic factors is largely quantitative, regionally concentrated (North America and Europe), and often lacks qualitative insights into broader cultural and personal factors [60, 61]. Methodological challenges such as missing data and limited variables further restrict the depth of analysis [55, 58, 63]. Furthermore, small sample sizes for minorities and reliance on retrospective data limit generalizability, with unmeasured variables potentially overlooking older adults’ preferences [54, 58, 66]. Thus, broader geographic representation and qualitative methods are needed. Caregiver support studies, predominantly qualitative, offer in-depth insights but are limited by small, geographically concentrated samples and retrospective designs [71, 75, 88]. Future research should use larger, more diverse samples and mixed method approaches to enhance the evidence base.

Regarding health conditions, research is largely quantitative and spans North America, Europe, Asia, and Australia, highlighting the global importance of these conditions in transitions [76, 83, 85, 98]. However, inconsistent definitions and varying measurement tools hinder cross-study comparability [38, 60]. Notably, some studies overlook the role of social and environmental factors, which are critical to understanding the influence of health conditions on transitions [65]. Furthermore, many resources focus only on immediate outcomes, neglecting long-term recovery trajectories and the effectiveness of post-transition interventions [57, 65]. Future research should utilize standardized measures, adopt longitudinal designs, incorporate social and environmental factors, and explore integrated interventions that address the complex needs of older adults.

Finally, research on healthcare system factors is broad but constrained by small sample sizes and limited input from diverse stakeholders [68, 73, 90, 91]. Specifically, studies often prioritize healthcare professionals’ perspectives, particularly nurses, while excluding the experiences of older adults, families, and other key stakeholders involved in transitions [68, 73, 91]. Hence, future research should employ larger sample sizes and incorporate a broader range of stakeholder perspectives to enhance the understanding of how these factors influence care transitions.

### Community reintegration transitions

#### Socio-demographic characteristics

**Age.** Older age reduces the likelihood of transitioning from LTC and CCC to home care [99–104]. This is attributed to medical complexity, functional impairments, cognitive decline, and inadequate social or family support [100, 102, 105]. To improve transitions to home care, targeted interventions and policy reforms are needed. Firstly, early discharge planning within the first 90 days of institutionalization can facilitate transitions [106]. Moreover, tailored support systems, such as comprehensive home care, cognitive and physical rehabilitation, and enhanced family support, may address critical barriers [100, 102, 105]. Additionally, pre-admission assessments to identify residents with potential for community discharge and resource prioritization for facilities with higher success rates could promote transitions [103, 104].

**Gender.** Most studies indicate that female residents are more likely than male residents to transition from LTC and CCC back to the community [6, 99–101]. This trend is commonly attributed to the stronger informal support networks among females, including family ties and assistance from children and relatives [99]. In contrast, males typically depend on spousal support and may face challenges if their spouse cannot provide care, resulting in lower discharge rates [99].

Interestingly, findings from a Japanese study present a contrasting perspective: male residents were more likely than females to be discharged home, likely due to caregiving patterns in which aging wives often outlive their husbands and act as caregivers [102]. This suggests that, in certain cultural contexts, males may have greater access to caregiving, enhancing their likelihood of returning home.

These gender-specific differences emphasize the need for tailored discharge planning. Policymakers should recognize the barriers that male residents may face due to smaller informal support networks and consider strategies to enhance their access to community resources [6, 99]. For example, identifying and involving friends or extended family members in post-transition care could be pivotal [99]. For female residents, policies should focus on sustaining and strengthening family support networks to facilitate their successful reintegration into the community [99, 101].

**Marital status.** Research consistently indicates that being married increases the likelihood of discharge to home, as spouses often provide essential caregiving and emotional support, facilitating a smoother transition. In contrast, unmarried individuals tend to rely more on institutional care [101, 105, 107]. As mentioned earlier, these findings highlight the need to consider marital status in discharge planning and to design targeted interventions for unmarried older adults. Additional support, such as community-based programs, could address the unique needs of unmarried individuals, helping them transition back to the community [101, 105, 107].

**Race and ethnicity.** Non-white older adults, including Hispanic, Asian, and Black individuals, are more likely to transition back to the community compared to their white counterparts, possibly due to stronger family caregiving norms and reciprocal support networks [99, 106–108]. However, the relationship between race and transitions is complex and context-dependent. For instance, while Hispanic older adults generally show higher transition rates, those with mental health disabilities face significant discharge barriers, likely because of cultural stigma around mental illness [106]. Additionally, although Hispanic and Asian older adults with TBI tend to have higher odds of successful discharge, the underlying mechanisms are not well-understood [108]. These findings highlight the necessity for culturally sensitive policies that address the unique needs and barriers experienced by various racial and ethnic groups [99, 106].

**Socioeconomic status.** Research from the U.S. shows that LTCs in areas with higher median family income achieve better discharge rates to community settings, likely due to better access to home care services and support infrastructure [109]. However, housing challenges remain a barrier to community reintegration, especially for individuals who are reliant on Medicaid. In fact, affordability and accessibility issues in housing contribute to transition delays, averaging an additional 86 days [106]. These findings suggest a need for healthcare systems to expand affordable and accessible housing options for older adults transitioning from institutional care to home care [106]. Early identification and proactive intervention in addressing housing needs may help mitigate these transition delays.

#### Caregiver support

Family and caregiver support plays a pivotal role in transitions. For example, Minnesota’s Return to Community Initiative (RTCI) successfully assisted over 4,300 older adults in returning home, primarily through the support of adult children and spouses [110]. In Canada, positive caregiver attitudes toward discharge were associated with shorter CCC hospital stays, underscoring the importance of caregiver involvement in discharge planning [6]. However, caregiver burdens, including stress and unmet needs, can hinder successful transitions. In Manitoba, family caregivers cited overwhelming home care demands and unsustainable caregiving responsibilities as primary factors in choosing LTC [111].

During the COVID-19 pandemic, family-driven transitions in Ontario highlighted how unprepared families struggled with the complexities of home care, often underestimating the required level of care and lacking sufficient support [112]. These findings suggest that while caregiver involvement is crucial, high caregiver burdens and limited support may impede effective transitions. Without adequate resources, formal support, and professional guidance, caregivers may feel unprepared and struggle to manage older adults’ complex needs [37, 101, 112].

Policymakers are encouraged to strengthen caregiver support through training, respite care, community services, and transportation assistance, which can help reduce caregiver burden and stress while improving transition outcomes [101, 106, 110, 111, 113]. Additionally, engaging caregivers early in discharge planning ensures that they are better prepared and supported, ultimately contributing to more successful transitions [6].

#### Health conditions

**Cognitive status**. Cognitive status strongly influences care transitions, with evidence suggesting a dose-response relationship: Severe cognitive impairment greatly reduces the probability of discharge to home, while moderate impairment has a less pronounced effect [100, 103, 108, 114]. Gender differences further complicate these patterns, with cognitively impaired male residents, particularly those with extended stays, experiencing notably low discharge rates [99]. In other words, while memory problems decrease the likelihood of discharge for both genders, the effect is more pronounced in males. As mentioned earlier, cognitive decline complicates discharge pathways by impairing medication adherence, nutrition, safety awareness, and care coordination [101, 106, 111]. Families often view LTC as a last resort when cognitive impairments lead to unmanageable safety crises at home [111]. Even well-resourced facilities struggle to discharge severely impaired residents, especially when social support is limited [103, 111, 114].

These findings underscore the importance of early cognitive assessments and targeted interventions to improve the likelihood of home care transitions for cognitively impaired residents. Healthcare providers should incorporate standardized cognitive evaluations, such as the MMSE or the Montreal Cognitive Assessment (MoCA), into both admission and discharge planning processes [103, 115]. Early identification enables the creation of personalized care plans that address cognitive deficits and optimize rehabilitation. Tailored rehabilitation programs focusing on memory, attention, and problem-solving skills can enhance cognitive function, improve the ability to manage ADLs, and increase the likelihood of home discharge [107, 115]. Policymakers should also mitigate the challenges for males with weaker social support systems by implementing gender-sensitive care models and strengthening external support networks [99]. Lastly, specialized discharge planning that addresses safety and supports families and caregivers is essential [105, 111].

**Functional status**. Functional capacity, particularly ADLs, strongly influences transition success [103, 115–118]. While Buttke et al. found that residents with moderate functional dependence had the highest transition potential, other studies generally demonstrate that higher functional independence is associated with higher rates of community reintegration [100, 103, 110, 116–118]. This pattern holds across different assessment tools, including the Barthel Index in Australia [116, 118], ADL indices in the United States [100, 103], and the Timed Up and Go (TUG) test in Europe [113].

As mentioned earlier, functional and mobility impairments can limit self-care and safe navigation, hindering independent living [6, 113, 114]. Cognitive deficits further reduce the likelihood of successful transitions, compounding the challenges posed by physical impairments [103, 107, 108]. Notably, the interaction between functional status and individual beliefs about recovery potential also affects transitions; self-efficacy and positive expectations might drive older adults to engage more actively in their rehabilitation and recovery, thereby facilitating their discharge [119]. Additionally, facility characteristics moderate these outcomes, with resource-rich environments achieving higher transition rates due to the availability of tailored rehabilitation programs and support services [100, 114].

These findings underscore key policy and practice implications. Early, comprehensive functional assessments using tools like the Barthel Index, FIM scores, and TUG test are essential to guide transition planning [103, 115, 116]. Assessments by physical and occupational therapists provide reliable transition predictions due to their close involvement with residents’ functional impairments and rehabilitation progress [103, 120]. Thus, incorporating these assessments into the discharge planning could help identify older adults at higher risk of failed transitions and target interventions to improve their outcomes. Specifically, targeted rehabilitation for these residents with functional impairments is strongly recommended to improve their potential for discharge [100, 117]. Policies should also prioritize specialized systems that address both physical and psychological factors, focusing on fostering older adults’ self-efficacy [120, 121].

**Mental health conditions**. Research points to depression, anxiety, and behavioral problems as the most common barriers to successful community reintegration. For instance, the risk of failed transitions from CCC is heightened by increased depression levels; individuals with more depressive symptoms show a higher likelihood of being readmitted to LTC, remaining in the hospital for prolonged periods, or passing away within the study periods [107, 114, 120]. These circumstances also affect caregivers, as their stress and mental health are related to transition success [113].

The existing evidence emphasizes that mental health issues impair older adults’ participation in rehabilitation and self-care, which limits their potential functional improvements for community residence. For example, depression generally reduces motivation and limits engagement in therapeutic activities that may positively influence rehabilitation outcomes [107, 111, 115, 120]. Furthermore, the behavioral problems associated with mental health conditions make it far less likely for an individual to leave institutional care, even when physical health has improved, as concerns about personal safety remain heightened [105, 106, 111].

Overall, these findings show the importance of focusing on integrated mental health support in discharge planning and rehabilitation. Routine mental health screening should be embedded in LTC and CCC admissions and discharge protocols to identify at-risk individuals early [107, 114, 120]. Studies also suggest that targeted mental health interventions (e.g., counseling or psychiatric care), alongside physical rehabilitation, can promote older adults’ engagement and transition success [106, 114, 120]. For individuals with cognitive or behavioral challenges, customized discharge plans that address psychological needs and educate families on care options can further support transitions [105, 111]. It is important to note that strengthening community-based mental health services post-discharge is equally essential, especially for those with severe conditions who may have difficulty accessing these services [100, 106, 113].

#### Healthcare systems

**Facility operational efficiency**. Research on facility size shows mixed results across different contexts. In the U.S., larger facilities with more beds generally achieve higher community discharge rates for short-stay residents, likely because of greater resources, robust care planning, and specialized care. However, this advantage does not extend to long-stay residents [114]. Conversely, in Japan, smaller facilities are associated with higher discharge rates [102]. These facilities may offer more personalized care and higher staffing ratios, which enhances recovery and discharge success, whereas larger facilities may face challenges such as overextended staff. Facilities’ ownership type also affects transitions. Public and nonprofit facilities in both Japan and the U.S. tend to have higher discharge rates, likely because of a stronger focus on patient-centered care and sufficient resource allocation [102, 104]. In contrast, some U.S. studies suggest that for-profit facilities may discharge more long-stay residents, possibly driven by financial incentives to free up beds for new admissions [114].

Staffing levels, particularly among registered nurses (RNs) and rehabilitation staff, are strongly linked to successful home-care transitions. Facilities with higher RN-to-patient ratios and RN-to-total nurse staffing ratios report better discharge outcomes, underscoring the value of skilled nursing [102, 109, 122]. However, beyond a certain threshold, additional increases in staffing may not significantly improve discharge rates, suggesting diminishing returns [104]. The ratio of RNs to LPNs is also impactful; facilities with more RNs relative to LPNs have better outcomes, highlighting the importance of nurse expertise [122]. Facilities with more rehabilitation staff also show a higher likelihood of discharging residents to home, emphasizing the role of therapy services in recovery [102].

Specialization within facilities also enhances discharge success. Facilities with specialized units and higher volumes of specific resident types, such as those recovering from hip fractures, accumulate expertise and tailored care protocols that support successful transitions [104]. Therapy intensity is also critical; providing at least 60 min of therapy per day significantly increases discharge rates, while lower therapy intensity is associated with prolonged institutionalization [123]. Lastly, interdisciplinary care models, like the Siebens Domain Management Model (SDMM), have been shown to improve discharge rates by enhancing collaboration among healthcare professionals and streamlining care processes [124].

These findings represent several implications for policy and practice. Investing in skilled staffing, particularly increasing RN-to-LPN ratios, may improve discharge rates [102, 122]. Similarly, structural adjustments, such as supporting smaller public facilities with robust staffing ratios, are essential in fostering personalized care and boosting discharge success rates [102]. In addition to staffing and structural considerations, promoting specialization within facilities holds promises for improving care efficiency. However, policies must ensure equitable access to these specialized services for all individuals, preventing disparities [104]. In tandem, reimbursement policies should encourage facilities to provide adequate therapy services to enhance recovery and reduce hospital readmissions [123].

Another critical strategy involves adopting interdisciplinary care models, such as the SDMM, to optimize workflows across healthcare teams and enhance discharge planning [124]. Moreover, public reporting on discharge rates and facility performance can empower families to make informed care decisions, aligning older adults’ needs with appropriate resources [104]. Lastly, early targeting of residents for transition programs and improving consumer engagement strategies are essential for promoting successful discharges [106].

#### Reimbursement and funding policies

Studies suggest that increased investment in community-based services promotes successful transitions to home care [37, 109, 125]. In the U.S., limited funding restricts access to these services, forcing many older adults to remain in LTC and CCC despite their preference to return home. In contrast, countries such as Sweden and Norway allocate a larger share of GDP to home care, enabling more older adults to remain in their communities [37].

These findings emphasize that policy reforms should prioritize reallocating resources toward community-based support and adapt reimbursement structures. For example, adjusting Medicaid reimbursement rates in the U.S. could enhance institutions’ capacity to support discharge planning and facilitate community reintegration [108, 109, 125]. Importantly, evidence suggests that expanding access to Medicaid-funded home care services (breadth) is more effective for facilitating successful transitions than increasing per-user spending (intensity), underscoring the need to prioritize service coverage over service intensity [125].

Additionally, policymakers should reevaluate measures like bed-hold provisions, which may unintentionally incentivize facilities to retain residents. While these policies are designed to ensure continuity of care by reserving beds for temporary hospitalized residents, studies reveal that states without bed-hold policies benefit more from increased home care spending [109].

#### Person-centered care

Residents’ desire to return to the community is one of the strongest determinants of successful home care transitions. Older adults who express a preference for returning to the community and hold positive beliefs about their ability to enhance their independence are remarkably more likely to achieve this transition [6, 100, 101, 110, 119]. For instance, in Minnesota’s RTCI, nearly all residents (99%) preferred to return home, emphasizing respecting their choices in transition planning [110]. Similarly, in Ontario’s CCC hospitals, residents who expressed a desire to return home experienced shorter stays [6]. Connecticut’s “Money Follows the Person” (MFP) program further advances this person-centered approach by incorporating input from the elderly, families, and care managers, resulting in smoother transitions and more positive experiences [113]. Notably, positive staff belief in older adults’ capabilities can promote successful transitions, but their influence depends on older adults’ confidence in their own abilities [119].

The implications highlight a need for policy and practice reforms prioritizing person-centered care and supportive networks. Policymakers are encouraged to expand programs such as Minnesota’s RTCI and Connecticut’s MFP to reduce prolonged LTC stays and associated costs while aligning care settings with older adults’ preferences [110, 113]. As mentioned earlier, a strategic shift of resources from institutional to home care may enable more individuals to receive care at home [37]. Moreover, adopting flexible, person-centered practices within LTC facilities, i.e., tailored interventions, training in independent living skills, fostering positive staff attitudes toward residents, and involving older adults in care planning, can further support effective home-care transitions [101, 119].

#### Literature gaps and future research

The included studies highlight several limitations and propose future research directions. Research on socioeconomic factors is primarily U.S. focused and quantitative, often neglecting intersectionality and broader determinants such as education, social networks, and resource access [99–101, 107]. Future work should adopt intersectional approaches using qualitative methods to capture personal experiences and uncover barriers for individuals from different socioeconomic backgrounds. Caregiver support studies, largely from North America, with some representation from Europe and Asia, rely heavily on self-reported data and underrepresent culturally diverse groups [6, 110, 111]. Expanding longitudinal and mixed methods research with diverse populations could provide deeper insights.

Health conditions research spans multiple countries but often uses single-center data, inconsistent measurement tools, and overlooks social determinants and interventions for complex conditions [102, 103]. Longitudinal, mixed methods design with standardized tools are needed to address these gaps. Healthcare system studies, which are mainly U.S.-based, focus on operational efficiency but rely on administrative data lacking resident-level detail and cross-system generalizability [102, 114]. Future research should adopt an international scope and consider unmeasured confounders like care model variations.

Research on reimbursement policies is also U.S.-centric, focusing on Medicaid and excluding broader financial models or caregiver roles [37]. Future studies should examine diverse funding systems and home care’s role in supporting aging in place. Finally, studies on person-centered care are regional and rely on self-reports, limiting their generalizability [100, 112]. Broader, longitudinal research across different regions and cultural contexts is essential to refine person-centered strategies for care transitions.

## Limitations

This scoping review has several limitations that should be considered when interpreting the findings. First, the review was restricted to studies conducted in OECD countries and published in English. While this decision was made to ensure consistency across healthcare systems with comparable demographic trends and policy environments, it limits the generalizability of the findings to non-OECD contexts. Additionally, although many OECD countries publish extensively in English, some relevant studies published in other languages may have been missed due to the language restriction, introducing the possibility of language bias. Second, while we included academic articles, reports, evidence briefs, and commentary pieces, the review may miss some relevant insights by not including other forms of gray literature, such as conference papers. Third, although our focus on the post-2015 literature was intentional in capturing recent developments in healthcare systems, this timeframe may exclude some valuable historical perspectives on care transitions. Finally, while our search strategy was comprehensive, it is acknowledged that no search method is completely exhaustive, and some relevant studies may have been inadvertently omitted.

## Conclusions

This scoping review synthesizes evidence from 120 resources, comprehensively examining the factors influencing older adults’ PAC and community reintegration transitions across LTC, CCC, and home care. The findings emphasize the critical influence of sociodemographic characteristics, caregiver support, health conditions, healthcare system attributes, reimbursement and funding policies, and person-centered care factors on these transitions. Our review’s synthesis of quantitative and qualitative data offers a comprehensive perspective on these determinants. Building on these findings, our study provides a foundation for developing more effective transition planning and decision-making processes. By incorporating the identified factors into care models and transition planning protocols, decision-makers can better assess older adults’ needs and optimize their placement decisions, thereby facilitating appropriate transitions and promoting individualized care plans.

All in all, our findings can inform policy development, enhance the management of transitional care, and address patient flow inefficiencies by providing evidence-based insights into whether older adults should receive care in institutional settings or at home. Future research should address the identified limitations and gaps in the current literature to strengthen the evidence base for improving older adults’ transitions.

## Supplementary Information

Below is the link to the electronic supplementary material.

Supplementary Material 1

## Data Availability

No datasets were generated or analysed during the current study.

## Footnotes

1 The registration is currently under embargo and will be made publicly available upon publication (Registration ID: osf.io/eus5z).

## Abbreviations

ADL: Activities of Daily Living
BPSD: Behavioral and Psychological Symptoms of Dementia
CCC: Complex Continuing Care
CIHI: Canadian Institute for Health Information
COVID-19: Coronavirus Disease 2019
DASC-8: Dementia Assessment Sheet for Community-based Integrated Care
DPC: Diagnosis Procedure Combination
EHR: Electronic Health Records
GNRI: Geriatric Nutritional Risk Index
HIE: Health Information Exchange
LPN: Licensed Practical Nurse
LTC: Long-Term Care
MMSE: Mini-Mental State Examination
MoCA: Montreal Cognitive Assessment
OECD: Organization for Economic Co-operation and Development
PAC: Post-acute care
PCC: Population-Concept-Context
PRISMA-ScR: Preferred Reporting Items for Systematic reviews and Meta-Analyses extension for Scoping Reviews
QOF: Quality and Outcomes Framework
RN: Registered Nurse
RTCI: Return to Community Initiative
SDMM: Siebens Domain Management Model
TAVR: Transcatheter Aortic Valve Replacement
TBI: Traumatic Brain Injury
TKA: Total Knee Arthroplasty
TUG: Timed Up and Go
