# Supplementary material for: Shaping older adults’ care policy: a scoping review of key determinants in post-acute and community reintegration transitions
Source: BMC Health Serv Res. 2025 Sep 30;25:1236. doi: 10.1186/s12913-025-13433-x (PMC12482025; doi:10.1186/s12913-025-13433-x)
Supplement: Supplementary file 1 — Supplementary Material 1 [file 12913_2025_13433_MOESM1_ESM.docx]

# **Supplementary materials**

## **S1**. **Search strategy**

Table S1 represents the detailed search strategy for identifying relevant resources.

**Table S1.** Detailed search strategy

|  | Search terms |
| --- | --- |
| 1 | (elderly OR seniors OR geriatric* OR gerontolog* OR “old age” OR “older adults” OR “older persons” OR “older people” OR “older men” OR “older women” OR “older patients” OR “older inpatients”) |
| 2 | (transitions OR transfers OR “hand over” OR “hand-off” OR move OR admission OR admit* OR discharge) |
| 3 | (“acute care” OR hospital*) |
| 4 | (“long-term care” OR “nursing home” OR “assisted living” OR “supportive living” OR “supportive care”) |
| 5 | (“complex continuing care” OR “chronic care” OR “rehabilitation” OR “skilled nursing” OR “continuing care” OR “integrated care”) |
| 6 | (“home care” OR “community care” OR “home health nursing”) |
| 7 | (factors OR “associated factors” OR “underlying factors” OR “key factors” OR determinants OR predictors OR influences OR correlates OR barriers OR facilitators OR impacting OR “driving forces” OR “conditioning factors” OR characteristics OR variables OR attributes OR challenges OR circumstances OR context OR conditions) |
| 8 | 1 AND 2 AND 3 AND 4 AND 7 |
| 9 | 1 AND 2 AND 3 AND 5 AND 7 |
| 10 | 1 AND 2 AND 3 AND 6 AND 7 |
| 11 | 1 AND 2 AND 4 AND 6 AND 7 |
| 12 | 1 AND 2 AND 5 AND 6 AND 7 |

## **S2. Full list of included publications**

Table S2 provides a comprehensive list of the publications included in this scoping review.

**Table S2.** A complete list of publications included in the review

| **Title** | **First Author** | **Year of Publication** | **Analysis Period** | **Jurisdiction** | **Study Population** | **Research Design** | **Setting transitioned from** | **Setting transitioned to** | **Factors Associated with the Care Destination (positive effect)** | **Factors Associated with the Care Destination (negative effect)** |
| --- | --- | --- | --- | --- | --- | --- | --- | --- | --- | --- |
| Earlier physical therapy input is associated with a reduced length of hospital stay and reduced care needs on discharge in frail older inpatients: an observational study | Peter J. Hartley | 2019 | 2016 | UK | Frail older adults admitted to the Department of Medicine for the Elderly wards | Quantitative | Acute care | LTC | - | Early physical therapy assessment (within 24 hours of admission) |
| Complementing chronic frailty assessment at hospital admission with an electronic frailty index (FI-Laboratory) comprising routine blood test results | Hugh Logan Ellis | 2020 | 2015 to 2017 | UK | Older adults admitted to a large tertiary hospital. | Quantitative | Acute care | LTC/CCC | Frailty | - |
| Accuracy of the Clinical Frailty Scale for perioperative frailty screening: a prospective observational study | Jai N. Darvall | 2020 | 2017 | Australia | Patients aged 65 years and above undergoing elective and emergency surgery at the Royal Melbourne Hospital | Quantitative | Acute care | LTC/CCC | Frailty | - |
| Living settings and cognitive impairment are stronger predictors of nursing home admission after hip fracture surgery than physical comorbidities: A nationwide Danish cohort study | Liv Riisager Wahlsten | 2020 | 2005 to 2015 | Denmark | Community-dwelling Danish patients aged 60 to 100 years undergoing their first hip fracture surgery | Quantitative | Acute care | LTC | Age, Living alone, dementia, Preinjury home care, Parkinson’s disease, and depression | - |
| LTC provision, hospital bed blocking, and discharge destination for hip fracture and stroke patients | James Gaughan | 2017 | 2008 to 2009 | UK | Patients aged 65 and above admitted from home with hip fracture or stroke to NHS hospitals | Quantitative | Acute care | LTC | Age, female gender, number of diagnoses, number of additional procedures conducted during hospitalization | - |
| Stakeholder involvement in care transition planning for older adults and the factors guiding their decision-making: a scoping review | Sarah Carbone | 2022 | No date restriction | Multiple countries | Older adults 65 or over, caregivers and health professionals | Literature Review | Acute care | LTC/CCC/Home care | Institutional priorities and requirements, resources available for care and transition, knowledge of stakeholders, risk of transition, group structure and dynamic, health and support needs, personality preferences and beliefs | - |
| Ageing Well - School of Policy Studies - Queen’s University | Don Drummond | 2020 | Not applicable (policy report) | Canada (Particularly Ontario) | Elderly Canadians, specifically those with dementia and frailty. | Policy analysis and recommendations | Institution (Hospital, LTC, CCC) | LTC/CCC/Home care | Positive on going to LTC: Dementia, frailty, falls, caregiver distress, medical instability, Assessment location (assessed in hospital than if assessed in a community setting), need assistance with ADL Positive on going to home care: Availability of home care services, community support, communal living arrangements, policy support for aging in place | - |
| Evidence-based considerations around LTC | McMaster University | 2022 | Not applicable | Canada | Seniors, particularly those considering transition to LTC facilities | Review and synthesis of evidence-based practices | Acute care | LTC | Serious functional and cognitive impairments (such as dementia), comorbidities, using several medications, having previously been hospitalized or temporarily placed in another care facility, caregiver stress, local healthcare system conditions, availability of LTC beds, and the standards determining how available beds are distributed | - |
| The future of LTC requires investment in both facility- and home-based services | David C. Grabowski | 2021 | Not explicitly mentioned. | USA, with comparisons to other OECD countries | Seniors requiring continuing care services, particularly those in LTC homes. | - | LTC | Home care | The preferences of the individual and their family members for home-based models, more government spending on home-based models and prioritizing it (The Dutch, Norwegian and Swedish systems of prioritizing HCBS is the better approach, but this approach is not cheap.) | specific health concerns like cognitive issues. lack a home to receive care in the community, isolation and loneliness in the community |
| Gender Differences in Institutional LTC Transitions | Stipica Mudrazija | 2015 | 2000-2010 | USA | 3,351 respondents aged 65 and older admitted to LTC (LTC) facilities | Quantitative | LTC | Home care | Younger age, Higher use of rehabilitation services, Family ties (e.g., living children), Being female, staying less than one year, Hispanic origin (for men, short-term stay), Non-Hispanic Black identity (for women, short-term stay), Larger household size (men, short-term; women, long-term), Use of special facilities or services (women only) Overnight hospital stays (women only), Low education (for women, long-term stay) | being male, Cognitive issues, Memory-related problems, including Alzheimers and dementia (more significant for men) |
| Community discharge of nursing home residents: the role of facility characteristics | Amanda A. Holup | 2016 | July 2007-June 2008 | USA (California and Florida) | LTC residents in California (n = 1,127) and Florida (n=657) | Quantitative | LTC | Home care | Larger Facility size (number of beds), Higher occupancy rate, For-profit ownership (only long-stay residents), Higher proportion of residents admitted from acute care facilities (California), higher proportion of Medicare-funded residents (long-stay), facilities located in more densely populated markets (Florida long-stay population), markets with a greater NH concentration (Florida long-stay population) | Longer average length of stay, Higher proportion of Medicaid residents, Cognitive impairment ADL dependencies, Behavioural problems |
| Shall I Stay or Shall I Go? The Choice to Remain in the Nursing Home Among Residents with High Potential for Discharge | Kathleen Abrahamson | 2020 | July 2015 to June 2016 | USA | Private paying residents admitted to Minnesota nursing facilities who expressed a desire for discharge, but chose to stay in the facility | Quantitative | LTC | Home care | - | Age, Moderate to severe Cognitive impairment, unmarried status, Behavioural problems, Diagnosis of dementia or Alzheimer’s, Dependency in ADL |
| Sizing Up the Challenge. Meeting the Demand for LTC in Canada | Robyn Gibbard | 2017 | 2015 to 2016 | Canada | Seniors (particularly those aged 75 and up) requiring LTC | - | hospital | LTC | Age, the presence of acute conditions, the availability of LTC beds, the cost of care in different settings (hospitals vs. LTC homes), waitlist status | |
| Frailty in an Older Inpatient Population: Using the Clinical Frailty Scale to Predict Patient Outcomes | David Basic | 2015 | 2010 to 2014 | Australia | older adults admitted under the care of eight geriatricians. | Quantitative | Acute care | LTC | Age, Frailty (CSHA-CFS), urine retention, deconditioning, dementia, comorbidities | - |
| Clinical predictors of protracted length of stay in Ontario CCC hospitals | Luke A. Turcotte | 2019 | March 31, 2001, to March 31, 2013 | Canada | Patients admitted to CCC hospitals in Ontario, with a sample size of 91,113 episodes of care. | Quantitative | CCC | Home care | female sex, older age, higher levels of medical instability, increasing number of recent hospitals stays and emergency department visits, cancer diagnosis, pneumonia, unsteady gait, patients that desired to return to the community, and having a support person who was positive towards discharge. | increasing levels of functional impairment, cognitive impairment, pressure ulcer risk, hemiplegia, traumatic brain injury, aphasia, antibiotic resistant infection, HIV infection, feeding tube, dialysis, psychological therapy, tracheostomy, ventilator, respirator |
| Factors Associated With Discharge Destination in Community-Dwelling Adults Admitted to Acute General Medical Units | Aruska N. D’Souza | 2020 | July 2016 to August 2017 | Australia | Inpatients admitted to the general medical unit of a tertiary hospital and referred to physical therapy | Quantitative | Acute care | home care | higher functional scores, higher mobility scores, higher cognitive scores, shorter length of stay, lower incidence of falls in the past 6 months |  |
| Challenges to community transitions through Money Follows the Person | Julie Robison | 2020 | December 2008 to December 2017 | USA | Individuals enrolled in Connecticut’s MFP program, including older adults, people with mental health disabilities, and people with physical disabilities. | Quantitative | LTC | Home care | non-white and Hispanic race, facility-related or services/supports challenges | Higher age, longer length of stay, Consumer engagement challenges, housing challenges, issues with family members or unpaid caregivers, mental health disability, physical health challenges, cognitive issues, dementia, |
| Experiences of Informal Caregivers of Older Adults Transitioned From Nursing Homes to the Community Through the Money Follows the Person Demonstration | Lorand Kristof | 2017 | September 2011 to November 2013 | USA | Informal caregivers of older adults who transitioned to the community living through the Connecticut Money Follows the Person (MFP) after prolonged nursing home stays | Mixed methods | LTC | Home care | Caregiver satisfaction with services, person-centred care plans. | high Unmet service needs, High degree of assistance needed with Activities of Daily Living (ADLs) and Instrumental Activities of Daily Living (IADLs), high caregiver burden. Depressive symptoms |
| Risk factors associated with residential aged care, respite and transitional aged care admission for older people following an injury-related hospitalisation | Rebecca Mitchell | 2017 | July 1, 2008 to June 30, 2013 | Australia | Individuals aged ≥65 years who had an injury hospitalisation in New South Wales, Australia. | Quantitative | Acute care | LTC | Higher age, female, not married, had more than one comorbidity, dementia, hospitalised following a fall, injuries to the hip and thigh, Age-adjusted mean hospital LOS | - |
| Association of Functional and Cognitive Impairment Severity with Discharge to LTC Facilities in Older Patients Admitted to a General Acute Care Hospital from Home | Seigo Mitsutake | 2023 | July 2016 to December 2018 | Japan | Patients aged ≥65 years discharged from a general acute care hospital in Japan, with a sample size of 9,060. | Quantitative | Acute care | LTC | Moderate to severe dementia and/or BADL impairment (Category III on DASC-8), Higher age (≥85 years) High frailty risk (HFRS ≥5) | Higher BI scores (Higher functional independence) |
| A myriad of factors influencing the implementation of transitional care innovations: a scoping review | Amal Fakha | 2021 | 2000 to 2020 | International | Older persons (aged 65 years and above) and healthcare professionals involved in transitional care. | Literature review | Hospital | Home care | High-perceived advantage of the innovation, encouraging transition roles, continuous monitoring | Low organizational readiness, poor implementation climate, targeting the wrong population group, skilled users with restricted knowledge and mixed attitudes about the innovation |
| Do financial aspects affect care transitions in LTC systems? A systematic review | Estera Wieczorek | 2022 | 2005 to 2020 | International | Older adults (aged 60 years and above) | Literature review | All settings | All settings | reimbursement mechanism, reward, and penalty | - |
| Assessing the impact of Minnesota’s return to community initiative for newly admitted nursing home residents | Zachary Hass | 2019 | 2014 to 2016 | USA | Non-Medicaid nursing home admissions in Minnesota who remained in the nursing home for at least 45 days | Quantitative | LTC | Home care | RTCI Score, shorter length of stay, higher facility participation rates | Pneumonia, feeding tube |
| A Statewide Model for Assisting Nursing Home Residents to Transition Successfully to the Community | Darci Buttke | 2018 | 2012 to 2016 | USA | Nursing home residents, primarily private-paying ones, who are part of the Return to Community Initiative (RTCI) | Mixed methods | LTC | Home care | Health status at the time of discharge, functional ability, fit a community discharge, Availability of a primary caregiver, resident’s own desire to return to the community, entering the nursing facility directly from a hospital | concerns about access to healthcare and personal safety in the community |
| Seniors in Transition: Exploring Pathways Across the Care Continuum | Kim Nuernberger (CIHI) | 2017 | 2012 to 2015 | Canada | Seniors aged 65 and older receiving publicly funded continuing care services | Quantitative | Hospital | LTC | Initial assessment in the hospital, requires physical assistance, Cognitive impairment, wandering, Living Alone, Caregiver unable to continue, caregiver distress, Wandering, falls, medical instability, and a diagnosis of dementia | - |
| Modelling Community Discharge of Medicaid Nursing Home Residents: Implications for Money Follows the Person | Zachary Hass | 2018 | 2011 to 2013 | USA | Medicaid-eligible nursing home residents in Minnesota (33,590 nursing home stays that qualified for Medicaid by the 90th day of their stay from 383 Minnesota nursing homes). | Quantitative | LTC | Home care | Preference for community discharge as indicated by the resident at the time of admission, Relatively better health (not being in the end stages of a disease and no heart failure diagnosis), younger than age 85, more functionally independent (lower ADL score), continent, no recent falls, having had a hip fracture, being cognitively intact, without a dementia diagnosis, absent behavioural problems, admitted from acute care and with a rehabilitation stay, and the female gender | length of stay |
| Drama and Trauma: Unpacking Moral Distress in the Context of Discharge Planning | Kristi L. Kirschner | 2020 | 2020 | USA | Medicaid-eligible nursing home residents in Minnesota (33,590 nursing home stays that qualified for Medicaid by the 90th day of their stay from 383 Minnesota nursing homes). | Qualitative | Hospital | Home care | - | Patient and Caregiver-level Concerns:  unilateral decision-making by healthcare teams, Feelings of abandonment and frustration.  Feeling poorly prepared for discharge.  Lack of information and resources.  Discordance between medical and social needs, Pressure to accept nursing home placement.  Lack of reasonable choices for home and community-based care. Issues related to dignity, autonomy, and safety.  Poor communication and lack of education about discharge.  Structural Barriers:  Financial pressures and insurance limitations. |
| Association Between Community Transition and Independence and Control Over Life: Analysis of Georgia’s Money Follows the Person Program | Farah Naz Sulaiman | 2017 | 2008 to 2015 | USA | The study includes 664 participants who are part of Georgia’s Money Follows the Person (MFP) program, with a male to female ratio of 54.4% to 45.6%. | Quantitative | LTC | Home care | Being able to pick the place of residence, Age, going to bed when desired, eating whenever preferred, choosing type of food, privacy on phone, engaging in community activities, doing voluntary work, picking care providers | - |
| Geriatric nutritional risk index and 100-m walk achievement predict discharge to home in elderly patients with heart failure | Shota Shimoyama | 2020 | 2014 to 2018 | Japan | 165 elderly patients with heart failure aged at least 75 years, who lived at home before admission to the institution. | Quantitative | Hospital | Home care | 100-m walk achievement and the Geriatric Nutritional Risk Index (GNRI) at 2 weeks after admission | - |
| Machine learning prediction of hospital patient need for post-acute care using an admission mobility measure is robust across patient diagnoses | Daniel L. Young | 2023 | 2016 to 2019 | USA | A retrospective cohort of 34,432 patient admissions to two hospitals within Johns Hopkins Medicine, Johns Hopkins Hospital and Johns Hopkins Bayview Hospital. | Quantitative | Hospital | LTC, CCC | lower mobility scores (specifically AM-PAC scores), higher BMI, age | - |
| Improving the post-acute care discharge score (PACD) by adding patients’ self-care abilities: A prospective cohort study | Daniel Koch | 2019 | February 2013 to October 2013 | Switzerland | Consecutive adult medical and neurological inpatients at KSA, a 600-bed tertiary hospital in the Canton of Aargau, Switzerland | Quantitative | Hospital | LTC, CCC | Lower self-care index scores, older age, longer hospital stay, a higher post-acute care discharge score (number of medically active problems on admission, number of disabilities, age) | - |
| Association Between Therapy Intensity and Discharge Outcomes in Aged Medicare Skilled Nursing Facilities Admissions | Suzanne R. O’Brien | 2018 | 2008 | USA | The study population includes aged Medicare fee-for-service beneficiaries in skilled nursing facilities (SNFs). The final analytical sample consisted of 311,338 admissions (80% of the total) to 3605 SNFs. | Quantitative | SNF (CCC) | Home care | Therapy Intensity, physical therapy, occupational therapy, and speech therapy | - |
| Transitions in Care in a Nationally Representative Sample of Older Americans with Dementia | Christopher M. Callahan | 2015 | 1999 to 2008 | USA | HRS (Health and Retirement Study) respondents aged 65 and older whose survey data were linked with Medicare claims (N = 16,186). | Quantitative | Hospital | LTC | severity of dementia, availability of formal services at home | - |
| Medicaid home- and community-based services and discharge from skilled nursing facilities | Sijiu Wang | 2021 | 2010 to 2013 | USA | The study includes 224,229 community-dwelling older adults (aged 65 and above) who were dually enrolled in Medicare and Medicaid and were newly admitted to skilled nursing facilities (SNFs) following an acute inpatient event. | Quantitative | All settings | All settings | Generosity of Medicaid home- and community-based services (HCBS), measured in terms of breadth and intensity, Individual factors, including age | - |
| Medicaid LTC Policies and Rates of Nursing Home Successful Discharge to Community | Huiwen Xu MHA | 2020 | 2014 to 2017 | USA | 11,694 unique nursing homes in the United States | Quantitative | LTC | Home care | Higher state Medicaid spending on home and community-based service (HCBS), more generous reimbursements to nursing homes, absence of bed-hold policies, higher penetration of Medicare Advantage, , higher % older population, higher median family income, presence of Alzheimer’s unit, chain affiliation, higher percentage of Medicare residents, RN to total nurse staffing ratio, any nurse practitioner or physician’s assistant | rural location of nursing home, NHs in markets with stronger competition, for-profit NH, higher % Medicaid residents, |
| Facility-Level Factors and Outcomes After Skilled Nursing Facility Admission for Trauma and Surgical Patients | Lucas W. Thornblade | 2018 | 2007 to 2009 | USA | 389,133 patients (mean age 78 years, 63% female) who were discharged to Medicare-certified skilled nursing facilities (SNFs) after trauma or major surgery. | Quantitative | SNF (CCC) | Home care | Lower Bed: Nurse ratios, greater density of specialty patients (surgery, trauma) | higher ratio of LPNs to RNs |
| Effect of Obesity on Postacute Outcomes of Skilled Nursing Facility Residents with Hip Fracture | Cyrus M. Kosar | 2018 | 2008 to 2015 | USA | Medicare fee-for-service beneficiaries discharged to a skilled nursing facility after hospitalization for hip fracture. Total population of 586,683 individuals, of which 82,768 (14.1%) met the obesity criteria. | Quantitative | SNF (CCC) | Home care | - | Level of obesity |
| Successful Community Discharge Among Older Adults with Traumatic Brain Injury in Skilled Nursing Facilities | Emily Evans | 2021 | 2011 to 2015 | USA | Medicare fee-for-service beneficiaries admitted to a SNF after hospitalization for TBI. | Quantitative | SNF (CCC) | Home care | race other than white and Black (Asian, Hispanic, North American Native) | Medicaid enrolment, incontinence, decreased independence with ADL, and cognitive impairment |
| Successful Community Discharge Among Older Adults With Traumatic Brain Injury Admitted to Inpatient Rehabilitation Facilities | Emily Evans | 2021 | 2011 to 2015 | USA | Community-dwelling adults aged 66 years and older, who were hospitalized after TBI and then admitted to an IRF. The sample included a mean of 1060 individuals across 30 linked datasets | Quantitative | CCC | Home care | Functional Independence Measure motor (FIM-M) and cognitive (FIM-C) scores, | pre-injury chronic conditions, and pre-injury living arrangement (alone) |
| Association of Fragmented Readmissions and Electronic Information Sharing With Discharge Destination Among Older Adults | Sara D. Turbow | 2023 | 2018 | USA | The cohort included 275,189 admission-readmission pairs, representing 268,768 unique patients | Quantitative | Acute care | Home care | Sharing health information between the admission and readmission hospitals via Health Information Exchange (HIE) | Fragmented readmissions |
| Living Alone and Discharge to Skilled Nursing Facility Care after Hospitalization in Older Adults | Daniel E. Lage | 2017 | 2014 to 2015 | USA | Community-dwelling individuals aged 50 and older who were admitted to the medical service and discharged alive between July 2014 and August 2015 (N = 7,029) | Quantitative | Hospital | SNF (CCC) | Living alone, higher age, length of stay, impaired mobility, impaired bathing, dual eligibility on insurance, urinary tract infection | Asian or African American, having a diagnosis of congestive heart failure, arrythmia, myocardial infraction, coronary artery disease |
| Hospital to Home: Supporting the Transition From Hospital to Home for Older Adults | Brittany Barber | 2022 | December 2019 to February 2020 | Canada | Older adults who were in an acute care unit waiting for placement in LTC facilities, their family or friend caregivers, and healthcare professionals involved in their care. | Qualitative | Acute care | Home care | - | Lack of timely information and communication about home care services, Caregiver burnout, and stress due to lack of support.  Insufficient home care services, particularly for overnight support. Reliance on unpaid caregivers without providing them with adequate resources and support.  Delays and barriers in accessing LTC.  Challenges in communicating between family caregivers and healthcare professionals.  late introduction of home care services, financial strains on caregivers |
| New care home admission following hospitalisation: How do older people, families and professionals make decisions about discharge destination? A case study narrative analysis | Sarah J Rhynas | 2018 | November 2013 to February 2015 | UK | A purposive sample of 10 cases selected from a cohort of 100 individuals admitted to a hospital from home and discharged to a care home. | Qualitative | Acute care | LTC | Family support | - |
| Risk of Care Home Placement following Acute Hospital Admission: Effects of a Pay-for-Performance Scheme for Dementia | Panagiotis Kasteridis | 2016 | 2006/07 to 2010/11 | UK | Individuals with dementia who were admitted to a hospital with a primary diagnosis of dementia or those who were admitted for treatment of an ambulatory care sensitive condition. | Quantitative | Acute care | LTC | older age, female gender, vascular dementia, incontinence, falls, hip fracture, and the number of comorbidities | - |
| Evaluating Siebens Domain Management Model for inpatient rehabilitation to increase functional independence and discharge rate to home in geriatric patients | David S Kushner | 2015 | 2010 to 2012 | USA | Geriatric patients aged 75 and above who were admitted to an Inpatient Rehabilitation (IR) facility in 2010 and 2012 | Quantitative | CCC | Home care | The implementation of the Siebens Domain Management Model (SDMM) which focuses on potential barriers to discharge home. | - |
| Evidence Brief Improving Hospital-To-Home Transitions for Older Adults with Complex Health and Social Needs in Ontario | McMaster University Health Forum | 2020 | - | Canada | Older adults with complex health and social needs transitioning from hospital to home, and their caregivers. | Systematic review of existing research evidence and synthesis of stakeholder insights | Acute care | Home care | . | many older adults have a wide range of complex health and social needs that make hospital-to-home, transitions complex and risky;, despite a growing body of research evidence about the experiences of older adults with complex health and social needs (and their caregivers) during hospital-to-home transitions, there is still a knowledge- to-practice gap, caregivers often feel unprepared to support hospital-to-home transitions; o the health system is not currently designed to support older adults with complex health and social needs during hospital-to-home transitions; the pressures being placed on health and social systems to respond to the, ongoing COVID-19, pandemic affect hospital-to-home transitions; and, bringing about system changes takes time,, resources and commitment from many players, including, older adults, family caregivers, providers, organizations and the system as a whole. |
| Moving to a new home in continuing care An information and decision-making guide for patients and families | Alberta health services continuing care | 2019 | - | Canada | Individuals transitioning to continuing care facilities (mainly seniors or individuals with specific health care needs). |  | Hospital | LTC, CCC | comprehensive assessment of individual’s physical function, mental health, social abilities, financial situation, and health care needs. The guide also mentions the importance of discussing needs with family members and case managers and they being actively involved in the assessment process to communicate preferences and needs, number of people on the waitlist, size of the facility and how frequently a room becomes available at the facility | - |
| Nursing’s Role in Successful Transitions Across Settings | Michelle Camicia | 2016 | - | USA | Stroke patients transitioning from acute care to various post-acute care settings | - | Hospital | Home care | Effective information transfer that contributes to optimal collaboration and coordination among the patient, family, and interprofessional team, family caregiver involvement and capacity, nurse-led transitional care programs (Nurses interact with patients/families at their most vulnerable times and often learn information critical to successful transition planning), early identification and communication of transition issues, including medication management in the transition plan (assessing patients medication, education and counselling about side effects) | Poor communication and coordination, insufficient preparation and support for family caregivers, limited finances |
| From facility to home: How healthcare could shift by 2025 | Oleg Bestsennyy | 2022 | - | USA | Elderly patients or patients with chronic conditions who might be eligible for Care at Home services. | Qualitative | Hospital | Home care | Growth in virtual care, emergence of new technologies, investment in the digital health market, economic viability of Care at Home, physician awareness and perceptions, patient preferences, and policy changes in healthcare reimbursement. | - |
| Factors associated with non-home discharge of patients hospitalized for hip fracture: A nationwide retrospective study using the Japanese diagnostic procedure combination database | Mutsuko Moriwaki | 2023 | April 2018-March 2019 | Japan | Patients aged ≥ 65 years who were hospitalized and discharged between April 2018 and March 2019 | Quantitative | Acute care | LTC, CCC | Age (75 to 84 years and ≥85 years), electrocardiography or respiratory treatment, level of assistance with activities of daily living, and hospital where the patient-to-nurse ratio is 7:1 | - |
| A Scoping Review of Care Trajectories across Multiple Settings for Persons with Dementia | Julie G. Kosteniuk | 2021 | 2007-2017 | Multiple countries | Persons with dementia (age >= 65) | Literature Review | Hospital | LTC | Widowhood, older age, female sex, severity of dementia, co-morbidity, severity of medical issues, falls, and hip fractures, incontinence, cerebrovascular disease, and cancer, percutaneous endoscopic gastrostomy, tube insertion in hospital, greater functional disability, and diabetes, behavioural and psychological  symptoms associated with dementia | - |
| Beyond Clinical Complexity: Nonmedical Barriers to Nursing Home Care for Rural Residents | Carrie Henning-Smith | 2018 | Not Specified | USA | Rural hospital discharge planners | Qualitative | Hospital | LTC | - | financial issues, transportation barriers, nursing home availability and infrastructure, timeliness (mention in rural areas) |
| Care patterns and predictors of community residence among older patients after hospital discharge for traumatic brain injury | Monique R. Pappadis | 2023 | 2014 to 2017 | U.S. | Medicare fee-for-service patients older than 65 years hospitalized for TBI in Texas | Quantitative | Acute care | Home care | Female sex  Hispanic ethnicity  “Other” race category (e.g., Asian/Pacific Islander), having a Prior primary care provider (PCP) prior to hospitalization | Age 75 and above  Prior nursing home (NH) residence, Dual eligibility (Medicare and Medicaid), Prior traumatic brain injury (TBI) diagnosis, Moderate-to-severe trauma injury severity |
| Determining discharge destination in geriatric evaluation and management units: Is progressive goal attainment a better early indicator of discharge destination than improvement in functional independence measure scores? | Susan Black | 2018 | Not Specified | Australia | 82 patients admitted to the hospital’s GEM Unit | Quantitative | CCC | Home care | positive effect on going to home: goal-attainment (patients who achieved their initial short-term goals were highly likely to be discharged home), | - |
| Development and validation of prediction models for the discharge destination of elderly patients with aspiration pneumonia | Yoshito Hirota | 2023 | April 2020-March 2021 | Japan | patients aged 65 years and older who were admitted to and discharged from the study hospitals between April 1, 2020, and March 31, 2021, and who were recorded as having aspiration pneumonia. | Quantitative | Hospital | Home care | BMI >25 | Negative effect on going home: older age, male gender, BMI lower than 18.5, needing assistance with at least one of the ten ADL functions of the Barthel index, malnutrition, impaired level of consciousness on the Japan coma scale, metastatic cancer, dementia, pressure ulcers, pleural effusion, oxygen administration, respiratory support, vasopressor administration, and sputum suctioning |
| Discharge from hospital – a national survey of transition to out-patient care | Elizabeth Åhsberg | 2018 | 2014 | Sweden | Patients discharged from Swedish hospitals in 2014, with a focus on those in need of further out-patient medical care and/or social services | Mixed methods | Hospital | LTC | individual planning, geriatric assessment, support and patient education | a decreasing number of beds in hospitals and nursing homes, lack of staff with proper education, and problems in the transfer of information between caregivers. |
| Disparities in Discharge Destination After Lower Extremity Joint Arthroplasty: Analysis of 7924 Patients in an Urban Setting | Ifeoma A. Inneh | 2016 | 2011 to 2014 | USA | 7924 patients admitted between 2011 and 2014 for primary or revision hip or knee arthroplasty at a single institution. | Quantitative | Hospital | Home care | Non-Black race/ethnicity | low to middle socioeconomic status, age, female gender, and undergoing a total knee arthroplasty procedure. |
| Early Predictors for Discharge to Geriatric Rehabilitation after Hip Fracture Treatment of Older Patients | Dieuwke van Dartel | 2021 | 2017 to 2019 | Netherlands | 21,176 patients with hip fracture aged 70 years and older. | Quantitative | Hospital | LTC, CCC | positive on going to CCC: Higher age, poor premorbid mobility, lower premorbid Katz-ADL, no history of dementia, ASA score 3-5, general anaesthesia, intramedullary implant, and cotreatment by a geriatrician Positive on going to LTC: Higher age, poor premorbid mobility, history of dementia, higher premorbid Katz - ADL, ASA score 3-5, general anaesthesia, intramedullary implant, and cotreatment by a geriatrician | - |
| Effectiveness and analysis of factors predictive of discharge to home in a 4-year cohort in a residential transitional care unit | Daniel Kam Yin Chan | 2019 | 1 January 2014 to 31 December 2017 | Australia | patients admitted to the r-TACP unit during the study period. The majority of these patients were aged 65 years or older. | Quantitative | CCC | Home care | - | Age, Greater comorbidities (the Charlson Comorbidity Index (CCI)) and poorer baseline functional status (lower Barthel index on admission to CCC) |
| Factors Affecting Discharge to Home of Geriatric Intermediate Care Facility Residents in Japan | Kojiro Morita | 2018 | April 2012–March 2014 | Japan | 342,758 individuals newly admitted to 3,459 geriatric intermediate care facilities during the study period. | Quantitative | LTC | Home care | Male gender, using home-based services 1 month before admission, underwent rehabilitation service, being in facilities with more staff members per 100 beds | Older age, higher level of care need, having several medical conditions (including dementia and dysphagia), private ownership of the facility, more beds in the facility, and more LTC facility beds per 1,000 adults aged 65 and older in the region |
| Factors affecting residents transition from long term care facilities to the community: a scoping review | Shannon Freeman | 2017 | 2000 to 2015 | Different countries | Residents of LTC facilities who have the potential to transition to community living. | Literature Review | LTC | Home care | Younger age, married, female, having higher cognitive and physical abilities, received intense therapy, having a preference for discharge to the community, recently had fractures, medically stable, fit a community discharge profile, supported by LTCF staff, consistency of LTCF care, staff respect and dignity for the person, receiving independent living training or community skills training, caregiver support, medication management, available community supports for ADL | cancer, cognitive impairment |
| Factors influencing home discharge after inpatient rehabilitation of older patients: a systematic review | Irma H. J. Everink | 2016 | 2000 to 2015 | Different OECD countries | older non-stroke patients undergoing rehabilitation | Literature Review | CCC | Home care | Younger age, Non-white ethnicity, Being married, better functional and cognitive status, absence of depression, less clinical severity of the illness, No active cardiac pathology | Older age, white ethnicity, being single or not married, poor functional and cognitive status, presence of depression, greater clinical severity of the illness, active cardiac pathology |
| Frailty independently predicts unfavorable discharge in non-operative traumatic brain injury: A retrospective single-institution cohort study | Rahul A. Sastry | 2022 | 2020 to 2021 | USA | All patients aged 70 or older admitted from home to the neurosurgical service of a single institution for non-operative TBI | Quantitative | Hospital | LTC, CCC | High frailty | - |
| Health, Social, and Functional Characteristics of Older Adults with Continuing Care Needs: Implications for Integrated Care. | Cara Brown | 2019 | 2014 to 2016 | Canada | Older, hospitalized adults who required continuing care on discharge (N = 214) | Quantitative | Acute care | LTC, CCC | Mental and behavioural issues, living alone, functional status, and preadmission concerns about the patient management in the community (needing assistance with toileting and mobilizing), vision or hearing difficulties | - |
| Healthcare professionals’ perception of barriers and facilitators for care coordination of older adults with complex care needs being discharged from hospital: A qualitative comparative study of two Nordic capitals | Janne Agerholm | 2023 | 2018 to 2019 | Denmark and Sweden | 5 nurses and 2 assistant nurses involved in the coordination of the discharge process at hospitals or in the home healthcare services (Copenhagen n = 11, Stockholm n = 16) | Qualitative | Acute care | Home care | Regular visits by municipal discharge coordinators, shared information system, follow-home nurse, ( Municipal discharge coordinators visiting patients at the hospital before discharge and the follow-home nurse were seen as facilitators in Copenhagen.  In Stockholm, the shared information system with access to patient records was lifted as a facilitator for coordination.  Staff influence on coordination and information: The staff’s possibility to influence the coordination and the information provided.  System knowledge: Knowledge about the system and the working conditions/responsibility of other actors.) | Lack of information in care plans (Insufficient information/communication regarding the patients’ care needs after discharge.)  Difficulty accessing collaborators (Difficulties accessing collaborators.  Uncertain responsibility: Uncertainty about who is responsible in specific situations.)  Work pressure on primary care nurses(Nursing staff at hospitals sometimes faced extreme challenges getting access to and collaborating with patients’ primary care clinics) |
| Hospital Discharge Decisions Concerning Older Patients: Understanding the Underlying Process | Pierre Koskas | 2019 | 2016 | France | All hospitalized patients >65 year at Bretonneau Hospital | Mixed methods | Acute care | LTC | Isolation, social problems, refusal of care, behavioural symptoms perceived as aggressive | Reliable family caregivers, social support from family or professional home service |
| Improved Prediction of Older Adult Discharge After Trauma Using a Novel Machine Learning Paradigm | Rachel S. Morris | 2022 | 2007 to 2014 | USA | Individuals >=65 admitted to a U.S. trauma centre for definitive care | Quantitative | Acute care | Home care | younger age, higher GCS score (Glasgow Coma Scale), the absence of a cervical spine fracture, a pelvic fracture or a hip fracture | - |
| No Other Safe Care Option: Nursing Home Admission as a Last Resort Strategy | Heather J Campbell-Enns | 2020 | Not Specified | Canada | 13 lower-care residents living in nursing homes and 13 family members of these residents. | Qualitative interpretive description methodology | LTC | Home care | specialized supports targeting mental health and substance use needs, enhanced hospital discharge plans, improved information about community-based care options | Isolation, physical and cognitive decline, anxiety and depression, substance use, insufficient family caregiving support, limited and inflexible community services, ineffective discharge procedures from hospitals, lack of information regarding services and processes for accessing care |
| Nursing Home Utilization following Implantable Cardioverter- Defibrillator Implantation in Older Patients: Results from the NCDR | Daniel B. Kramer, | 2017 | 2006 to 2010 | USA | 192,483 patients > 65 years receiving implantable cardioverter-defibrillators between January 1, 2006 – March 31, 2010 | Quantitative | Hospital | LTC | older age, dementia, and recent prior NH stay, class IV heart failure, | - |
| Positive Beliefs and the Likelihood of Successful Community Discharge From Skilled Nursing Facilities | Emily Evans | 2021 | 2011 to 2016 | USA | Fee-for-service Medicare beneficiaries (N=526,432) aged 66 years or older who were discharged to an SNF after hospitalization for stroke, hip fracture, or traumatic brain injury | Quantitative | SNF (CCC) | Home care | Positive patient beliefs about their capability to increase independence with ADLs, Positive staff beliefs about patient capability | Negative patient beliefs about their capability to increase independence with ADLs, Negative staff beliefs about patient capability |
| Predicting discharge to institutional LTC after hospital admission: a systematic review and meta-analysis | Jennifer Harrison | 2017 | - | Multiple countries | 354,985 participants admitted directly to long-term institutional care from the acute hospital | Literature Review | Acute care | LTC | Older age, female sex, dementia, functional dependency, malnutrition, unmarried status | - |
| Predicting factors of elderly patients’ discharge to home after rehabilitation in rural Japan: a retrospective cohort study. | Ryuichi Ohta | 2021 | 2016 to 2020 | Japan | 783 consecutive patients aged over 65 years with frailty and multimorbidity who were admitted with acute diseases and underwent active rehabilitation at a rural community hospital. | Quantitative | CCC | Home care | A high score in the motor component of the functional independence measure (FIM), shorter duration of stay |  |
| Predictors of home discharge among patients hospitalized for behavioural and psychological symptoms of dementia | Shinnichi Tochimoto | 2015 | April 2006 to March 2011 | Japan | 391 Consecutive patients who were admitted to the acute psychogeriatric ward of Ishikawa Prefectural Takamatsu Hospital from their own home for the treatment of BPSD | Quantitative | Acute care | Home care | High Mini-Mental State Examination, High Nishimura-style senile activities of daily living | Living alone and manifestation of aggressiveness at the time of admission |
| Calling time on the ‘dance of the blind reflex’: how collaborative working reduced older persons’ length of stay in acute care and increased home discharge | Dolores Donegan | 2021 | 2018 to 2019 | Ireland | acute hospital patients aged over 65 years | Practice development project | Hospital | Home care | an integrated system of care based on a combination of Lean Six Sigma and person-centred approaches, Creating a shared vision across all services that puts the patient at the centre of care supports, patients and families to choose and achieve their care preferences, overall, these are the factors (Improved communication within and among services, and with patients and their families. Enhanced patient and family involvement in care planning. Use of Lean Six Sigma to streamline discharge processes. Increased clarity of roles among healthcare providers.) | - |
| Rural/urban differences in discharge from rehabilitation in older adults with traumatic brain injury. | Anderson, Marie C | 2021 | 2011 to 2015 | USA | Medicare beneficiaries aged 66 and older discharged to a SNF following hospitalization (n = 61,021) | Quantitative | SNF (CCC) | Home care | - | Rurality |
| The influence of socio-demographic factors and close relatives at hospital discharge and post hospital care of older people with complex care needs: nurses’ perceptions on health inequity in three Nordic cities | A. E. M. Liljas | 2022 | 2018 to 2019 | Sweden and Denmark | Healthcare professionals (nurses) involved in the hospital discharge process of older adults with complex care needs in Stockholm, Copenhagen, and Tampere. | Qualitative | Hospital | Home care | Close relatives providing support and information (information about their older relative’s everyday life and home situation) | Language barriers (especially for patients from ethnic minorities), Socioeconomic barriers (inability to afford care costs, medications, and home help), unfavourable home situations (sanitation of the patient’s home, changes to home environment), Poor collaboration between healthcare professionals at different sites, patient’s health, functional, and cognitive status, Inequities in local healthcare resources and collaboration (differences in care quality based on geographic location), |
| Factors Influencing Discharge Destination After Total Knee Arthroplasty: A Database Analysis | Ran Schwarzkopf | 2016 | 2010 | USA | 28 611 patients undergoing TKA hospitalized in the state of California | Quantitative | Acute care | LTC/CCC | Increased Charlson Index, age, Asian ethnicity, Black ethnicity, Hispanic ethnicity, being a Medicare patient | Male gender |
| Nonhome Discharge in Patients Undergoing Pelvic Reconstructive Surgery: A National Analysis | James H Ross | 2023 | 2010 to 2018 | USA | 38,012 patients who underwent Sacro colpopexy, vaginal colpopexy, and colocalises | Quantitative | Acute care | LTC, CCC | preoperative weight loss, dependent health care status, abdominal hysterectomy, American Society of Anaesthesiologists class 3 or greater, age, operative time, laparoscopic hysterectomy, laparoscopic Sacro colpopexy | - |
| Risk scoring model for prediction of non-home discharge after transcatheter aortic valve replacement | Alexis K Okoh | 2020 | June 2012 to December 2018 | USA | 1126 patients discharged alive after transcatheter aortic valve replacement | Quantitative | Acute care | LTC/CCC | extreme age, female sex, elective procedures, history of CABG, dyslipidaemia, chronic liver disease, history of pacemaker, non-transfemoral approach, postoperative complication | - |
| Rehabilitation Providers’ Prediction of the Likely Success of the SNF-to-Home Transition Differs by Discipline | Adam Simning | 2019 | 2016 to 2017 | USA | One hundred-twelve English-speaking adults aged 65 years and older admitted to 2 SNF rehabilitation units | Quantitative | SNF (CCC) | Home care | - | living alone, more medical conditions, lower physical functioning scores, and greater depression scores, occupational and physical therapists’ neutral or negative predictions of post SNF discharge outcomes |
| Using population-based routinely collected data from the Sentinel Stroke National Audit Programme to investigate factors associated with discharge to care home after rehabilitation | Dipankar Dutta | 2018 | 2014 to 2017 | UK | 2595 patients admitted to Gloucestershire Royal Hospital, with a diagnosis of stroke confirmed by experienced stroke physicians | Quantitative | Acute care | LTC | age, incontinence, dysphagia, severe weakness, pneumonia, urinary tract infection, depression | - |
| Volume matters: Returning home after hip fracture | Gozalo, P. | 2015 | 2000 to 2007 | USA | Community-dwelling fee-for-service Medi-care beneficiaries aged 75 and older admitted to U.S. hospitals for their first hip fracture and discharged to a SNF for postacute care from 2000 to 2007 (N = 512,967) | Quantitative | SNF (CCC) | Home care | SNF volume of hip fracture admissions (number of hip fracture admissions during the 12 months before participant’s fracture), Higher RN staffing levels (initial beneficial effects) | Age |
| What Factors Predict Adverse Discharge Disposition in Patients Older Than 60 Years Undergoing Lower- extremity Surgery? The Adverse Discharge in Older Patients after Lower-extremity Surgery (ADELES) Risk Score | Maximilian S. Schaefer | 2021 | 2005 to 2017 | USA | patients 60 years or older undergoing lower- extremity orthopaedic surgery | Quantitative | Hospital | LTC / CCC | age older than 90 years (10 points), hip or knee surgery, fracture management, dementia, unmarried status, federally provided insurance, and low estimated household income based on ZIP code | - |
| Factors associated with community versus personal care home discharges after inpatient stroke rehabilitation: the need for a pre-admission predictive model | Alexander Wasserman | 2020 | 2008 to 2017 | Canada | 1588 patients discharged from the inpatient stroke rehabilitation ward at Riverview from 1 April 2008–31 March 2017 | Quantitative | CCC | Home care | - | Older age, having a lower functional independence measure score cognitive deficits, lived alone before their stroke, excessive truncal instability limiting Berg balance scale measurability |
| Frailty predicts failure to discharge patients home from a subacute‑care unit: a 3‑year Italian experience | Paolo Mazzola | 2022 | 2017 to 2020 | Italy | All patients consecutively admitted to the SCU of Desio Hospital during the temporal period between October 2017 and February 2020. | Quantitative | CCC | Home care | - | Having a urinary bladder catheter at discharge, being overtly frail (CFS > 8), and low Barthel Index score |
| Non-home discharge after cardiac surgery in Australia and New Zealand: a cross-sectional study | Mahesh Ramanan | 2021 | 2004 to 2019 | Australia and New Zealand | Adult patients who underwent cardiac surgery from the Australia New Zealand Intensive Care Society Adult Patient Database (APD) | Quantitative | Acute care | LTC, CCC | Increasing age, female sex, non-elective surgery, surgery type (CABG+ valve), Acute Physiology, and Chronic Health Evaluation III (APACHE-III) Score, Surgery in a private hospital | - |
| Predictors of inpatient (neuro)rehabilitation after acute care of severe traumatic brain injury: An epidemiological study | Rahel Schumacher | 2016 | 2007 to 2010 | Switzerland | Patients having sustained severe TBI and having been admitted to one of the Swiss trauma centres, were included. | Quantitative | Hospital | CCC | Lower scores on the Glasgow Coma Scale at admission/at 14 days, higher injury severity scores and older age | - |
| VA Staff Perceptions of Barriers and Facilitators to Home-and Community-Based Placement Post–Hospital Discharge | Edward Alan Miller | 2019 | May 18, 2012, and December 6, 2012 | USA | 35 semi-structured interviews with staff from 12 VA medical centres from around the country | Qualitative | Acute care | Home care | - | High care needs exceeding what can be provided at home, Unpaid, informal care is critical for HCBS to work, Caregiver burden and stress, Homelessness can complicate/preclude HCBS placement, Linking Veterans to non-VA services and supports, Challenges linking Veterans to non-VA services and supports, Insufficient availability of VA providers, Level and type of staffing, Staff unwillingness to enter certain neighbourhoods due to crime and safety concern, Budgetary pressures impact HCBS availability, Variation in decisions affecting the number of service hours and Veterans served, Those living outside the home-based primary care catchment area lack access to these services |
| Prediction of post-acute care demand in medical and neurological inpatients: diagnostic assessment of the post-acute discharge score – a prospective cohort study | Antoinette Conca | 2018 | February to October 2013 | Switzerland | 1896 consecutive medical and neurological patients admitted to the KSA | Quantitative | Acute care | LTC, CCC | Post-acute care discharge tool on day 1 and day 3 of the hospital stay |  |
| Return to community living and mortality after moving to a LTC facility: A nationally representative cohort study | Kenneth Lam | 2022 | 2011 to 2018 | USA | 739 adults over age 65 who had moved from community living into an LTCF between 2011 and 2018 | Quantitative | LTC | Home care | - | Older age, dementia, and previously living alone |
| Predicting Discharge to Institutional LTC AfterStroke: A Systematic Review and Metaanalysis | Jennifer K. Burton | 2018 | From Inception to 2017 | Multiple countries | 18 studies (n=32,139 participants) (Adults hospitalized for stroke who were newly admitted directly to long-term institutional care at the time of hospital discharge) | Literature Review | Acute care | LTC | Older age, greater stroke severity, comorbidities | - |
| How Do Race and Hispanic Ethnicity Affect Nursing Home Admission? Evidence From the Health and Retirement Study | Mieke Beth Thomeer | 2015 | 1998 to 2010 | USA | 18,952 nursing home admission for non-Hispanic whites, non-Hispanic Blacks, and Hispanics from 1998 to 2010 in the Health and Retirement Study | Quantitative | Different locations, its mostly about the race | LTC | being on Medicaid, having an informal caregiver, non-Hispanic whites, older age, ADL, I-ADL, mobility difficulties, cognitive impairments, previous hospitalization, never being married (only for non-Hispanic whites and Hispanics, not for non-Hispanic Blacks!), being widowed or divorced, relying on caregiver, being female (non-Hispanic whites and Hispanics) | Hispanics (this is the lowest rate of admission even compared to Blacks), non-Hispanic Blacks, homeownership, higher income, more living children, living with a child, having future help available if needed (only among Hispanics), Less than high school education, being female (non-Hispanic Blacks) |
| Previous in-home physiotherapy prevents institutionalization after short-term hospitalization in community-dwelling older dependent people | A. Sebban | 2020 | January to December 2016 | France | Community-dwelling older dependent people aged 60 and over, having a natural caregiver, and hospitalized in the acute care unit of the State Geriatric Center. | Quantitative | Acute care | LTC | length of stay in the acute care unit, disruptive behavioural and psychological symptoms of dementia, moderately severe-to-severe cognitive impairment, caregiver burden, living alone with a close or a remote caregiver | In-home physiotherapy |
| Development of a Point System to Predict Discharge to Home for Acute Stroke Patients | Kosei Kubo | 2020 | 2005 to 2015 | Japan | Acute stroke patients were admitted within 3 days of stroke onset and from home, excluding those with incomplete data or who died in the hospital. | Quantitative | Acute care | Home care | Higher Barthel Index on admission, lower modified Rankin Scale, Lower National institute of Health Stroke Scale, stroke type (cerebral infarction or subarachnoid haemorrhage) | Older age, higher stroke severity (NIHSS score), higher disability (mRS score), presence of paralysis |
| Socioeconomic Inequality in the Use of LTC among European Older Adults: An Empirical Approach Using the SHARE Survey | Javier Lera | 2021 | 2017 | Ten European countries | Older adults from ten European countries. | Quantitative | The study focuses on LTC utilization without a specific transition pathway detailed in the context of the settings transitioned from. So its general. | LTC | Age, being single, being female, Higher education level, Number of descendants, Living in an urban area, Number of limitations in activities of daily living (ADLs), Number of chronic diseases (NCD). | At least good self-assessed health status (SAGHS). greater number of household members, |
| Facilitators and Inhibitors in Hospital-to-Home Transitional Care for Elderly Patients with Chronic Diseases: A Meta-Synthesis of Qualitative Studies | Mengjie Sun | 2023 | No date restriction | Multiple countries | Older adults with chronic diseases transitioning from hospital to home, including perspectives from caregivers and healthcare providers. | Literature review (Qual) | Acute care | Home care | Positive personal traits (e.g., motivation, initiative, cooperation), Personal coping strategies (e.g., creating schedules, adapting the home environment, self-care skills), Strong relationships with caregivers, Active support and advocacy from family caregivers, Positive relationships with healthcare providers, Effective care coordination (e.g., transition nurses, multidisciplinary teams), Well-developed electronic systems for information sharing | Negative perceptions of transition, Gaps in knowledge and self-care skills, Indifferent tone and attitude from healthcare providers, Physical and mental symptoms and financial burden of caregivers (PC), fragmented communication and lack of standardized processes, Human resource limitations (e.g., inadequate staffing, insufficient knowledge, and skills), Organizational barriers (e.g., time and workload constraints), E-health literacy, Patients’ residential distance |
| Understanding Pathways into Care homes using Data (UnPiCD study): a retrospective cohort study using national linked health and social care data | Jennifer Burton | 2022 | 2013 to 2016 | UK (Scotland) | Older Adults moving into care homes in Scotland, between April 1, 2013, and March 31, 2016. | Quantitative | Acute care | LTC | Older age, female sex, High frailty, Hospital discharge with a diagnosis of fracture, Hospital discharge with a diagnosis of stroke, Discharge from inpatient psychiatry, a higher number of hospital admissions in the six months before moving into care home, Any diagnosis of dementia, or a hospital discharge with falls, functional dependency | - |
| Factors associated with entry to residential care in frail older inpatients | Karen Misquitta | 2023 | 2007 to 2018 | Australia (Queensland) | Frail older inpatients aged over 60 years, admitted from independent living and referred for geriatric consultation in 27 hospitals in Queensland. | Quantitative | Acute care | LTC | Higher frailty index (each 0.1 increment in FI increased the risk by 54%), Presence of behavioural and psychological symptoms of dementia (BPSD), Faecal incontinence | Being married or in a de facto relationship, living with others |
| Key Care Provision Aspects That Affect Care Transition in the LTC Systems: Preliminary Review Findings | Estera Wieczorek | 2022 | 2005 to 2019 | Multiple countries | Older adults aged 60 and above in LTC systems | Literature review | Various care settings | LTC | Direction is not the focus. It wants to represent the overall aspects that affect transitions: Coordination of resources (e.g., nurse-led programs, medication reconciliation) Communication among involved professional groups  Transfer of information and care responsibility of the patient Training and education of staff Education and involvement of the patient and family Use of e-health technologies Social care support coordination of resources provider payment mechanism (reimbursement) Rewards Penalties | |
| Predicting the Need for Supportive Services After Discharge from Hospital: A Systematic Review | Daniel M. Kobewka | 2020 | 2017 | Multiple countries | Patients admitted non-electively to medical wards and discharged from hospital | Literature review | Acute care | LTC, CCC | Age, Impaired physical function, Disabilities in performing activities of daily living (ADLs), Absence of an informal caregiver, Frailty, Stroke, Disabilities in instrumental activities of daily living (IADLs), Receiving supportive services prior to hospital admission | - |
| Extension and Validation of the Self-care Index to Predict Transfer to a Post-acute Care Institution in Internal Medicine Patients | Antoinette Conca | 2022 | 2013 | Switzerland | 1372 adult internal medicine in-patients admitted from home | Quantitative | Acute care | LTC, CCC | Higher age, Female gender, Lower overall self-care abilities (lower SPI total score), Lower ability to acquire knowledge, Higher dependency in activities of daily living | - |
| LTC transitions during a global pandemic: Planning and decision-making of residents, care partners, and health professionals in Ontario, Canada | Sarah Carbone | 2023 | 2021 to 2022 | Canada | 32 participants (3 residents, 18 care partners, 11 health professionals) | Qualitative | LTC | Home care | Fear of COVID-19 infection in LTC Desire to avoid isolation policies in LTC Strong care partner support and resources Belief in personal responsibility for care Negative media portrayals of LTC during the pandemic Perception of better quality of life in community Availability of appropriate home environment | Complex health and support needs of residents Limited availability of home care services Lack of access to primary care providers in community Potential for caregiver burnout Insufficient resources (financial, time, equipment) Positive perceptions of care quality in LTC Concerns about disrupting resident’s routine or environment |
| Predictors for living at home after geriatric inpatient rehabilitation: a prospective cohort study | Jan Kool | 2017 | 2014 | Switzerland | All patients aged 65 years or more who were referred for geriatric inpatient rehabilitation were eligible to enter the study | Quantitative | CCC | Home care | Better mobility at discharge, lower multimorbidity, better cognition, and not living alone. | - |
| Factors influencing home discharge of hospitalized oldest-old patients (≥ 90 years): A retrospective quantitative case-control study | Toru Takekawa | 2024 | Jan 1 to Dec 31 2020 | Tokyo, Japan | 90 patients aged ≥90 (mean ≈91 yrs) | Quantitative | Acute care | Home care | Living with someone, partial or no assistance with eating, higher lymphocyte count, better mobility scores | Living alone, full assistance for eating, low lymphocyte count, low mobility scores |
| Skilled Nursing Facility Rehabilitation Intensity and Successful Discharge in Persons with Dementia | Thomas Bayer | 2024 | Jan 2011 to Jun 2019 | USA | 8 255 veterans ≥ 65 y with dementia | Quantitative | SNF (CCC) | Home care | Receiving ≥5.3 h/week of skilled therapy; each additional hour of therapy increases likelihood of home discharge | Higher comorbidity counts, greater ADL dependency, cognitive impairment |
| Low phase angle: A predictor of functional status and discharge disposition in acute stroke older patients | Kota Amakasu | 2024 | Oct 2021 to Dec 2022 | Japan | 205 acute stroke patients aged ≥ 65 starting rehab within 1 week | Quantitative | Acute care | Home care | Good trunk control (Trunk Control Test ≥40), mild stroke severity (NIHSS 0-5), normal phase angle | Low phase angle,, poor trunk control |
| Factors Affecting Home Discharge of Older Adults with Cervical Spinal Cord Injury in Japan Regional Population | Sota Sasaki | 2023 | 2005 to 2020 | Japan | 219 patients (mean age ≈74 yrs) | Quantitative | Acute care | Home care | Age < 75 y, Living with someone, Lower neurological level (C5-8), Incomplete injury (AIS B-D), Higher functional independence at admission | Age ≥ 75 y, Living alone, High neurological level (C1-4), Complete injury AIS A |
| Preoperative Risk Factors for Discharge to Facility After Surgery in Geriatric Patients | Danielle Abbitt | 2024 | Jan 2018 to Dec 2022 | USA | 432 patients aged ≥75 (mean ≈79 yrs) | Quantitative | Acute care | Home care | No falls in prior 6 mo, No mobility-aid use, Pre-illness independent functional status, Living with others | ≥ 1 fall in 6 mo, Use of cane/walker/wheelchair, pre‑illness functional dependence, Living alone, orthopedic surgery |
| Predictors of 15-year transitions across living and care settings in a population of Swedish older adults | Susanna Gentili | 2025 | 2001 to Jan 2017 | Sweden | 3,021 adults aged ≥60 (mean ≈74.7 yrs) | Quantitative | Acute care | Home care | Older age and female sex increased likelihood of home care after hospital/post‑acute, higher education and being unpartnered increased odds of hospitalisation and hospital‑to‑home with social services | Cognitive impairment and ADL/IADL disability reduced chance of returning home and increased hazard of nursing home placement, slower gait speed raised risk of hospitalisation and nursing‑home discharge |
| Multistate Competing Risk Analysis of Transition Back to the Community Among LTC-Destined Patients: A Brief Report | Bonaventure Egbujie | 2023 | Jan 2020 to Jun 2023 | Canada | 111 patients enrolled in Harbour Light transitional unit aged > 65 | Quantitative | Acute care | Home care | Female sex, Usual home available | Higher cognitive impairment (CPS), Post‑Acute Delayed Discharge Risk score ≥1, greater ADL hierarchy, heart failure, falls |
| COVID-19 Pandemic and Racial and Ethnic Disparities in Long-Term NH Stay or Death Following Hospital Discharge | Laurent G. Glance | 2025 | Jan 2016 to Jun 2021 | USA | 2,964,517 community-dwelling adults ≥ 65 y hospitalized with sepsis | Quantitative | Acute care | LTC | Frailty-related factors (malnutrition, fecal incontinence, housing instability, neurologic disorders, psychoses, severe kidney failure, congestive heart failure, stroke, acute respiratory failure), being Black, pandemic period increased risk | Being Asian/Pacific Islander, Hispanic or American Indian/Alaska Native, pre-pandemic trend of declining LTC stay or death |
| Post-Acute Care Transitions and Outcomes Among Medicare Beneficiaries with Dementia: Associations with Race/Ethnicity and Dual Status | Helena Temkin-Greener | 2023 | 2017 | USA | 619,262 Medicare FFS beneficiaries with dementia aged > 65 | Quantitative | Acute care | CCC | Older age, surgical admission, prior nursing home use | Dual Medicare-Medicaid eligibility and longer time since dementia diagnosis reduced likelihood of institutional discharge |
| Caregiver preparedness is associated with desire to seek long-term care admission of hospitalized persons with dementia | Ashley Kuzmik | 2023 | 2018 to 2021 | USA | 424 patient-caregiver dyads, patients aged > 65 | Quantitative | Acute care | LTC | Lower caregiver preparedness, caregiver cohabitation, greater patient delirium severity increased desire for LTC | White caregiver ethnicity reduced desire for LTC, higher caregiver preparedness mitigated desire to institutionalize |
| Impact of vision impairment on discharge destination for patients with hip fracture | Jacarri Tollette | 2024 | 2015 | USA | 10,336 Medicare inpatients ≥ 65 with hip fracture | Quantitative | Acute care | LTC, CCC | Male sex, Black race, systemic complications and late postoperative discharge increased likelihood of LTCF discharge versus home | Vision impairment was not a significant predictor; protective factors for home discharge not strongly identified |
| Post-acute care transitions during COVID-19: racial, ethnic, and socioeconomic differences in older adults with ADRD | Ming-Ting Yang | 2024 | 2019 to 2021 | USA | 830,656 Medicare FFS ≥ 65 with ADRD hospitalized | Quantitative | Acute care | SNF (CCC) | Dual Medicare-Medicaid eligibility increased likelihood of SNF discharge and modestly increased HHA discharge pre-pandemic | Non-Latinx Black or Latinx ethnicity reduced likelihood of SNF discharge, dual eligibility reduced HHA discharge during the pandemic |
| A comparison of acute ischemic stroke patients discharged to inpatient rehabilitation vs skilled nursing facility | Priyadarshini Pattath | 2023 | 2016 to 2019 | USA | 130,988 acute ischemic stroke survivors (mean age ≈74 yrs) | Quantitative | Acute care | SNF (CCC) | Older age (≥85), non-Hispanic Black or Hispanic ethnicity, Medicaid/Medicare insurance (vs private), lower ambulation ability, prior stroke, diabetes, myocardial infarction/coronary artery disease increased likelihood of SNF discharge | Male sex, slight disability (modified Rankin Score 2), admission to large hospitals and hospitals with stroke units decrease likelihood of SNF discharge |
| Referral to geriatric rehabilitation in the Netherlands, an exploratory study of patient characteristics | Aafke J. de Groot | 2025 | Jan 15 to May 15 2019 | The Netherlands | 87 patients (mean age ≈76.3 yrs) | Quantitative | Acute care | CCC | Complications, cognitive symptoms, high multi-domain vulnerability | Independent baseline mobility, absence of complications, fewer cognitive symptoms, less multi-domain vulnerability |
| In the footstep of the old patient from hospital to home: A qualitative field observation study | Sanne Have Beck et al. | 2024 | Not Specified | Denmark | 10 older hospitalized patients (geriatric department) | Qualitative | Acute care | Home | Presence and active involvement of relatives in discharge interactions, Adaptation of home environment into a “workplace” supporting daily activities | Limited involvement in discharge planning leading to loss of autonomy, Fragmented communication across sectors causing helplessness and fear of burdening family |
| Facilitating the transition from hospital to home after hip fracture surgery: a qualitative study from the HIP HELPER trial | A Welsh | 2024 | Nov 2021 to Mar 2022 | UK | 10 patient-carer dyads (age > 60) + 8 health professionals | Qualitative | Acute care | Home | Reassurance, collaborative planning, individualized support and coordinated discharge planning | Ineffective communication among providers/patients, Disjointed care systems delaying services, neglect of holistic needs, |
| The DEPARTS Score: A Novel Tool for Predicting Discharge Disposition in Geriatric Trauma Patients | Eric O. Yeates | 2023 | 2017 | USA | 132,956 geriatric trauma patients (> 65 y) from the 2017 TQIP cohort (split equally into derivation & validation) | Quantitative | Acute care | LTC/CCC | Higher number of comorbidities, fall mechanism, spinal cord injury, long bone fracture, major surgery | Fewer comorbidities and absence of the injuries |

## **S3. Detailed factors influencing post-acute care (PAC) transitions**

### **S3.1. Factors influencing transitions from acute care to LTC**

**Socio-demographic characteristics.** Age was the most frequently cited demographic factor linked to LTC placement in quantitative studies (n=21) [1–21], with further support from qualitative studies and reviews (n=2) [22,23]. Gender also emerged as a significant determinant. Female gender was associated with a greater likelihood of LTC transitions in eight quantitative studies (n=8) [1,3,8,10,13,21,24,25] and three systematic reviews (n=3) [20,22,26]. However, one quantitative study reported that males are more likely to transition to LTC (n=1) [27]. Living arrangements were also critical; individuals living alone (n=7) [16,17,19,28–31] were more likely to transition to LTC, with mixed methods research (n=1) [32] emphasizing social isolation as a contributing factor. Similarly, marital status played a role, with studies (n=6) [12,16,19,21,22,24] linking unmarried, widowed, or divorced individuals to a higher likelihood of LTC transitions. In contrast, marriage or de facto relationships were identified as protective factors. This association was also confirmed in a systematic review (n=1) [26].

Race and ethnicity showed varied impacts. Black ethnicities were associated with an increased likelihood of LTC placement (n=3) [8,27,33], whereas Hispanic ethnicity (n=2) [16,33] and Asian/Pacific Islander or American-Indian/Alaska-Native backgrounds (n=1) [33] was linked to a reduced likelihood. Finally, lower socioeconomic status and household income (n=3) [5,12,33], were associated with increased LTC transitions, while higher income (n=1) [16] was a protective factor.

**Caregiver support****.** In quantitative research, caregiver distress and an inability to provide informal care were associated with an increased likelihood of LTC placement (n=3) [28,34,35]. Conversely, protective factors against LTC transitions included strong family caregiving networks in quantitative research (n=1) [16] and reliable family caregivers in qualitative studies (n=1) [32].

**Health conditions.** Comorbidities were consistently identified as increasing the likelihood of LTC admission in quantitative studies (n=5) [1,8,18,24,33] and one systematic review [15]. In terms of cognitive health, dementia (n=11) [6,7,12,17,18,20,24,26,36–38] stands out as a major factor, increasing the probability of LTC transitions. Similarly, general cognitive impairment was found to increase LTC admissions (n=3) [16,28,30], as were certain dementia subtypes (n=2) [3,19]. Mental health conditions, including behavioral challenges (n=5) [19,22,29,30,32], depression (n=2) [11,17], various psychiatric conditions (n=1) [20], and refusal of care (n=1) [32] also elevate the likelihood of LTC transitions.

Functional and physical health factors considerably shape these transitions. Functional impairments were demonstrated to necessitate LTC in quantitative studies (n=6) [18–20,24,29,37] and systematic reviews (n=4) [22,26,34,38]. Mobility challenges are also positively associated with LTC transitions (n=3) [2,6,16], as is a high level of dependence on activities of daily living (ADL), as supported by quantitative studies (n=6) [4,6,16,25,28,39] and systematic reviews (n=1) [34]. Additionally, higher frailty levels were identified as determinants of the need for LTC in quantitative studies (n=9) [18–20,33,37,39–42] and one systematic review [34]. Falls are another risk factor for these transitions, as seen in quantitative studies (n=5) [3,20,24,28,39] and a systematic review [22].

Beyond these, specific health conditions were documented as increasing LTC placement in quantitative studies. These include neurological conditions, including Parkinson’s disease and stroke (n=2) [17,20], urological conditions (n=2) [3,18], musculoskeletal conditions (n=2) [3,24], cardiovascular conditions (n=1) [7], respiratory conditions (n=1) [11], incontinence (n=3) [3,11,19], and medical instability (n=1) [28]. Moreover, cancer was identified as increasing the likelihood of LTC placement in a systematic review [22]. Lastly, poor nutritional status was reported to increase this likelihood in a systematic review (n=1) [26] and a quantitative study (n=1) [9].

**Healthcare systems.** Specific clinical procedures were particularly important in determining LTC placement. Major surgeries, including total knee arthroplasty (TKA) (n=2) [5,12], cardiac procedures, such as transcatheter aortic valve replacement (TAVR) and coronary artery bypass graft (CABG) (n=2) [10,13], and gynecological surgeries (n=1) [9] were all associated with an increased likelihood of LTC transition. Additional factors heightening LTC placement risk included non-elective surgeries (n=1) [13], general anesthesia (n=1) [6], respiratory treatments (n=1) [4], tube insertions in hospital (n=1) [22], and multiple procedures during a single hospitalization (n=1) [1]. Conversely, pre-hospitalization physiotherapy (n=1) [30] decreased the likelihood of LTC transition.

Operational efficiency within hospitals also played a role. Higher patient-to-nurse ratios (7:1) were linked with increased LTC transitions (n=1) [4]. Several institutional practices were identified as barriers to effective transition processes. This includes the dominance of healthcare team decision-making with limited incorporation of patient and family preferences (n=1) [43], inadequate staff education (n=1) [44], delayed communication between hospitals and LTC in rural areas (n=1) [45], and poor inter-provider communication (n=2) [44,46]. Notably, electronic health records (EHR) and information transfer mechanisms were identified in systematic reviews (n=1) [46] and mixed methods studies (n=1) [44] as critical tools affecting transition processes.

The availability of LTC beds was another determinant, with limited bed availability prolonging hospital stays in quantitative (n=1) [38], qualitative (n=1) [45], and mixed methods research (n=1) [44]. Resource availability and coordination were also emphasized in systematic reviews (n=3) [38,43,46] as essential components in effective transition planning.

### **S3.2. Factors influencing transitions from acute care to CCC**

**Socio-demographic characteristics.** In quantitative studies, age was the most frequently cited factor associated with an increased likelihood of CCC placement (n=9) [2,4,6,9,10,13,14,47,48]. This association was also supported by a systematic review (n=1) [34]. Female gender (n=5) [8,10,13,25,48], living alone (n=2) [29,47], unmarried status (n=1) [12], and low estimated household income (n=1) [12] were further associated with a higher likelihood of CCC placement. Race and ethnicity showed mixed effects. Asian or African American race (n=1) [47], non-Latinx Black or Latinx ethnicity (n=1) [49] appeared to decrease the likelihood of CCC placement, while Hispanic and non-Hispanic Black ethnicities increased it (n=1) [48].

**Caregiver support.** This aspect was minimally documented in CCC transitions. Specifically, one systematic review identified the absence of an informal caregiver as increasing the likelihood of CCC placement (n=1) [34], while one report highlighted the importance of discussing needs with family members actively involved in the assessment process to facilitate these transitions (n=1) [50].

**Health conditions.** Comorbidities were identified as increasing the likelihood of CCC transitions in quantitative studies (n=3) [8,51,52]. Regarding cognitive health, an intriguing pattern emerged: One study found that the absence of dementia increased the likelihood of CCC placement (n=1) [6], two studies reported that dementia and cognitive symptoms increased the likelihood of CCC placement (n=2) [12,53], and another study reported that a longer time since dementia diagnosis reduced the likelihood of CCC placement (n=1) [54].

Functional impairments were extensively highlighted as necessitating CCC transitions in quantitative studies (n=4) [2,6,25,47]. Within this category, mobility challenges (n=4) [2,6,47,48], increased ADL dependence (n=5) [4,6,25,47,53], and high frailty levels (n=4) [34,40–42] all raised the likelihood of CCC placement. In contrast, mental health conditions were less frequently documented, with only behavioral issues (n=1) [29] specifically identified as contributing to CCC placement.

Lastly, specific health conditions influenced CCC transitions. Cardiovascular issues, such as congestive heart failure, arrhythmia, and coronary artery disease, were associated with decreased transitions to CCC (n=1) [47]. On the other hand, chronic liver disease (n=1) [10], spinal-cord injury (n=1) [52], long-bone fracture (n=1) [52], major surgery (n=1) [52], diabetes (n=1) [48], myocardial infraction (n=1) [48], prior stroke (n=1) [48], as well as high multi-domain vulnerability (n=1) [53] and other complications, all increased these transitions.

**Healthcare system.** Specific surgical procedures were associated with an increased likelihood of CCC transitions, including intramedullary implant procedures (n=1) [6], abdominal hysterectomy (n=1) [9], non-transfemoral cardiac approaches(n=1) [10], and trauma-related major surgery (n=1) [52]. Other medical treatments linked to increased CCC placement included electrocardiography and respiratory treatments (n=1) [4] and the use of general anesthesia (n=1) [6].

Hospital operational efficiency was minimally investigated in relation to CCC transitions, with a patient-to-nurse ratio of 7:1 (n=1) [4] identified as a factor increasing these transitions. Finally, access to services was examined in systematic reviews (n=2) [34,50], which underscored the impact of pre-admission supportive services and overall service availability on CCC transitions.

### **S3.3. Factors influencing transitions from acute care to home care**

**Socio-demographic characteristics.** In quantitative studies, older age (n=6) [5,55–59], was associated with a decreased likelihood of home care placement. Gender also played a role, with females being more likely than males to transition to home care in four studies (n=4) [56,57,60,61], but less likely than males in one study (n=1) [5]. Race and ethnicity influenced transitions as well, with Hispanic ethnicity and non-Black racial/ethnic groups linked to higher rates of transition to home care (n=2) [5,57]. Living alone was found to reduce the likelihood of home care placement (n=4) [31,59,62,63]. Qualitative studies highlighted further barriers, including language difficulties among ethnic minorities (n=1) [64] and socioeconomic constraints such as unaffordable care (n=1) [64] and homelessness (n=1) [65].

**Caregiver support.** Quantitative evidence indicated that the availability of caregiver support facilitated transitions to home care (n=1) [28]. Qualitative studies further emphasized the critical role of caregiver involvement. In particular, active participation of relatives and collaborative discharge planning (n=1) [66] were found to promote successful transitions, while excluding carers from these processes led to stress and uncertainty (n=2) [66,67]. Qualitative findings also highlighted several barriers, including caregiver unpreparedness (n=1), burnout and stress (n=1), insufficient informal support (n=1), and variations in family capacity to provide care (n=1) [65,68,69]. Systematic reviews reinforced these observations, reporting the negative impact of overwhelmed and unprepared caregivers on transitions (n=3) [38,70,71].

**Health conditions.** An advanced comorbidity burden was associated with reduced likelihood of transition to home care, particularly in cases of metastatic cancer (n=1) [56] and heart failure (n=1) [61]. Regarding cognitive health, higher Mini-Mental State Examination (MMSE) scores (n=1) [31] and better cognitive performance (n=1) [72] increased the probability of home transitions, whereas cognitive impairment decreased it (n=2) [60,61]. In the context of mental health, depression (n=1) [70] and behavioral issues, especially aggressiveness (n=1) [31], were noted to hinder home care transitions.

Functional capacity was also a key determinant. Higher Barthel Index scores and lower disability levels (n=1) [58], superior mobility performance evidenced through 100-meter walk achievement (n=1) [73], higher functional and mobility scores (n=2) [59,72], and enhanced ADL capability measured using the Nishimura-style scale (n=1) [31] all increased the likelihood of home transitions. These findings were further supported by studies identifying better trunk-control performance (n=1) [74], and pre-illness functional status without the use of mobility aids (n=1) [63] as transition facilitators. Nutritional status also played a role. Better nutritional indicators, including higher body mass index (BMI) (n=1) [56] and superior geriatric nutritional risk index (GNRI) scores (n=1) [73], facilitated home transitions, whereas malnutrition (n=1) [56] decreased its likelihood.

Specific health conditions further influenced transitions. A higher likelihood of home care transition was reported among individuals without musculoskeletal conditions, such as cervical spine, pelvic, or hip fractures (n=1) [55], those with lower stroke severity (n=1) [58], incomplete cervical spinal cord injuries (n=1) [59], lower neurological-level lesions (n=1) [59], and no recent history of falls (n=1) [63]. In contrast, prior traumatic brain injury (n=1) [57], pressure ulcers (n=1) [56], and pleural effusion (n=1) [56] negatively influenced these transitions.

**Healthcare system.** Clinical interventions significantly influenced transitions from acute care to home care. For instance, total knee arthroplasty (n=1) [5] and complex medical treatments such as respiratory support, vasopressor administration, and oxygen therapy (n=1) [56] were associated with a decreased likelihood of home care transitions. Conversely, pre-hospitalization physiotherapy was found to increase the likelihood of home care placement [30].

Regarding hospital operational efficiency, integrated care systems incorporating Lean Six Sigma principles and person-centered approaches (n=1) [75], along with clearly defined roles among healthcare providers (n=1) [75], were found to facilitate home transitions. Qualitative studies further emphasized the importance of collaborative planning and early adaptation of the home environment to support daily activities (n=2) [66,67]. However, several barriers were also identified. High staff workload and poor inter-professional communication (n=4) [66,67,70,76], unclear role definitions (n=1) [76], and difficulty incorporating family preferences into healthcare decision-making (n=5) [66–69,75], were all found to hinder transitions*.* Additional challenges included disjointed care systems leading to service delays (n=1) [67], care demands exceeding available resources (n=1) [65], lack of standardized processes (n=1) [70], and shortened hospital stay for complex patients (n=1) [71].

Information technology was also reported as a key enabler of successful transitions. Health information exchange (n=1) [77], shared information systems (n=1) [76], and telehealth services (n=1) [78] increased the likelihood of home care placement. Systematic reviews further emphasized the importance of robust electronic systems for information sharing (n=1) [70], as well as standardized medication reconciliation through EHR (n=1) [71] in transitions. On the other hand, delays in accessing timely information (n=1) [68] were highlighted as an impediment to these transitions.

Access to post-discharge services posed continuing challenges. Barriers included insufficient home care services, especially for overnight support (n=1) [68], geographic disparities in service availability (n=1) [65], and inequities in local healthcare resources (n=1) [64]. Systematic reviews reinforced these findings, noting insufficient support for older adults with complex health needs during hospital-to-home transitions (n=1) [38], as well as limitations in human resources (n=1) [70].

## **S4. Frequency of influential factors in PAC transitions**

Table S3 presents the complete list of factors influencing the transition of older adults from acute care to LTC, CCC, and home care and their frequency.

**Table S3**. Full list of PAC transitions’ influential factors

| Factors associated with older adults’ post-acute care transitions | | Acute care to LTC | | | | | Acute care to CCC | | | | | Acute care to home care | | | | |
| --- | --- | --- | --- | --- | --- | --- | --- | --- | --- | --- | --- | --- | --- | --- | --- | --- |
|  |  | Quantitative | | Qualitative | | | Quantitative | | Qualitative | | | Quantitative | | Qualitative | | |
|  |  | Positive | Negative | Positive | Negative | Not Specified | Positive | Negative | Positive | Negative | Not Specified | Positive | Negative | Positive | Negative | Not Specified |
| Socio-demographic characteristics | | | | | | | | | | | | | | | | |
|  | Age | 21 | - | 2 | - | - | 9 | - | - | 1 | - | - | 6 | - | - | - |
|  | Gender (Female) | 8 | 1 | 3 | - | - | 5 | - | - | - | - | 4 | 1 | - | - | - |
|  | Higher income | - | 3 | - | - | - | - | - | - | - | - | - | - | - | - | - |
|  | Homeownership | - | 1 | - | - | - | - | - | - | - | - | - | - | - | - | - |
|  | Homelessness | - | - | - | - | - | - | - | - | - | - | - | - | - | 1 | - |
|  | Less than high school education | - | 1 | - | - | - | - | - | - | - | - | - | - | - | - | - |
|  | Language barriers | - | - | - | - | - | - | - | - | - | - | - | - | - | 1 | - |
|  | Living alone | 7 | - | 1 | - | - | 2 | - | - | - | - | - | 4 | - | - | - |
|  | Lower income | 2 | - | - | - | - | - | - | - | - | - | - | - | - | - | - |
|  | Low to middle socioeconomic status | 1 | - | - | - | - | 1 | - | - | - | - | - | - | - | 1 | - |
|  | Not married | 6 | - | 1 | - | - | 1 | - | - | - | - | - | - | - | - | - |
|  | Race | 3 Black, 1 non-Hispanic | 1 Asian/Pacific Islander, 2 Hispanic | - | - | - | - | 1 Asian, 1 African American | - | - | - | 1 Hispanic, 1 non-Black | - | - | - | - |
| Caregiver support | | | | | | | | | | | | | | | | |
|  | Caregiver distress | 3 | - | - | - | - | - | - | - | - | - | - | - | - | 2 | - |
|  | Caregiver capacity/experience | - | - | - | - | - | - | - | - | - | - | - | - | 1 | - | - |
|  | Family support networks | 1 | - | 1 | - | - | - | - | 1 | - | - | 1 | - | 1 | - | - |
|  | Caregiver unpreparedness | - | - | - | - | - | - | - | 1 | - | - | - | - | - | 4 | - |
| Health conditions | | | | | | | | | | | | | | | | |
|  | 100 m walk achievement | - | - | - | - | - | - | - | - | - | - | 1 | - | - | - | - |
|  | Admission Glasgow Coma Scale | - | 1 | - | - | - | - | - | - | - | - | - | - | - | - | - |
|  | BMI | 1 (high BMI) | - | - | - | - | 1 (high BMI) | - | - | - | - | 1 (low BMI) | - | - | - | - |
|  | Cancer diagnosis | 1 | - | - | - | - | - | - | - | - | - | - | 1 | - | - | - |
|  | Cardiovascular | 1 | - | - | - | - | 2 | - | - | - | - | - | - | - | - | - |
|  | Chronic liver disease | - | - | - | - | - | - | - | - | - | - | - | 1 | - | - | - |
|  | Cognitive impairment | 3 | - | - | - | - | 2 | - | - | - | - | 2 | - | - | - | - |
|  | Comorbidities | 5 | - | 1 | - | - | 3 | - | - | - | - | - | 2 | - | - | - |
|  | Dementia | 11 | - | - | - | - | - | 2 | - | - | - | - | 2 | - | - | - |
|  | Dependence in ADL | 6 | - | 2 | - | - | 5 | - | - | - | - | - | 1 | - | - | - |
|  | Depression | 2 | - | - | - | - | - | - | - | - | - | - | - | - | - | - |
|  | Dyslipidemia | 1 | - | - | - | - | 1 | - | - | - | - | - | - | - | - | - |
|  | Dysphagia | 1 | - | - | - | - | - | - | - | - | - | - | - | - | - | - |
|  | Functional impairment | 6 | - | 3 | - | - | 4 | - | - | - | - | - | - | - | - | - |
|  | Falls | 5 | - | 1 | - | - | - | - | - | - | - | - | - | - | - | - |
|  | Frailty | 9 | - | 1 | - | - | 4 | - | - | - | - | - | 1 | - | - | - |
|  | Goal-attainment | - | - | - | - | - | - | - | - | - | - | 1 | - | - | - | - |
|  | Higher injury severity score | - | 1 | - | - | - | - | 1 | - | - | - | - | - | - | - | - |
|  | Have markers of increased Traumatic Brain Injury severity | - | - | - | - | - | 1 | - | - | - | - | - | - | - | - | - |
|  | High Mini-Mental State Examination and Nishimura-style senile ADL scores | - | - | - | - | - | - | - | - | - | - | 1 | - | - | - | - |
|  | High scores in post-acute care discharge tool | 1 | - | - | - | - | - | - | - | - | - | - | - | - | - | - |
|  | Higher Cognitive scores | - | - | - | - | - | - | - | - | - | - | 1 | - | - | - | - |
|  | Higher functional independence | - | - | - | - | - | - | - | - | - | - | 2 | - | - | - | - |
|  | Hip fracture | 2 | - | - | - | - | - | - | - | - | - | - | - | - | - | - |
|  | History of CABG | 1 | - | - | - | - | 1 | - | - | - | - | - | - | - | - | - |
|  | History of pacemaker | 1 | - | - | - | - | 1 | - | - | - | - | - | - | - | - | - |
|  | Impaired bathing | 1 | - | - | - | - | - | - | - | - | - | - | - | - | - | - |
|  | Impaired consciousness level on the Japan coma scale | - | - | - | - | - | - | - | - | - | - | - | 1 | - | - | - |
|  | Impaired mobility | 3 | - | - | - | - | 3 | - | - | - | - | - | 1 | - | - | - |
|  | Incontinence | 3 | - | - | - | - | - | - | - | - | - | - | - | - | - | - |
|  | Level of obesity | 1 | - | - | - | - | - | - | - | - | - | - | - | - | - | - |
|  | Lower premorbid Katz-ADL | - | - | - | - | - | 1 | - | - | - | - | - | - | - | - | - |
|  | Manifestation of aggressiveness at the time of admission | - | - | - | - | - | - | - | - | - | - | - | 1 | - | - | - |
|  | Medical instability | 1 | - | - | - | - | - | - | - | - | - | - | - | - | - | - |
|  | Mental and behavioral issues | 5 | - | - | - | - | 1 | - | - | - | - | - | - | - | 1 | - |
|  | More pre-injury chronic conditions | 1 | - | - | - | - | - | - | - | - | - | - | - | - | - | - |
|  | Multiple non-head injuries | - | - | - | - | - | 1 | - | - | - | - | - | - | - | - | - |
|  | Musculoskeletal | 2 | - | - | - | - | - | - | - | - | - | - | 1 | - | - | - |
|  | Not being ambulatory before surgery | 1 | - | - | - | - | 1 | - | - | - | - | - | - | - | - | - |
|  | Neurological | 2 | - | - | - | - | 1 | - | - | - | - | - | - | - | - | - |
|  | Nutritional status (The Geriatric Nutritional Risk Index (GNRI) | 1 | - | 1 | - | - | - | - | - | - | - | 2 | 1 | - | - | - |
|  | Parkinson’s disease | 1 | - | - | - | - | - | - | - | - | - | - | - | - | - | - |
|  | Pleural effusion | - | - | - | - | - | - | - | - | - | - | - | 1 | - | - | - |
|  | Pneumonia | 1 | - | - | - | - | - | - | - | - | - | - | - | - | - | - |
|  | Pressure ulcers | - | - | - | - | - | - | - | - | - | - | - | 1 | - | - | - |
|  | Requires physical assistance | 1 | - | - | - | - | - | - | - | - | - | - | - | - | - | - |
|  | Respiratory | 1 | - | - | - | - | - | - | - | - | - | - | - | - | - | - |
|  | Severe or Critical Abbreviated Injury Scale scores | - | - | - | - | - | 1 | - | - | - | - | - | 1 | - | - | - |
|  | Severe weakness | 1 | - | - | - | - | - | - | - | - | - | - | - | - | - | - |
|  | Traumatic Brain Injury | - | - | - | - | - | 1 | - | - | - | - | - | 1 | - | - | - |
|  | The self-care abilities of patients | - | 1 | - | - | - | - | 1 | - | - | - | - | - | - | - | - |
|  | Urinary tract infection | 2 | - | - | - | - | - | - | - | - | - | - | - | - | - | - |
|  | Urological | 2 | - | - | - | - | 1 | - | - | - | - | - | - | - | - | - |
|  | Using several medications | - | - | 1 | - | - |  |  |  |  |  |  |  |  |  |  |
|  | Wandering | 1 | - | - | - | - | - | - | - | - | - | - | - | - | - | - |
| Healthcare systems | | | | | | | | | | | | | | | | |
|  | Abdominal hysterectomy | 1 | - | - | - | - | 1 | - | - | - | - | - | - | - | - | - |
|  | Integrated care systems | - | - | - | - | - | - | - | - | - | - | - | - | 1 | - | - |
|  | Assessment location (hospital) | 2 | - | 1 | - | - | - | - | - | - | - | - | - | - | - | - |
|  | Assessment tools | - | - | - | - | - | 1 | - | - | - | - | - | - | - | - | - |
|  | Cardiac procedures | 2 | - | - | - | - | 1 | - | - | - | - | - | - | - | - | - |
|  | Care coordination | - | - | - | - | - | - | - | - | - | - | - | - | 2 | 1 | - |
|  | Communication systems | - | - | 2 | - | - | - | - | - | - | - | - | - | - | - | - |
|  | Cotreatment by a geriatrician | 1 | - | - | - | - | 1 | - | - | - | - | - | - | - | - | - |
|  | Early physical therapy assessment | - | 1 | - | - | - | - | - | - | - | - | 1 | - | - | - | - |
|  | Elective procedures | 1 | - | - | - | - | 1 | - | - | - | - | - | 1 | - | - | - |
|  | Electrocardiography | 1 | - | - | - | - | 1 | - | - | - | - | - | - | - | - | - |
|  | The emotional cost to relatives involved in decision-making | - | - | 1 | - | - | - | - | - | - | - | - | - | - | - | - |
|  | Facility size | 1 | - | - | - | - | - | - | - | - | - | - | - | - | - | - |
|  | Facilities with higher proportions of patients with histories of trauma or major surgery | - | 1 | - | - | - | - | - | - | - | - | - | - | - | - | - |
|  | Financial pressures and insurance limitations | - | - | 1 | - | 1 | - | - | - | - | - | - | - | 3 | 1 | - |
|  | Financial resources and economic viability of Care at Home | - | - | 1 | - | 1 | - | - | - | - | - | - | - | 3 | - | - |
|  | Fracture management | 1 | - | - | - | - | - | - | - | - | - | - | - | - | - | - |
|  | Fragmented hospital readmissions |  |  |  |  |  |  |  |  |  |  |  | 1 |  |  |  |
|  | Geographic disparities | - | - | - | - | - | - | - | - | - | - | - | - | - | 1 | - |
|  | Gynecological surgeries | 1 | - | - | - | - | 1 | - | - | - | - | - | - | - | - | - |
|  | Geriatric assessment | - | - | 1 | - | - | - | - | - | - | - | - | - | - | - | - |
|  | Telehealth Services | - | - | - | - | - | - | - | - | - | - | - | - | 2 | - | - |
|  | Having a urinary bladder catheter at discharge | - | - | - | - | - | - | - | - | - | - | - | 1 | - | - | - |
|  | Having future help available if needed | - | 1 | - | - | - | - | - | - | - | - | - | - | - | - | - |
|  | Health information exchange | - | - | 2 | - | - | - | - | - | - | - | 1 | - | 3 | - | - |
|  | Hip/knee surgery | 4 | - | - | - | - | 1 | - | - | - | - | - | 1 | - | - | - |
|  | Intensity of services (e.g., number of hours of nursing care or therapy) | - | - | - | - | - | - | - | - | - | - | - | - | 1 | - | - |
|  | Intramedullary Implant | 1 | - | - | - | - | 1 | - | - | - | - | - | - | - | - | - |
|  | Inadequate information transfer to caregivers | - | - | 3 | - | - | - | - | - | - | - | - | - | - | - | - |
|  | Lack of staff with proper education | - | - | 1 | - | - | - | - | - | - | - | - | - | 1 | - | - |
|  | Laparoscopic hysterectomy | 1 | - | - | - | - | 1 | - | - | - | - | - | - | - | - | - |
|  | Laparoscopic Sacro colpopexy | 1 | - | - | - | - | 1 | - | - | - | - | - | - | - | - | - |
|  | Length of stay | 2 | - | - | - | - | - | - | - | - | - | - | 1 | - | - | - |
|  | Lower licensed practical nurse (LPN) to registered nurse (RN) ratios | - | 1 | - | - | - | - | - | - | - | - | - | - | - | - | - |
|  | Non-elective surgery | 1 | - | - | - | - | 1 | - | - | - | - | - | - | - | - | - |
|  | Nursing home availability and infrastructure and funding | - | - | 5 | - | - | - | - | - | - | - | - | - | - | - | - |
|  | Older adults with complex needs are not always identified upon hospital admission or before discharge | - | - | 1 | - | - | - | - | - | - | - | - | - | - | - | - |
|  | Overnight support | - | - | - | - | - | - | - | - | - | - | - | - | - | 1 | - |
|  | Oxygen therapy | - | - | - | - | - | - | - | - | - | - | - | 1 | - | - | - |
|  | Patient education and support | - | - | 1 | - | - | - | - | - | - | - | - | - | - | - | - |
|  | Patient-to-nurse ratio | 1 | - | - | - | - | 1 | - | - | - | - | - | - | - | - | - |
|  | Perspectives and experiences of older people, families, and multidisciplinary team members | - | - | 1 | - | - | - | - | - | - | - | - | - | - | - | - |
|  | Physician awareness and perceptions | - | - | - | - | - | - | - | - | - | - | - | - | 1 | - | - |
|  | Poor communication and lack of education about discharge | - | - | - | - | - | - | - | - | - | - | - | - | - | 1 | - |
|  | Postoperative complication | 1 | - | - | - | - | 1 | - | - | - | - | - | 1 | - | - | - |
|  | Postoperative vascular events | 1 | - | - | - | - | 1 | - | - | - | - | - | - | - | - | - |
|  | Preadmission concerns about the patient managing in the community (needing assistance with toileting and mobilizing) | - | - | - | - | - | 1 | - | - | - | - | - | - | - | - | - |
|  | Preoperative weight loss | 1 | - | - | - | - | 1 | - | - | - | - | - | - | - | - | - |
|  | Previous hospitalization | 1 | - | 1 | - | - | - | - | - | - | - | - | - | - | - | - |
|  | Pandemic-related pressures | - | - | 1 | - | - | - | - | - | - | - | - | - | - | 1 | - |
|  | Pressure to accept nursing home placement and lack of reasonable choices for home and community-based care. | - | - | - | - | - | - | - | - | - | - | - | - | - | 1 | - |
|  | Prior primary care provider (PCP) | - | - | - | - | - | - | - | - | - | - | 1 | - | - | - | - |
|  | Proximity to home, prior experience with the facility | - | - | 1 | - | - | - | - | - | - | - | - | - | - | - | - |
|  | Quality of planning and support for hospital-to-home transitions | - | - | - | - | - | - | - | - | - | - | - | - | 1 | - | - |
|  | Recent prior LTC stay | 1 | - | - | - | - | - | - | - | - | - | - | 1 | - | - | - |
|  | Refusal of care | - | - | 2 | - | - | - | - | - | - | - | - | - | - | - | - |
|  | Rehabilitation tolerance | - | - | - | - | - | - | - | - | - | - | - | - | 1 | - | - |
|  | Relationships with inpatient and outpatient healthcare providers | - | - | - | - | - | - | - | - | - | - | - | - | 1 | - | - |
|  | Religion and ‘god’ promoting adaptation | - | - | 1 | - | - | - | - | - | - | - | - | - | - | - | - |
|  | Respiratory treatment | 1 | - | - | - | - | 1 | - | - | - | - | - | 1 | - | - | - |
|  | Shared vision across services | - | - | - | - | - | - | - | - | - | - | - | - | 1 | - | - |
|  | Sputum suctioning | - | - | - | - | - | - | - | - | - | - | - | 1 | - | - | - |
|  | Bed availability | 1 | - | 4 | - | - | - | - | - | - | - | - | - | - | - | - |
|  | Staff unwillingness to enter certain neighborhoods due to crime and safety concern | - | - | - | - | - | - | - | - | - | - | - | - | - | 1 | - |
|  | Surgery type and Acute Physiology and Chronic Health Evaluation III Score | 1 | - | - | - | - | 1 | - | - | - | - | - | - | - | - | - |
|  | Resource availability | - | - | 3 | - | - | 2 | - | - | - | - | 1 | - | - | 3 | - |
|  | The concept of a last chance at home before moving to a care home | - | - | 1 | - | - | - | - | - | - | - | - | - | - | - | - |
|  | The decision-making process involving older adults, family members, nursing staff | - | - | 1 | - | - | - | - | - | - | - | - | - | - | - | - |
|  | The degree of familial support | - | - | 4 | - | - | - | - | - | - | - | - | - | 2 | - | - |
|  | The level of preparation for relocation | - | - | 1 | - | - | - | - | - | - | - | - | - | - | - | - |
|  | The number of additional procedures conducted during hospitalization | 2 | - | - | - | - | - | - | - | - | - | - | - | - | - | - |
|  | Transportation | - | - | - | - | 1 | - | - | - | - | - | - | - | - | - | - |
|  | Tube insertion in hospital | 1 | - | - | - | - | - | - | - | - | - | - | - | - | - | - |
|  | Unawareness of support services in the community | - | - | - | - | - | - | - | - | - | - | - | - | - | 1 | - |
|  | Use of general anesthesia | 1 | - | - | - | - | 1 | - | - | - | - | - | - | - | - | - |
|  | Vasopressor administration | - | - | - | - | - | - | - | - | - | - | - | 1 | - | - | - |

## **S5. Detailed factors influencing community reintegration transitions**

### **S5.1. Factors influencing transitions from LTC to home care**

**Socio-demographic characteristics.** Age was a significant factor in quantitative studies, with younger individuals more likely to transition to home care (n=3) [79–81], while older age was identified as a barrier (n=4) [82–85]. Gender also influenced transitions, with female gender positively associated with home care transitions in several studies (n=3) [79–81]; however, one study identified male gender as a facilitating factor (n=1) [83].

Race and ethnicity demonstrated consistent patterns, with non-white and Hispanic populations showing a higher likelihood of transitioning to home care (n=2) [79,82]. Living arrangements were also influential, with quantitative studies indicating that support systems and larger households facilitated transitions (n=2) [79,86]. Conversely, both quantitative (n=1) [85] and qualitative (n=2) [87,88] studies identified living alone and social isolation as barriers. Marital status also played a role, as being married facilitated transitions (n=1) [81], while being unmarried hindered them (n=1) [84]. In terms of socioeconomic factors, higher median family income supported transitions (n=1) [89], whereas housing challenges posed barriers (n=1) [82].

**Caregiver support.** Multiple aspects of caregiver support were influential in transitions from LTC to home care. Facilitating factors were identified across various study types. Mixed methods research underscored the importance of primary caregiver availability (n=1) [86], review evidence highlighted the overall significance of caregiver support (n=1) [81], and qualitative research demonstrated the positive impact of strong care partner support and resources (n=1) [90]. However, several caregiving-related barriers were also documented. Quantitative studies identified issues with family members and unpaid caregivers as obstacles to successful transitions (n=1) [82]. Qualitative research pointed to insufficient family caregiving support and the potential for caregiver burnout as significant barriers (n=2) [88,90]. Mixed methods research further emphasized high caregiver burden as a major challenge (n=1) [91].

**Health conditions.** Cognitive impairment, particularly dementia and memory-related problems, stands out as a major barrier to transitioning from LTC to home care. This is frequently cited in quantitative studies (n=7) [79,80,82–85,92] and further supported by qualitative and review evidence (n=2) [81,88]. Mental health conditions also emerged as significant determinants in quantitative (n=4) [80,82,91,92] and qualitative studies (n=1) [88], with depression, anxiety, and mental health disabilities identified as barriers. Behavioral challenges were consistently documented as barriers in quantitative research (n=3) [80,84,92].

In terms of physical health, quantitative evidence indicated that relatively better health facilitated transitions (n=1) [80], and review evidence emphasized the importance of medical stability (n=1) [81]. Conversely, physical health challenges were identified as barriers in both quantitative (n=3) [82,86,92] and qualitative research (n=1) [88]. Functional status also emerged as a critical determinant. Quantitative studies highlighted ADL dependencies as significant barriers to transitions (n=3) [84,91,92], while greater functional independence was associated with successful home transitions (n=3) [80,81,86].

**Healthcare system.** Multiple facility characteristics and operational factors influence transitions from LTC to home care. Regarding physical facility characteristics, quantitative studies revealed contradictory findings on facility size: one study reported that a larger facility size facilitated transitions (n=1) [92], whereas another represented it as a barrier (n=1) [83]. Higher occupancy rates showed a positive influence on transitions (n=1) [92]. Ownership type also exhibited varying effects. For-profit status facilitated transitions for long-stay residents in one study (n=1) [92], whereas another study found that public ownership positively influenced transitions compared to private facilities (n=1) [83].

In terms of staffing, research consistently showed that higher RN-to-total nurse staffing ratios (n=1) [89] and more staff per 100 beds (n=1) [83] facilitated transitions. Several operational barriers were identified in quantitative research, including longer average length of stay (n=1) [92], and challenges with facility-related services and supports (n=1) [82]. Qualitative evidence further highlighted that ineffective discharge procedures (n=1) [88] and limited availability of care services (n=1) [90] hindered successful transitions.

**Reimbursement and Funding Policies.** Quantitative research identified several factors that positively influence transitions from LTC to home care in the U.S. context, including higher state Medicaid spending on home care (n=1) [89], more generous nursing home reimbursements (n=1) [89], higher Medicare Advantage penetration (n=1) [89], the absence of bed-hold policies (n=1) [89], and a greater proportion of Medicare-funded residents (n=1) [92]. Commentary evidence further emphasized the importance of government investment in home-based care models (n=1) [87], noting that countries investing 3-3.7% of GDP in home care achieve stronger support for transitions.

**Person-centered care.** Patient preferences considerably influence transitions, with quantitative evidence indicating that residents who expressed a desire for community discharge were 8.82 times more likely to transition successfully (n=1) [80]. Mixed methods research supported this finding, highlighting the importance of both residents’ desire to return to the community (n=1) [86] and person-centered care planning approaches (n=1) [91]. Review evidence reinforced these patterns (n=2) [81,87], underscoring how individual and family preferences for home care, alongside resident-directed care and respect for autonomy, positively influenced transitions.

### **S5.2. Factors influencing transitions from CCC to home care**

**Socio-demographic characteristics.** Age was consistently represented as an influential factor in quantitative (n=3) [93–95] and a review study (n=1) [96] , with older age acting as a barrier to home transitions. Gender also appeared as a determinant in quantitative research, with female sex associated with successful transitions (n=1) [97]. Race and ethnicity demonstrated notable patterns across study types. Quantitative research identified positive associations between non-white populations and successful transitions (n=1) [98], a finding further supported by a systematic review (n=1) [96] that highlighted white ethnicity as a barrier to transitions.

Living arrangements showed substantial influence in quantitative studies. Having a support person and not living alone were noted as facilitating factors (n=2) [97,99], while living alone consistently appeared as a barrier (n=3) [94,98,100]. Marital status demonstrated clear associations in systematic review evidence (n=1) [96], with being married facilitating transitions and single status hindering them.

**Caregiver support.** In transitions from CCC settings, caregiver support was less frequently documented but showed a clear influence. Quantitative evidence identified having a support person with a positive attitude toward discharge as a facilitating factor (n=1) [97]. Although fewer studies examined caregiver support in this setting compared to LTC transitions, the available evidence underscored the importance of early caregiver involvement in discharge planning.

**Health conditions.** Cognitive impairment was identified as a significant obstacle to transitions in quantitative studies (n=5) [94,97–99,101], a finding further supported by systematic review evidence (n=1) [96]. Mental health factors, particularly depression, negatively influenced transitions in quantitative research (n=1) [100], with systematic reviews similarly identifying depression as a barrier (n=1) [96].

Physical health complexities, including higher comorbidity, were also barriers in quantitative studies (n=3) [93,100,101]. Functional status showed its influence across multiple indicators. Quantitative and review studies identified reduced functional independence and decreased ADL performance as significant barriers to transitions (n=8) [93,94,96–98,100–102], whereas higher motor function scores were found to facilitate transitions (n=1) [103]. Mobility status was another important factor, with better mobility at discharge supporting transitions (n=1) [99] and mobility impairments hindering them (n=1) [94]. Moreover, older adults’ positive beliefs about their capability to improve independence with ADLs (n=1) [104] promoted successful transitions. Lastly, continence status was relevant, with incontinence identified as a barrier in quantitative evidence (n=1) [98].

**Healthcare system.** Staffing was a critical factor, with quantitative studies identifying lower bed-to-nurse ratios (n=1) [105] and higher registered nurse (RN) staffing levels (n=1) [95] as facilitators of transitions. Conversely, higher ratios of licensed practical nurses (LPNs) to RNs were highlighted as barriers (n=1) [105]. Facility types also influenced transitions, with hospital-based and nonprofit facilities demonstrating higher discharge rates (n=1) [95]. Several operational factors were also positively associated with successful transitions. Specifically, a greater density of specialty patients (n=1) [105], higher volumes of specific conditions (such as hip fracture admissions) (n=1) [95], and the presence of dedicated rehabilitation units (n=1) [95] all facilitated home care placement. Structured operational models also showed positive effects, with the Siebens Domain Management Model (SDMM) improving discharge rates and reducing length of stay (n=1) [106]. Therapy intensity was another significant factor, with higher-intensity therapy (>60 minutes/day or >5.3 hours/week) associated with increased home care transition rates and shorter lengths of stay (n=2) [101,107].

**Reimbursement and funding policies.** Quantitative research identified Medicaid enrollment as a barrier to successful transitions from CCC to home care (n=1) [98], while broader Medicaid home care coverage was found to facilitate these transitions (n=1) [108].

**Person-centered care.** Quantitative research identified several person-centered facilitating factors, including individuals’ desire to return to the community (n=1) [97], positive beliefs from both older adults and staff regarding capability (n=1) [104], and the use of structured person-centered care models (n=1) [106]. Conversely, negative beliefs from either patients or staff about the ability for independence were reported as barriers to successful transitions (n=1) [104].

## **S6. Frequency of influential factors in community reintegration transitions**

Table S4 presents the complete list of factors influencing the transition of older adults from LTC and CCC to home care and their frequency.

**Table S4.** Full list of community reintegration factors

| Factors associated with older adults’ community reintegration transitions | | LTC to home care | | | | | CCC to home care | | | | |
| --- | --- | --- | --- | --- | --- | --- | --- | --- | --- | --- | --- |
|  |  | Quantitative | | Qualitative | | | Quantitative | | Qualitative | | |
|  |  | Positive | Negative | Positive | Negative | Not specified | Positive | Negative | Positive | Negative | Not specified |
| Socio-demographic characteristics | | | | | | | | | | | |
|  | Gender (Female) | 3 | - | - | - | - | 1 | - | - | - | - |
|  | Gender (male) | 1 | - | - | - | - | - | - | - | - | - |
|  | Higher median family income | 1 | - | - | - | - | - | - | - | - | - |
|  | Housing challenges | - | 1 | - | - | - | - | - | - | - | - |
|  | Living alone and isolation | - | 1 | - | 2 | - | - | 3 | - | - | - |
|  | Married | 1 | - | - | - | - | 1 | - | - | - | - |
|  | Race (non-white and Hispanic ethnicity) | 2 | - | - | - | - | 1 | - | 1 | - | - |
|  | Older age | - | 4 | - | - | - | - | 3 | - | - | - |
|  | Support systems/larger household | 2 | - | - | - | - | 2 | - | - | - | - |
|  | Unmarried | - | 1 | - | - | - | - | - | - | - | - |
|  | Younger age | 3 | - | - | - | - | 1 | - | - | - | - |
| Caregiver support | | | | | | | | | | | |
|  | Caregiver availability | - | - | 1 | - | - | - | - | - | - | - |
|  | Caregiver burden | - | - | - | 1 | - | - | - | - | - | - |
|  | Family/unpaid caregiver issues | - | 1 | - | - | - | - | - | - | - | - |
|  | Insufficient support | - | - | - | 2 | - | - | - | - | - | - |
|  | Strong care partner support | - | - | 1 | - | - | - | - | - | - | - |
|  | Supporting person positive attitude | - | - | - | - | - | 1 | - | - | - | - |
| Health conditions | | | | | | | | | | | |
|  | ADL dependencies | - | 3 | - | - | - | - | 4 | - | - | - |
|  | Better health/medical stability | 1 | - | 1 | - | - | - | - | - | - | - |
|  | Behavioral challenges | - | 3 | - | - | - | - | - | - | - | - |
|  | Cognitive impairment (including Dementia) | - | 7 | - | 1 | - | - | 3 | - | 1 | - |
|  | Continence | 2 | - | - | - | - | - | 1 | - | - | - |
|  | Depression/anxiety | - | 4 | - | 1 | - | - | 1 | - | 1 | - |
|  | Functional Independence | 3 | - | - | - | - | 1 | - | - | - | - |
|  | Greater comorbidities (the Charlson Comorbidity Index) | - | - | - | - | - | - | 2 | - | - | - |
|  | Having had a hip fracture | 1 | - | - | - | - | - | - | - | - | - |
|  | Mobility status | - | - | - | - | - | 1 | 1 | - | - | - |
|  | No Active cardiac pathology | - | - | - | - | - | 1 | - | - | - | - |
|  | Physical health challenges | - | 3 | - | 1 | - | - | 2 | - | - | - |
|  | Relatively better health (not being in the end stages of a disease and no heart failure diagnosis) | 3 | - | 1 | - | - | - | - | - | - | - |
|  | Substance use | - | - | - | 1 | - | - | - | - | - | - |
|  | The resident’s Return to Community Initiative’s score | 1 | - | - | - | - | - | - | - | - | - |
| Healthcare system (operational efficiency) | | | | | | | | | | | |
|  | Length of stay | - | 1 | - | - | - | - | - | - | - | - |
|  | Staff per 100 beds | 1 | - | - | - | - | - | - | - | - | - |
|  | Concerns about access to healthcare and personal safety in the community | - | - | - | 1 | - | - | - | - | - | - |
|  | Consistency of LTCF care | 2 | - | - | - | - | - | - | - | - | - |
|  | Consumer engagement challenges | - | 1 | - | - | - | - | - | - | - | - |
|  | Expanding the number of home care workers | - | - | 1 | - | - | - | - | - | - | - |
|  | Facility size | 1 | 1 | - | - | - | - | - | - | - | - |
|  | Ownership type | 1 | 1 | - | - | - | 1 | - | - | - | - |
|  | Implementation of the Siebens Domain Management Model (SDMM) | - | - | - | - | - | 1 | - | - | - | - |
|  | Improved information about community-based care options | - | - | 1 | - | - | - | - | - | - | - |
|  | LPN to RN ratio | - | - | - | - | - | - | 1 | - | - | - |
|  | Occupancy rate | 1 | - | - | - | - | - | - | - | - | - |
|  | Possible medical complications that may occur during geriatric IR | - | - | - | - | - | - | 1 | - | - | - |
|  | Proportion of Medicaid residents | - | 1 | - | - | - | - | - | - | - | - |
|  | Receiving Medicare-certified home health agency services | 1 | - | - | - | - | - | - | - | - | - |
|  | Rehabilitation programs | 1 | - | - | - | - | 1 | - | - | - | - |
|  | Registered Nurse-to-total nurse ratio | 1 | - | - | - | - | 1 | - | - | - | - |
|  | Rural location | - | 1 | - | - | - | - | - | - | - | - |
|  | Service availability | - | 1 | - | 2 | - | - | - | - | - | - |
|  | Specialty patient density | - | - | - | - | - | 1 | - | - | - | - |
|  | Specialized support targeting mental health and substance use needs | - | - | 1 | - | - | - | - | - | - | - |
|  | The proportion of residents admitted directly from acute care facilities | 2 | - | 1 | - | - | - | - | - | - | - |
|  | Therapy Intensity (physical therapy, occupational therapy, and speech therapy) | 1 | - | - | - | - | 1 | - | - | - | - |
|  | Undergoing rehab services | 1 | - | - | - | - | - | - | - | - | - |
|  | Volume of hip fracture admissions | 1 | - | - | - | - | - | - | - | - | - |
| Reimbursement and funding policies | | | | | | | | | | | |
|  | Bed-hold policies | 1 | - | - | - | - | - | - | - | - | - |
|  | Medicaid spending on home care | 1 | - | - | - | - | - | - | - | - | - |
|  | Medicare-funded residents | 1 | - | - | - | - | - | - | - | - | - |
|  | Medicare Advantage | 1 | - | - | - | - | - | - | - | - | - |
|  | LTC reimbursements | 1 | - | - | - | - | - | - | - | - | - |
|  | Medicaid enrolment | - | 1 | - | - | - | - | 1 | - | - | - |
| Person-centered care | | | | | | | | | | | |
|  | Having a preference/desire for discharge to the community | 1 | - | 3 | - | - | 1 | - | - | - | - |
|  | Negative beliefs about patient’s capability to increase independence with ADLs | - | - | - | - | - | - | 1 | - | - | - |
|  | PCC planning approaches | - | - | 2 | - | - | 1 | - | - | - | - |
|  | Positive beliefs about the patient’s capability to increase independence with ADLs | - | - | - | - | - | - | 1 | - | - | - |
|  | Resident autonomy | - | - | 1 | - | - | - | - | - | - | - |

# **References**

[1] Gaughan J, Gravelle H, Santos R, Siciliani L. Long-term care provision, hospital bed blocking, and discharge destination for hip fracture and stroke patients. Int J Health Econ Manag 2017;17:311–31. https://doi.org/10.1007/s10754-017-9214-z.

[2] Young DL, Engels R, Colantuoni E, Friedman LA, Hoyer EH. Machine learning prediction of hospital patient need for post-acute care using an admission mobility measure is robust across patient diagnoses. Health Policy and Technology 2023;12:100754. https://doi.org/10.1016/j.hlpt.2023.100754.

[3] Kasteridis P, Mason A, Goddard M, Jacobs R, Santos R, Rodriguez-Sanchez B, et al. Risk of Care Home Placement following Acute Hospital Admission: Effects of a Pay-for-Performance Scheme for Dementia. PLoS One 2016;11:e0155850. https://doi.org/10.1371/journal.pone.0155850.

[4] Moriwaki M, Hayashida K, Ogata Y. Factors associated with non-home discharge of patients hospitalized for hip fracture: A nationwide retrospective study using the Japanese diagnostic procedure combination database. Medicine (Baltimore) 2023;102:e33138. https://doi.org/10.1097/MD.0000000000033138.

[5] Inneh IA, Clair AJ, Slover JD, Iorio R. Disparities in Discharge Destination After Lower Extremity Joint Arthroplasty: Analysis of 7924 Patients in an Urban Setting. Journal of Arthroplasty 2016;31:2700–4. https://doi.org/10.1016/j.arth.2016.05.027.

[6] van Dartel D, Vermeer M, Folbert EC, Arends AJ, Vollenbroek-Hutten MMR, Hegeman JH. Early Predictors for Discharge to Geriatric Rehabilitation after Hip Fracture Treatment of Older Patients. J AM MED DIR ASSOC 2021;22:2454–60. https://doi.org/10.1016/j.jamda.2021.03.026.

[7] Kramer DB, Reynolds MR, Normand S-L, Parzynski CS, Spertus JA, Mor V, et al. Nursing Home Use After Implantable Cardioverter-Defibrillator Implantation in Older Adults: Results from the National Cardiovascular Data Registry. J Am Geriatr Soc 2017;65:340–7. https://doi.org/10.1111/jgs.14520.

[8] Schwarzkopf R., Ho J., Quinn J.R., Snir N., Mukamel D. Factors Influencing Discharge Destination After Total Knee Arthroplasty: A Database Analysis. Geriatr Orthop Surg Rehabit 2016;7:95–9. https://doi.org/10.1177/2151458516645635.

[9] Ross JH, Wood N, Simmons A, Lua-Mailland LL, Wallace SL, Chapman GC. Nonhome Discharge in Patients Undergoing Pelvic Reconstructive Surgery: A National Analysis. Urogynecology (Phila) 2023. https://doi.org/10.1097/SPV.0000000000001347.

[10] Okoh A.K., Ozturk E., Gold J., Siddiqui E., Dhaduk N., Haik B., et al. Risk scoring model for prediction of non-home discharge after transcatheter aortic valve replacement. J Geriatr Cardiol 2020;17:621–7. https://doi.org/10.11909/j.issn.1671-5411.2020.10.002.

[11] Dutta D, Thornton D, Bowen E. Using population-based routinely collected data from the Sentinel Stroke National Audit Programme to investigate factors associated with discharge to care home after rehabilitation. Clinical Rehabilitation 2018;32:1108–18. https://doi.org/10.1177/0269215517748715.

[12] Schaefer MS, Hammer M, Platzbecker K, Santer P, Grabitz SD, Murugappan KR, et al. What Factors Predict Adverse Discharge Disposition in Patients Older Than 60 Years Undergoing Lower-extremity Surgery? The Adverse Discharge in Older Patients after Lower-extremity Surgery (ADELES) Risk Score. Clin Orthop 2021;479:546–7. https://doi.org/10.1097/CORR.0000000000001532.

[13] Ramanan M, Kumar A, Anstey C, Shekar K. Non-home discharge after cardiac surgery in Australia and New Zealand: a cross-sectional study. BMJ Open 2021;11:e049187. https://doi.org/10.1136/bmjopen-2021-049187.

[14] Schumacher R, Walder B, Delhumeau C, Müri RM. Predictors of inpatient (neuro)rehabilitation after acute care of severe traumatic brain injury: An epidemiological study. Brain Inj 2016;30:1186–93. https://doi.org/10.1080/02699052.2016.1183821.

[15] Burton JK, Ferguson EEC, Barugh AJ, Walesby KE, MacLullich AMJ, Shenkin SD, et al. Predicting Discharge to Institutional Long-Term Care After Stroke: A Systematic Review and Metaanalysis. J Am Geriatr Soc 2018;66:161–9. https://doi.org/10.1111/jgs.15101.

[16] Thomeer MB, Mudrazija S, Angel JL. How do race and Hispanic ethnicity affect nursing home admission? Evidence from the Health and Retirement Study. J Gerontol B Psychol Sci Soc Sci 2015;70:628–38. https://doi.org/10.1093/geronb/gbu114.

[17] Wahlsten LR, Smedegaard L, Brorson S, Gislason G, Palm H. Living settings and cognitive impairment are stronger predictors of nursing home admission after hip fracture surgery than physical comorbidities A nationwide Danish cohort study. Injury 2020;51:2289–94. https://doi.org/10.1016/j.injury.2020.06.041.

[18] Basic D, Shanley C. Frailty in an older inpatient population: using the clinical frailty scale to predict patient outcomes. J Aging Health 2015;27:670–85. https://doi.org/10.1177/0898264314558202.

[19] Misquitta K, Reid N, Hubbard RE, Gordon EH. Factors associated with entry to residential care in frail older inpatients. Australas J Ageing 2023;42:720–7. https://doi.org/10.1111/ajag.13231.

[20] Burton JK, Ciminata G, Lynch E, Shenkin SD, Geue C, Quinn TJ. Understanding Pathways into Care homes using Data (UnPiCD study): a retrospective cohort study using national linked health and social care data. Age and Ageing 2022;51. https://doi.org/10.1093/ageing/afac304.

[21] Lera J, Pascual-Sáez M, Cantarero-Prieto D. Socioeconomic Inequality in the Use of Long-Term Care among European Older Adults: An Empirical Approach Using the SHARE Survey. Int J Environ Res Public Health 2020;18:20. https://doi.org/10.3390/ijerph18010020.

[22] Kosteniuk JG, Morgan DG, Elliot V, Froehlich Chow A, Bayly M, Watson E, et al. A Scoping Review of Care Trajectories across Multiple Settings for Persons with Dementia. Can J Aging 2022;41:71–95. https://doi.org/10.1017/S0714980821000167.

[23] Sizing Up the Challenge: Meeting the Demand for Long-Term Care in Canada. The Conference Board of Canada 2017. https://www.conferenceboard.ca/product/sizing-up-the-challenge-meeting-the-demand-for-long-term-care-in-canada/ (accessed July 30, 2023).

[24] Mitchell R, Harvey L, Draper B, Brodaty H, Close J. Risk factors associated with residential aged care, respite and transitional aged care admission for older people following an injury-related hospitalisation. Archives of Gerontology and Geriatrics 2017;72:59–66. https://doi.org/10.1016/j.archger.2017.05.012.

[25] Conca A, Koch D, Regez K, Kutz A, Haubitz S, Schuetz P, et al. Sciendo. International Journal of Health Professions 2022;9:59–66. https://doi.org/10.2478/ijhp-2022-0005.

[26] Harrison JK, Walesby KE, Hamilton L, Armstrong C, Starr JM, Reynish EL, et al. Predicting discharge to institutional long-term care following acute hospitalisation: a systematic review and meta-analysis. Age Ageing 2017;46:547–58. https://doi.org/10.1093/ageing/afx047.

[27] Tollette J, Heh V, Wiseman JM, Quatman-Yates CC, Moroi S, Quatman CE. Impact of vision impairment on discharge destination for patients with hip fracture. J Clin Orthop Trauma 2024;50:102377. https://doi.org/10.1016/j.jcot.2024.102377.

[28] Canadian Institute for Health Information,. Seniors in Transition - Exploring Pathways Across the Care Continuum. 2017.

[29] Brown CL, Menec V. Health, Social, and Functional Characteristics of Older Adults With Continuing Care Needs: Implications for Integrated Care. Journal of Aging & Health 2019;31:1085–105. https://doi.org/10.1177/0898264318759856.

[30] Sebban A, Lesclide E, Bonin-Guillaume S, Campana M, Grino M, Franqui C. Previous in-home physiotherapy prevents institutionalization after short-term hospitalization in community-dwelling older dependent people. Aging Clinical and Experimental Research 2020;32:1271–7. https://doi.org/10.1007/s40520-019-01310-7.

[31] Tochimoto S, Kitamura M, Hino S, Kitamura T. Predictors of home discharge among patients hospitalized for behavioural and psychological symptoms of dementia. Psychogeriatrics 2015;15:248–54. https://doi.org/10.1111/psyg.12114.

[32] Koskas P, Pons-Peyneau C, Romdhani M, Houenou-Quenum N, Galleron S, Drunat O. Hospital Discharge Decisions Concerning Older Patients: Understanding the Underlying Process. Can J Aging 2019;38:90–9. https://doi.org/10.1017/S0714980818000442.

[33] Glance LG, Joynt Maddox KE, Stone PW, Shang J, Furuya EY, Chastain AM, et al. COVID-19 Pandemic and Racial and Ethnic Disparities in Long-Term Nursing Home Stay or Death Following Hospital Discharge. JAMA Netw Open 2025;8:e2456816. https://doi.org/10.1001/jamanetworkopen.2024.56816.

[34] Kobewka DM, Mulpuru S, Chassé M, Thavorn K, Lavallée LT, English SW, et al. Predicting the need for supportive services after discharged from hospital: a systematic review. BMC Health Services Research 2020;20:161. https://doi.org/10.1186/s12913-020-4972-6.

[35] Kuzmik A, BeLue R, Resnick B, Rodriguez M, Berish D, Galvin JE, et al. Caregiver preparedness is associated with desire to seek long-term care admission of hospitalized persons with dementia. Int J Geriatr Psychiatry 2023;38:e6006. https://doi.org/10.1002/gps.6006.

[36] Callahan CM, Tu W, Unroe KT, LaMantia MA, Stump TE, Clark DO. Transitions in Care in a Nationally Representative Sample of Older Americans with Dementia. J Am Geriatr Soc 2015;63:1495–502. https://doi.org/10.1111/jgs.13540.

[37] Mitsutake S, Ishizaki T, Edahiro A, Kitamura A, Hirata T, Saito A. The effects of dental visits on the occurrence of acute hospitalization for systemic diseases among patients aged 75 years or older: A propensity score-matched study. Archives of Gerontology and Geriatrics 2023;107. https://doi.org/10.1016/j.archger.2022.104876.

[38] University M. Evidence-based considerations around long-term care. Default n.d. https://www.mcmasteroptimalaging.org/blog/detail/blog/2022/08/31/evidence-based-considerations-around-long-term-care (accessed July 30, 2023).

[39] Don Drummond. Ageing Well - School of Policy Studies - Queen’s University. 2020.

[40] Ellis HL, Wan B, Yeung M, Rather A, Mannan I, Bond C, et al. Complementing chronic frailty assessment at hospital admission with an electronic frailty index (FI-Laboratory) comprising routine blood test results. CMAJ 2020;192:E3–8. https://doi.org/10.1503/cmaj.190952.

[41] Darvall JN, Loth J, Bose T, Braat S, De Silva A, Story DA, et al. Accuracy of the Clinical Frailty Scale for perioperative frailty screening: a prospective observational study. Can J Anaesth 2020;67:694–705. https://doi.org/10.1007/s12630-020-01610-x.

[42] Sastry RA, Feler JR, Shao B, Ali R, McNicoll L, Telfeian AE, et al. Frailty independently predicts unfavorable discharge in non-operative traumatic brain injury: A retrospective single-institution cohort study. PLoS ONE 2022;17. https://doi.org/10.1371/journal.pone.0275677.

[43] Carbone S, Kokorelias KM, Berta W, Law S, Kuluski K. Stakeholder involvement in care transition planning for older adults and the factors guiding their decision-making: a scoping review. BMJ Open 2022;12:e059446. https://doi.org/10.1136/bmjopen-2021-059446.

[44] Ahsberg E. Discharge from hospital - a national survey of transition to out-patient care. Scand J Caring Sci 2019;33:329–35. https://doi.org/10.1111/scs.12625.

[45] Henning-Smith C, Kozhimannil KB, Casey MM, Prasad S. Beyond Clinical Complexity: Nonmedical Barriers to Nursing Home Care for Rural Residents. Journal of Aging and Social Policy 2018;30:109–26. https://doi.org/10.1080/08959420.2018.1430413.

[46] Wieczorek E, Kocot E, Evers S, Sowada C, Pavlova M. Key Care Provision Aspects That Affect Care Transition in the Long-Term Care Systems: Preliminary Review Findings. International Journal of Environmental Research and Public Health 2022;19:6402. https://doi.org/10.3390/ijerph19116402.

[47] Lage DE, Jernigan MC, Chang Y, Grabowski DC, Hsu J, Metlay JP, et al. Living Alone and Discharge to Skilled Nursing Facility Care after Hospitalization in Older Adults. J Am Geriatr Soc 2018;66:100–5. https://doi.org/10.1111/jgs.15150.

[48] Pattath P, Odom EC, Tong X, Yin X, Coleman King SM. A Comparison of Acute Ischemic Stroke Patients Discharged to Inpatient Rehabilitation vs a Skilled Nursing Facility: The Paul Coverdell National Acute Stroke Program. Arch Phys Med Rehabil 2023;104:605–11. https://doi.org/10.1016/j.apmr.2022.11.008.

[49] Yang M-T, Temkin-Greener H, Veazie P, Cai S. Post-acute care transitions during COVID-19: Racial, ethnic, and socioeconomic differences in older adults with Alzheimer’s disease and related dementia. J Am Geriatr Soc 2024;72:2006–16. https://doi.org/10.1111/jgs.18884.

[50] Alberta health services continuing care. Moving to a new home in continuing care An information and decision-making guide for patients and families 2019.

[51] Koch D, Schuetz P, Haubitz S, Kutz A, Mueller B, Weber H, et al. Improving the post-acute care discharge score (PACD) by adding patients’ self-care abilities: A prospective cohort study. PLOS ONE 2019;14:e0214194. https://doi.org/10.1371/journal.pone.0214194.

[52] Yeates EO, Grigorian A, Kuza CM, Nguyen NT, Inaba K, Dolich M, et al. The DEPARTS Score: A Novel Tool for Predicting Discharge Disposition in Geriatric Trauma Patients. Am Surg 2023;89:447–51. https://doi.org/10.1177/00031348211029843.

[53] de Groot AJ, Wattel EM, van Balen R, Hertogh CMPM, van der Wouden JC. Referral to Geriatric Rehabilitation in the Netherlands, an Exploratory Study of Patient Characteristics. Rehabil Nurs 2025;50:66–77. https://doi.org/10.1097/RNJ.0000000000000489.

[54] Temkin-Greener H, Yan D, Cai S. Post-acute care transitions and outcomes among Medicare beneficiaries with dementia: Associations with race/ethnicity and dual status. Health Serv Res 2023;58:164–73. https://doi.org/10.1111/1475-6773.14059.

[55] Morris RS, Tignanelli CJ, deRoon-Cassini T, Laud P, Sparapani R. Improved Prediction of Older Adult Discharge After Trauma Using a Novel Machine Learning Paradigm. J Surg Res 2022;270:39–48. https://doi.org/10.1016/j.jss.2021.08.021.

[56] Hirota Y, Shin J-H, Sasaki N, Kunisawa S, Fushimi K, Imanaka Y. Development and validation of prediction models for the discharge destination of elderly patients with aspiration pneumonia. PLoS One 2023;18:e0282272. https://doi.org/10.1371/journal.pone.0282272.

[57] Pappadis MR, Malagaris I, Kuo Y, Leland N, Freburger J, Goodwin JS. Care patterns and predictors of community residence among older patients after hospital discharge for traumatic brain injury. Journal of the American Geriatrics Society 2023;71:1806–18. https://doi.org/10.1111/jgs.18308.

[58] Kubo K, Kamo T, Momosaki R, Mitsutomi K. Development of a Point System to Predict Discharge to Home for Acute Stroke Patients. PM and R 2021;13:38–44. https://doi.org/10.1002/pmrj.12371.

[59] Sasaki S, Hayashi T, Masuda M, Kawano O, Yamamoto T, Maeda T. Factors Affecting Home Discharge of Older Adults with Cervical Spinal Cord Injury in Japan Regional Population. Spine Surg Relat Res 2023;7:482–7. https://doi.org/10.22603/ssrr.2023-0045.

[60] Gentili S, Calderón-Larrañaga A, Rizzuto D, Gordon AL, Agerholm J, Lennartsson C, et al. Predictors of 15-year transitions across living and care settings in a population of Swedish older adults. Age Ageing 2025;54:afaf006. https://doi.org/10.1093/ageing/afaf006.

[61] Egbujie BA, Tran J, Hirdes JP. Multistate Competing Risk Analysis of Transition Back to the Community Among Long-Term Care Home (LTC) Destined Patients: A Brief Report. J Prim Care Community Health 2023;14:21501319231220742. https://doi.org/10.1177/21501319231220742.

[62] Takekawa T, Katagi M, Kobayashi K, Toyoda S, Nakamura T, Yoshida H, et al. Factors influencing home discharge of hospitalized oldest-old patients (≥90 years): A retrospective quantitative case-control study. Geriatric Nursing 2024;60:99–106. https://doi.org/10.1016/j.gerinurse.2024.08.021.

[63] Abbitt D, Choy K, Robinson TN, Jones EL, Horney C, Sommerville S, et al. Preoperative Risk Factors for Discharge to Facility After Surgery in Geriatric Patients. Am Surg 2024;90:2222–7. https://doi.org/10.1177/00031348241256056.

[64] Liljas AEM, Jensen NK, Pulkki J, Andersen I, Keskimäki I, Burström B, et al. The influence of sociodemographic factors and close relatives at hospital discharge and post hospital care of older people with complex care needs: nurses’ perceptions on health inequity in three Nordic cities. European Journal of Ageing 2022;19:189–200. https://doi.org/10.1007/s10433-022-00701-6.

[65] Miller EA, Intrator O, Gadbois E, Gidmark S, Rudolph JL. VA Staff Perceptions of Barriers and Facilitators to Home-and Community-Based Placement Post-Hospital Discharge. J Aging Soc Policy 2019;31:1–29. https://doi.org/10.1080/08959420.2018.1444889.

[66] Beck SH, Eilertsen G, Andersen-Ranberg K, Janssens A, Nielsen DS. In the footstep of the old patient from hospital to home: A qualitative field observation study. Scand J Caring Sci 2024;38:745–55. https://doi.org/10.1111/scs.13257.

[67] Welsh A, Hanson S, Pfeiffer K, Khoury R, Clark A, Grant K, et al. Facilitating the transition from hospital to home after hip fracture surgery: a qualitative study from the HIP HELPER trial. BMC Geriatrics 2024;24:948. https://doi.org/10.1186/s12877-024-05390-7.

[68] Barber B, Weeks L, Steeves-Dorey L, McVeigh W, Stevens S, Moody E, et al. Hospital to Home: Supporting the Transition From Hospital to Home for Older Adults. Canadian Journal of Nursing Research 2022;54:483–96. https://doi.org/10.1177/08445621211044333.

[69] Kirschner KL. Drama and Trauma: Unpacking Moral Distress in the Context of Discharge Planning. Narrat Inq Bioeth 2020;10:223–30. https://doi.org/10.1353/nib.2020.0067.

[70] Sun M, Liu L, Wang J, Zhuansun M, Xu T, Qian Y, et al. Facilitators and inhibitors in hospital-to-home transitional care for elderly patients with chronic diseases: A meta-synthesis of qualitative studies. Front Public Health 2023;11:1047723. https://doi.org/10.3389/fpubh.2023.1047723.

[71] Camicia M, Lutz BJ. Nursing’s Role in Successful Transitions Across Settings. Stroke 2016;47:e246–9. https://doi.org/10.1161/STROKEAHA.116.012095.

[72] D’Souza AN, Granger CL, Patrick CJ, Kay JE, Said CM. Factors Associated With Discharge Destination in Community-Dwelling Adults Admitted to Acute General Medical Units. J Geriatr Phys Ther 2021;44:94–100. https://doi.org/10.1519/JPT.0000000000000272.

[73] Shimoyama S, Ono T, Ebihara S. Geriatric nutritional risk index and 100-m walk achievement predict discharge to home in elderly patients with heart failure. Geriatr Gerontol Int 2020;20:1029–35. https://doi.org/10.1111/ggi.14014.

[74] Amakasu K, Inoue T, Watanabe Y. Low phase angle: A predictor of functional status and discharge disposition in acute stroke older patients. Clin Nutr ESPEN 2024;61:197–202. https://doi.org/10.1016/j.clnesp.2024.03.028.

[75] Donegan D, Teeling SP, McNamara M, McAweeney E, McGrory L, Mooney R. Calling time on the “dance of the blind reflex”: how collaborative working reduced older persons’ length of stay in acute care and increased home discharge. International Practice Development Journal 2021;11:1–14. https://doi.org/10.19043/ipdj.111.004.

[76] Agerholm J, Jensen NK, Liljas A. Healthcare professionals’ perception of barriers and facilitators for care coordination of older adults with complex care needs being discharged from hospital: A qualitative comparative study of two Nordic capitals. BMC Geriatr 2023;23:32. https://doi.org/10.1186/s12877-023-03754-z.

[77] Turbow SD, Ali MK, Culler SD, Rask KJ, Perkins MM, Clevenger CK, et al. Association of Fragmented Readmissions and Electronic Information Sharing With Discharge Destination Among Older Adults. JAMA Network Open 2023;6:e2313592. https://doi.org/10.1001/jamanetworkopen.2023.13592.

[78] Bestsennyy O, Chmielewski M, Koffel A. From facility to home: How healthcare could shift by 2025 | McKinsey 2022. https://www.mckinsey.com/industries/healthcare/our-insights/from-facility-to-home-how-healthcare-could-shift-by-2025 (accessed September 14, 2024).

[79] Mudrazija S, Thomeer MB, Angel JL. Gender Differences in Institutional Long-Term Care Transitions. Womens Health Issues 2015;25:441–9. https://doi.org/10.1016/j.whi.2015.04.010.

[80] Hass Z, Woodhouse M, Kane R, Arling G. Modeling Community Discharge of Medicaid Nursing Home Residents: Implications for Money Follows the Person. Health Services Research 2018;53:2787–802. https://doi.org/10.1111/1475-6773.12795.

[81] Freeman S, Bishop K, Spirgiene L, Koopmans E, Bothelo FC, Fyfe T, et al. Factors affecting residents transition from long term care facilities to the community: A scoping review. BMC Health Services Research 2017;17. https://doi.org/10.1186/s12913-017-2571-y.

[82] Robison J, Shugrue N, Porter M, Baker K. Challenges to community transitions through Money Follows the Person. Health Serv Res 2020;55:357–66. https://doi.org/10.1111/1475-6773.13267.

[83] Morita K, Ono S, Ishimaru M, Matsui H, Naruse T, Yasunaga H. Factors Affecting Discharge to Home of Geriatric Intermediate Care Facility Residents in Japan. Journal of the American Geriatrics Society 2018;66:728–34. https://doi.org/10.1111/jgs.15295.

[84] Abrahamson K, Hass Z, Arling G. Shall I Stay or Shall I Go? The Choice to Remain in the Nursing Home Among Residents With High Potential for Discharge. Journal of Applied Gerontology 2020;39:863–70. https://doi.org/10.1177/0733464818807818.

[85] Lam K, Cenzer I, Covinsky KE. Return to community living and mortality after moving to a long-term care facility: A nationally representative cohort study. Journal of the American Geriatrics Society 2023;71:569–76. https://doi.org/10.1111/jgs.18144.

[86] Buttke D, Cooke V, Abrahamson K, Shippee T, Davila H, Kane R, et al. A Statewide Model for Assisting Nursing Home Residents to Transition Successfully to the Community. Geriatrics (Basel) 2018;3:18. https://doi.org/10.3390/geriatrics3020018.

[87] Grabowski DC, Chen A, Saliba D. Paying for Nursing Home Quality: An Elusive But Important Goal. Journal of the American Geriatrics Society 2023;71:342–8. https://doi.org/10.1111/jgs.18260.

[88] Campbell-Enns HJ, Campbell M, Rieger KL, Thompson GN, Doupe MB. No Other Safe Care Option: Nursing Home Admission as a Last Resort Strategy. Gerontologist 2020;60:1504–14. https://doi.org/10.1093/geront/gnaa077.

[89] Xu H, Intrator O. Medicaid Long-term Care Policies and Rates of Nursing Home Successful Discharge to Community. J Am Med Dir Assoc 2020;21:248-253.e1. https://doi.org/10.1016/j.jamda.2019.01.153.

[90] Carbone S, Berta W, Law S, Kuluski K. Long-term care transitions during a global pandemic: Planning and decision-making of residents, care partners, and health professionals in Ontario, Canada. PLoS One 2023;18:e0295865. https://doi.org/10.1371/journal.pone.0295865.

[91] Kristof L, Fortinsky RH, Kellett K, Porter M, Robison J. Experiences of Informal Caregivers of Older Adults Transitioned From Nursing Homes to the Community Through the Money Follows the Person Demonstration. J Aging Soc Policy 2017;29:20–34. https://doi.org/10.1080/08959420.2016.1187034.

[92] Holup A.A., Gassoumis Z.D., Wilber K.H., Hyer K. Community Discharge of Nursing Home Residents: The Role of Facility Characteristics. Health Serv Res 2016;51:645–66. https://doi.org/10.1111/1475-6773.12340.

[93] Chan D.K.Y., Zhang S., Liu Y., Upton C., Kurien P.E., Li R., et al. Effectiveness and analysis of factors predictive of discharge to home in a 4-year cohort in a residential transitional care unit. Aging Med 2019;2:162–7. https://doi.org/10.1002/agm2.12076.

[94] Wasserman A, Thiessen M, Pooyania S. Factors associated with community versus personal care home discharges after inpatient stroke rehabilitation: the need for a pre-admission predictive model. Top Stroke Rehabil 2020;27:173–80. https://doi.org/10.1080/10749357.2019.1682369.

[95] Gozalo P, Leland NE, Christian TJ, Mor V, Teno JM. Volume matters: Returning home after hip fracture. Journal of the American Geriatrics Society 2015;63:2043–51. https://doi.org/10.1111/jgs.13677.

[96] Everink IHJ, Van Haastregt JCM, Van Hoof SJM, Schols JMGA, Kempen GIJM. Factors influencing home discharge after inpatient rehabilitation of older patients: A systematic review Health services research. BMC Geriatrics 2016;16. https://doi.org/10.1186/s12877-016-0187-4.

[97] Turcotte LA, Perlman CM, Fries BE, Hirdes JP. Clinical predictors of protracted length of stay in Ontario Complex Continuing Care hospitals. BMC Health Serv Res 2019;19:218. https://doi.org/10.1186/s12913-019-4024-2.

[98] Evans E, Gutman R, Resnik L, Zonfrillo MR, Lueckel SN, Kumar RG, et al. Successful community discharge among older adults with traumatic brain injury in skilled nursing facilities. Journal of Head Trauma Rehabilitation 2021;36:E186–98. https://doi.org/10.1097/HTR.0000000000000638.

[99] Kool J, Oesch P, Bachmann S. Predictors for living at home after geriatric inpatient rehabilitation: A prospective cohort study. J Rehabil Med 2017;49:185–90. https://doi.org/10.2340/16501977-2182.

[100] Simning A, Caprio TV, Seplaki CL, Conwell Y. Rehabilitation Providers’ Prediction of the Likely Success of the SNF-to-Home Transition Differs by Discipline. J AM MED DIR ASSOC 2019;20:492–6. https://doi.org/10.1016/j.jamda.2018.11.015.

[101] Bayer TA, Jiang L, Singh M, Kunicki ZJ, Browne JW, Nubong T, et al. Skilled Nursing Facility Rehabilitation Intensity and Successful Discharge in Persons with Dementia. J Am Med Dir Assoc 2024;25:105286. https://doi.org/10.1016/j.jamda.2024.105286.

[102] Mazzola P, Butto V, Elli S, Galluccio R, Domenici G, Stella V, et al. Frailty predicts failure to discharge patients home from a subacute-care unit: a 3-year Italian experience. Aging Clin Exp Res 2022;34:95–103. https://doi.org/10.1007/s40520-021-01908-w.

[103] Ohta R, Maeki N, Maniwa S, Miyakoshi K. Predicting factors of elderly patients’ discharge to home after rehabilitation in rural Japan: a retrospective cohort study. RURAL REMOTE HEALTH 2021;21:6406. https://doi.org/10.22605/RRH6406.

[104] Evans E, Kosar CM, Thomas KS. Positive Beliefs and the Likelihood of Successful Community Discharge From Skilled Nursing Facilities. vol. 102, 2021, p. 480–7. https://doi.org/10.1016/j.apmr.2020.09.375.

[105] Thornblade LW, Arbabi S, Flum DR, Qiu Q, Fawcett VJ, Davidson GH. Facility-Level Factors and Outcomes after Skilled Nursing Facility Admission for Trauma and Surgical Patients. J Am Med Dir Assoc 2018;19:70-76.e1. https://doi.org/10.1016/j.jamda.2017.08.007.

[106] Kushner DS, Peters KM, Johnson-Greene D. Evaluating Siebens Domain Management Model for inpatient rehabilitation to increase functional independence and discharge rate to home in geriatric patients. Arch Phys Med Rehabil 2015;96:1310–8. https://doi.org/10.1016/j.apmr.2015.03.011.

[107] O’Brien SR, Zhang N. Association Between Therapy Intensity and Discharge Outcomes in Aged Medicare Skilled Nursing Facilities Admissions. Arch Phys Med Rehabil 2018;99:107–15. https://doi.org/10.1016/j.apmr.2017.07.012.

[108] Wang S, Temkin-Greener H, Simning A, Konetzka RT, Cai S. Medicaid home- and community-based services and discharge from skilled nursing facilities. Health Serv Res 2021;56:1156–67. https://doi.org/10.1111/1475-6773.13690.
